# Atopic disease and inflammatory bowel disease: A bidirectional Mendelian randomization study

**DOI:** 10.1097/MD.0000000000040143

**Published:** 2024-10-18

**Authors:** Dongyuan Zheng, Qinke Xu, Yingchao Liu

**Affiliations:** a The Second Clinical Medical College of Zhejiang Chinese Medical University, Hangzhou, China; b Department of Gastroenterology, The Second Affiliated Hospital of Zhejiang Chinese Medical University, Hangzhou, China.

**Keywords:** atopic disease, causal effect, inflammatory bowel disease, Mendelian randomization

## Abstract

Observational studies have reported associations between atopic diseases, including allergic rhinitis (AR), asthma, atopic dermatitis (AD), and inflammatory bowel disease (IBD), but the causal relationship remains unknown. We utilized pooled data from genome-wide association studies, qualified instrumental variables were screened according to the 3 hypotheses of MR, and bidirectional causality between atopic diseases and IBD was assessed using 2-sample Mendelian randomization analysis (2SMR). The results of our study suggest that AR increased the risk of Crohn disease (CD) (IVW OR = 1.19, 95% CI = 1.02–1.39, *P* = .026), ulcerative colitis (UC) (IVW OR = 1.14, 95% CI = 1.01–1.29, *P* = .031) and overall IBD (IVW OR = 1.15, 95% CI = 1.03–1.28, *P* = .015); Asthma increased the risk of CD (IVW OR = 7.66, 95% CI = 1.58–37.20, *P* = .012), UC (IVW OR = 3.81, 95% CI = 1.09–13.32, *P* = .036) and overall IBD (IVW OR = 5.13, 95% CI = 1.48–17.70, *P* = .010); AD increased the risk of CD (IVW OR = 1.19, 95% CI = 1.02–1.39, *P* = .023) and overall IBD (IVW OR = 1.14, 95% CI = 1.03–1.28, *P* = .015) risk. In reverse causality, only CD increased the risk of AR (IVW OR = 1.02, 95% CI = 1.00–1.05, *P* = .031). This study shows that atopic diseases of AR and asthma are causally related to IBD and its subtypes, and AD is causally related to IBD (which may be attributed to CD). Of the reverse causality, only CD was causally related to AR.

## 1. Introduction

Inflammatory bowel disease (IBD) is an umbrella term that describes a group of chronic nonspecific inflammatory diseases of the gastrointestinal tract, including 2 major subtypes: ulcerative colitis (UC) and Crohn disease (CD).^[1]^ UC, which mainly affects the rectum and colon, tends to be a continuous inflammation affecting only the mucosal layer of the intestine and is mainly characterized by mucosal erosions and ulcer formation,^[2]^ whereas CD can involve any part of the intestine from the mouth to the anus and manifests itself in the form of strictures, ulcers, fistulas, and inflammation of the intestinal tract.^[3]^ As a multifactorial disease, the pathogenesis of IBD mainly involves complex interactions between environmental, genetic or microbial factors and the immune response.^[4–6]^ However, the exact pathogenesis remains unclear. Atopic disorders are a cluster of conditions closely associated with allergic responses, primarily encompassing bronchial asthma, allergic rhinitis (AR), and atopic dermatitis (AD). Studies have shown that atopic diseases have become a major global health problem, with their prevalence growing significantly in developing and industrialized countries.^[7]^ Asthma is mainly characterized by chronic inflammation of the airways and reversible airway narrowing. Patients often experience symptoms such as coughing, wheezing, and shortness of breath when exposed to allergens or encountering other irritants.^[8]^ Allergic rhinitis is mainly characterized by chronic inflammation of the nasal mucosa, leading to symptoms such as nasal congestion, sneezing, and runny nose.^[9]^ AD is mainly characterized by an inflammatory reaction of the skin, presenting as redness, dryness, and itching of the skin.^[10]^ Studies have shown that immune response, genetic, environmental and microbial factors play an equally important role in atopic diseases.^[11–13]^

Although atopic disease and IBD are 2 distinct conditions, a growing body of research suggests a correlation between the 2.^[14,15]^ A systematic review of 15 studies discovered a connection between asthma and both CD and UC. The link between CD and asthma remained consistent, but the association between UC and asthma seemed to be weaker.^[16]^ Nevertheless, a study conducted on a Korean population indicates that individuals with atopic disorders such as AD, AR, and asthma might not only be susceptible to IBD but also may be at progressively increased risk for IBD as the number of atopic disorders increases.^[17]^ Among children with IBD, there is a similar trend toward increased prevalence of asthma and AR. Although there is much evidence that atopic diseases and IBD interact,^[18,19]^ most of these studies are observational and may contain confounding variables or misclassifications, etc, and cannot be used to determine causality.

Mendelian randomization is a method of inferring causality that employs genetic variation to randomly allocate individuals into distinct groups, guaranteeing equity and randomness across experimental and control groups, eliminating potential sources of bias, and yielding more dependable and inclusive findings compared to observational studies.^[20]^ Hence, this research involved conducting MR analysis on GWAS data to enhance comprehension of the correlation between IBD and respiratory allergies, aiming to offer novel perspectives for enhancing patient diagnosis and treatment.

## 2. Materials and methods

**Figure 1.**
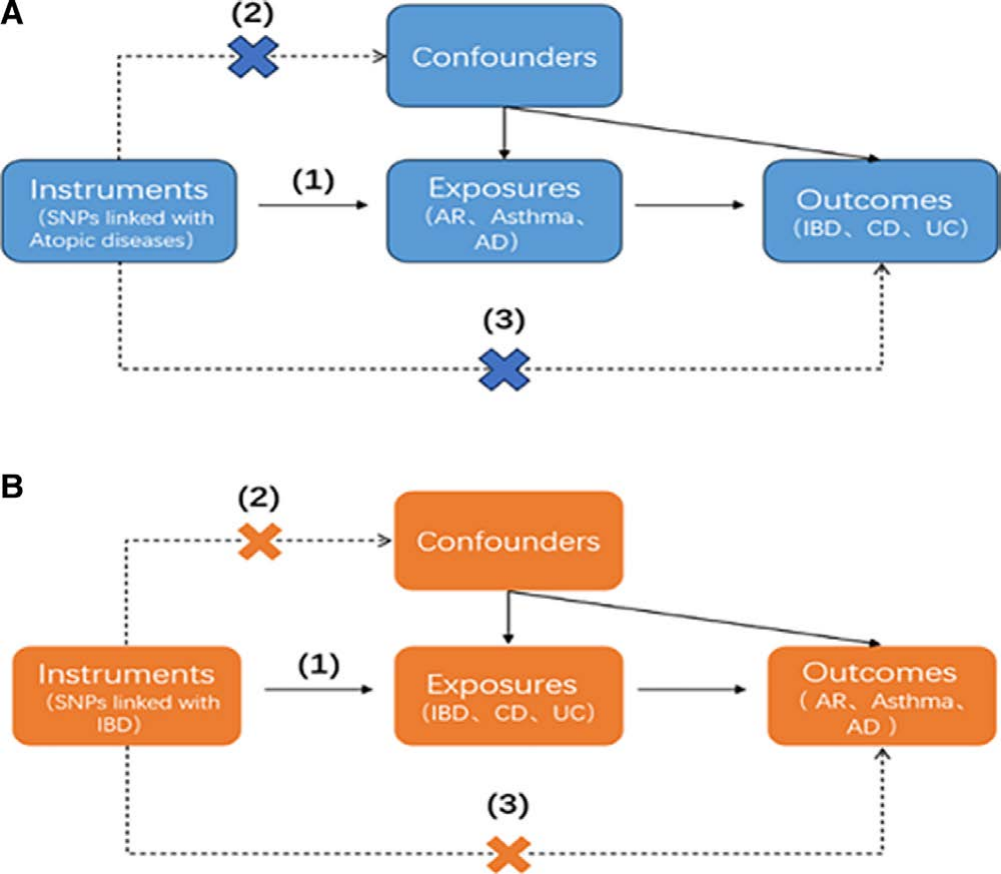
Diagram illustrating the main MR analysis assumptions. (A) shows the study investigating the genetic effect of Atopic diseases on IBD, while (B) shows the study investigating the genetic effect of IBD on Atopic diseases.

In a 2-sample MR study, a collection of multiple single nucleotide polymorphisms (SNPs) that represent genetic variation were examined as IVs. Three hypotheses were utilized in this study (Fig. 1): instrumental variables (IVs) have a direct and significant correlation with exposure; IVs are not influenced by any confounding factors; and IVs solely impact outcomes through exposure.^[20]^ MR analyses were performed to assess bidirectional causality between AR and IBD. Our study was designed and drafted according to the principles of the MR report, Strengthening the Reporting of Observational Studies in Epidemiology–Mendelian randomization (STROBE-MR).^[21]^

### 2.1. Data sources

To prevent the influence of population stratification, we collected summary data from studies exclusively conducted with individuals of European descent, encompassing all SNPs and their corresponding information. You can find summary statistics in Integrative Epidemiology Unit (IEU) Open GWAS database at (https://gwas.mrcieu.ac.uk/). A total of 96,486 individuals were involved in the study of IBD, with 90% European ancestry and 10% East Asian, Indian, or Iranian ancestry.^[22]^ Only individuals with European ancestry were included in the data analysis. The research involved a total of 12,882 individuals diagnosed with IBD and 21,770 individuals who were in good health and volunteered for the study. In the CD analysis, 5956 patients and 14,927 controls were included; in the UC analysis, 6968 patients and 20,464 controls were included. The other set of IBD data came from the FinnGen database, which provided IBD (5673 cases, 213,119 controls), as well as CD (2056 cases, 210,300 controls) and UC (4320 cases, 210,300 controls), also available in the IEU GWAS database.

In addition, we explored the publicly accessible IEU GWAS database for any genome-wide association studies (GWASs) associated with AR, Asthma, and AD. The specific number of cases and controls for AR was not provided by the database; however, there were 38,791 cases and 297,991 controls in relation to Asthma, and 22,474 cases and 774,187 controls in relation to AD.

Ethical approval or patient consent was not necessary for this study as the data were obtained from previously published studies available in the public domain.

### 2.2. Selection of instrumental variables

The selection of IVs was based on a series of quality control steps. First, SNPs were screened from atopic disease data (*P* < 5 × 10^−8^) with a regional range (kb) of 10,000 for linkage disequilibrium (LD) and a threshold of 0.01 for the LD parameter (r^2^). Weak IVs with *F*-test value < 10 were removed. Palindrome SNPs were further excluded.

We searched the phenotype scanner database for any confounding variables and outcomes (*P* < 5 × 10^−8^) related to all SNPs associated with exposure. Genetic IVs were manually eliminated to ensure their independence from outcome and confounders.

### 2.3. Statistical analysis

All analyses were performed in RStudio version 4.3.1, using the “TwoSampleMR” and “MendelianRandomization” packages for data analysis.

We used various MR methods in our study to determine that atopic disease is causally related to IBD, including inverse variance weighting (IVW), MR-Egger regression, simple model, weighted median, weighted model, contamination mixture method, where IVW was the most common method. IVW is fitted using the inverse of the variance of the IVs when all IVs satisfy the 3 conditions and is an unbiased estimation^[23]^; MR-Egger regression uses the inverse of the variance of the endings in a weighted computation, incorporates an intercept term in the regression, takes into account multicollinearity, and does not require that the straight line passes through the origin^[24]^; Weighted median has the advantage of giving consistent effect estimates even when the null instrumental variable is close to 50%; Weighted median is slightly less effective because each SNP receives the same weight in the statistical analysis,^[25]^ whereas more precise SNPs in the Weighted mode receive more weight.^[26]^ The contamination mixture method is good at analyzing hundreds of IVs and can provide causal estimates without invalid instruments.^[27]^ In this study, IVW results were used as the primary outcome. We used Cochran Q test to assess heterogeneity, and if the *P*-value was <0.05 (*P < *.05), it indicates the presence of heterogeneity. To assess the presence of horizontal pleiotropy, we used the MR-Egger intercept test, where a significant intercept (*P* < .05) indicates the presence of pleiotropy, and the results should be interpreted with caution.^[24]^

## 3. Results

### 3.1. Selection of instrumental variables associated with atopic diseases and inflammatory bowel disease

The number of SNPs ranged from 31 to 143, after quality control steps by LD effects and palindromic. The *F*-statistic of SNPs ranges from 20.8 to 424, indicating that each SNP revealed adequate validity. The detailed information for each SNP and its *F*-statistic value were shown in Tables S1 to S18, Supplemental Digital Content, http://links.lww.com/MD/N766.

### 3.2. Causal effects of atopic diseases on inflammatory bowel disease and its subtypes

In order to examine the harmful effect of atopic conditions on IBD, 177 SNPs were initially chosen as IVs for AR, which were significant at the genome-wide level (*P* < 5 × 10^−8^) and had independent inheritance (r2 < 0.01 and distance > 10,000 kb) when compared to 110392006 SNPs. By querying the PhenoScanner V2 database,^[28]^ 11 SNPs associated with at least 1 IBD-associated trait (such as rs10066308) were excluded. A total of 25 possible SNPs were identified as having palindromic characteristics. In the end, a total of 141 SNPs were authorized for conducting MR analysis to assess the detrimental effect of AR on the risk of IBD. Similarly, according to the above process and criteria, 143 and 141 SNPs were used for the MR analysis of the pathogenic impact of AR on CD and AR on UC risk; 76, 76, and 74 SNPs were screened for the analysis of asthma on IBD, CD, and UC risk, respectively; due to the lack of access to a sufficiently large number of SNPs in the analysis of the risk of AD on IBD and its subtypes, we adjusted for significance (*P* < 5 × 10^−6^), and finally 61, 61, and 63 SNPs were used in the analysis of AD on IBD, CD, and UC risk, respectively. The *F*-statistics for all IVs were > 10, indicating no bias from weak IVs.

**Figure 2.**
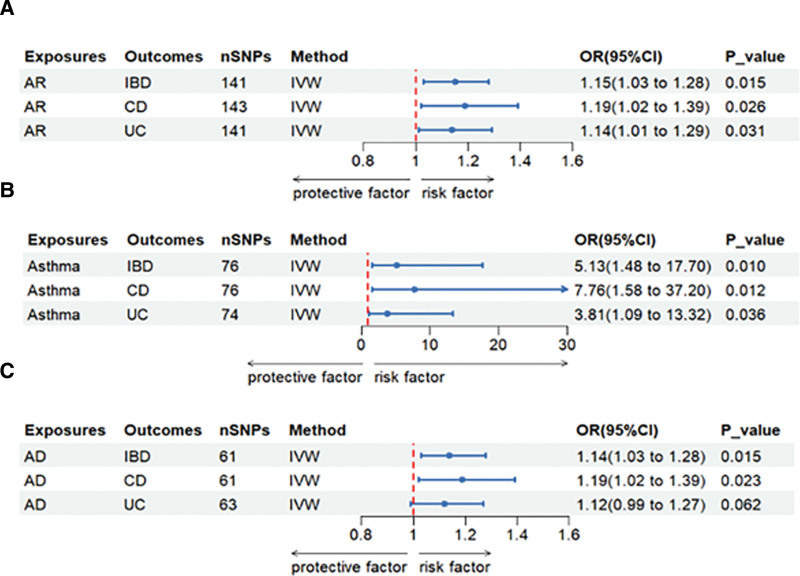
Causal estimates given as odds ratios (ORs) and 95% confidence intervals for the effect of atopic dermatitis on inflammatory bowel disease as a whole, on Crohn disease, and ulcerative colitis. (A) Mendelian randomization results of causal effects between AR and IBD (including CD and UC). (B) Mendelian randomization results of causal effects between Asthma and IBD (including CD and UC). (C) Mendelian randomization results of causal effects between AD and IBD (including CD and UC).Estimates were obtained from the inverse-variance weighted method.

Figure S1, Supplemental Digital Content (http://links.lww.com/MD/N765), shows the MR estimates for the various methods. Cochran *Q* test showed heterogeneity (*P* < .05) (Table S19, Supplemental Digital Content, http://links.lww.com/MD/N767). Therefore, we used the IVW method in the random effects model. A significant causal relationship was observed between atopic disease and IBD (Fig. 2A). AR was found to be associated with IBD (IVW OR = 1.15, 95% CI = 1.03–1.28, *P* = .015), as well as CD (IVW OR = 1.19, 95% CI = 1.02–1.39, *P* = .026). However, when analyzing AR and UC, the predictive directions of the Simple mode and Weighted model differed from the remaining 3 methods. To ensure robust outcomes, we supplemented the causal estimates using the contamination mixture method for AR and UC (IVW OR = 1.14, 95% CI = 1.01–1.29, *P* = .031) (Figure S9, Supplemental Digital Content, http://links.lww.com/MD/N765). Figure 2B illustrates the causal relationship between asthma and IBD (IVW OR = 5.13, 95% CI = 1.48–17.70, *P* = .010), as well as CD (IVW OR = 7.66, 95% CI = 1.58–37.20, *P* = .012), and UC (IVW OR = 3.81, 95% CI = 1.09–13.32, *P* = .036). Furthermore, Figure 2C demonstrates the causal relationship between AD and IBD (IVW OR = 1.14, 95% CI = 1.03–1.28, *P* = .015), CD (IVW OR = 1.19, 95% CI = 1.02–1.39, *P* = .023), and UC (IVW OR = 1.12, 95% CI = 0.99–1.27, *P* = .062), which did not show sufficient significance in terms of causality. The direction of other MR methods, such as Weighted median’s results and MR-Egger results, was consistent. Since the horizontal multivariate test did not demonstrate statistical significance (*P* > .05 for the MR-Egger intercept), estimating causal effects using IVW estimation instead of MR-Egger regression might be more reliable. Regarding this matter, we supported the IVW findings and evaluated the causal impact of atopic illness on IBD.

### 3.3. Causal impact of inflammatory bowel disease and its subtypes on atopic diseases

Atopic disease outcomes were demonstrated using IBD and its subtypes as the exposure factors in reverse causality. For IBD and asthma, a total of 5646, and 33 SNPs were found as IVs for the 3 distinct outcomes of IBD, CD, and UC, respectively. However, in the case of IBD and AD, the number of SNPs was insufficient, so we adjusted the significance level (*P* < 5 × 10^−6^) and identified 4533, and 34 SNPs as IVs for IBD, CD, and UC, respectively. Similarly, in the context of IBD with AR, we also encountered a lack of adequate SNPs. Therefore, we adjusted the significance level (*P* < 5 × 10^−6^) and discovered 42, 31, and 31 SNPs as IVs for the 3 different outcomes of IBD, CD, and UC, respectively.

The *F*-statistic for all IVs was > 10, indicating the absence of bias from weak IVs. Once again, because of the existence of diversity, we employed a random effects approach. Figure S2, Supplemental Digital Content (http://links.lww.com/MD/N765), shows the MR estimates for the various methods. Moreover, Figure S10, Supplemental Digital Content (http://links.lww.com/MD/N765) shows a forest plot of the causal relationship between IBD and atopic disease. Based on the IVW approach, there was no observed link between IBD and asthma (IVW OR = 1.00, 95% CI = 1.00–1.01, *P* = .198). The MR-Egger method indicated an opposite direction, suggesting that the validity of causality is not supported for Crohn (IVW OR = 1.00, 95% CI = 1.00–1.01, *P* = .046). There was no evidence of causality for UC (IVW OR = 1.00, 95% CI = 1.00–1.00, *P* = .919).

Furthermore, no evidence was found indicating a connection between IBD (including Crohn and UC) and AD (IVW OR = 1.03, 95% CI = 0.99–1.07, *P* = .108; IVW OR = 1.01, 95% CI = 0.99–1.03, *P* = .360; IVW OR = 1.01, 95% CI = 0.99–1.04, *P* = .293).

Allergic rhinitis was not causally affected by IBD and UC (IVW OR = 1.02, 95% CI = 0.99–1.05, *P* = .107; IVW OR = 1.01, 95% CI = 0.98–1.04, *P* = .483;), whereas CD was found to have a causal effect on AR (IVW OR = 1.02, 95% CI = 1.00–1.05, *P* = .031).

All MR-Egger regressions except UC on asthma produced negative results (MR-Egger intercept *P* > .05), indicating unbiased horizontal pleiotropy (Table S19, Supplemental Digital Content, http://links.lww.com/MD/N767). Moreover, the forest plot and funnel plot both conformed to the characteristics of the positive outcomes provided by the MR analysis, and the Leave-One-Out analysis proved the robustness of the outcome (Figures S3–S8, Supplemental Digital Content, http://links.lww.com/MD/N765).

## 4. Discussion

We evaluated the observation in MR analyses utilizing the GWAS study database that asthma and AR elevate the likelihood of developing IBD and its subcategories, while AD raises the risk of IBD, potentially due to CD. In reverse causality, CD increased the risk of AR.

Observational studies indicate a frequent co-occurrence of asthma and IBD, as demonstrated by a Canadian study.^[29]^ The study revealed that the annual average incidence of CD and UC among individuals with asthma was 23.1 per 100,000 and 8.1 per 100,000 respectively, significantly surpassing the incidence rates in the general population. A Danish national study stated^[19]^ that asthma in childhood similarly increases the risk of IBD. According to a case-control study^[30]^ in the Nordic region, it was suggested that individuals with IBD, particularly those with UC and women, have a higher occurrence of asthma. However, in our study, there was no increased risk of asthma among patients with IBD, which may be due to geographical differences as well as differences in the nature of the study. Interestingly, an MR study stated^[31]^ that there is a negative correlation between asthma and CD, but more studies are needed to confirm this.

AR is also often associated with IBD, and many previous studies have shown^[14,17]^ that AR increases the risk of developing IBD. In a reverse causality, a meta-analysis that included 11 case-control and cohort studies found that people with IBD had a higher probability of developing AR, in which 5838 of 65,687 people with IBD suffered from AR. The control group consisted of 345,176 participants who did not have IBD, out of which only 24,625 individuals developed AR.^[32]^ Our study found that CD increases the risk of AR, however, UC does not seem to increase the risk of AR, and the results that account for this discrepancy may be related to the different pathogenesis of these 2 subtypes.

There is also a connection between AD and IBD, and research^[33]^ has demonstrated that individuals with AD are more likely to develop CD, although there seems to be no link between AD and UC. A study^[34]^ involving both children and adults with AD found that pediatric patients with AD had a 44% higher chance of developing IBD and a 74% higher chance of developing CD, which increased with the severity of AD. However, except for those with severe AD, they did not show an increased risk of UC. As AD worsened, adults experienced a 34% higher risk of IBD, a 36% higher risk of CD, and a 32% higher risk of UC. Our study suggests that AD increases the risk of CD but not UC, which is consistent with some of the previous findings.

The pathogenesis of atopic diseases and IBD share intriguing intersections, primarily due to the commonality of immune system hyperactivation as a pivotal factor. Research has discovered that Th17 cells have a crucial role in the development of atopic diseases. These cells are notably elevated in the bloodstream of individuals with asthma, AD, and AR. They generate cytokines like IL-17 and TNF-α, contributing to the inflammatory response. Excessive inflammation can worsen symptoms and harm tissues.^[35–37]^ Additionally, Th17 cells are implicated in the pathogenesis of IBD. Normally, they play a vital role in safeguarding mucosal and epithelial tissues against external microbial infections. However, when immune dysregulation occurs, Th17 cells proliferate abnormally and produce large amounts of pro-inflammatory cytokines, inducing an abnormal immune response and leading to the development of IBD. A recent discovery suggests that IL-36 cytokines may serve as a novel focus for addressing IBD and atopic diseases.^[38]^ Increased expression of IL-36α and IL-36γ was detected in colon biopsy tissues of patients with active IBD.^[39,40]^ Additionally, IL-36R-/- mice subjected to acute dextran sodium sulfate-induced (DSS-induced) colitis exhibited up-regulation of IL-36α and IL-36γ.^[41]^ Furthermore, IL-36R signaling enhances the Th1 response while suppressing the Th17 response. Administration of IL-38, an IL-36 inhibitor, mitigated the severity of acute colitis induced by DSS in mice. Elevated mRNA and protein expression of IL-36 was likewise found in the serum of AR patients, and an experimental study found that IL-36α promoted Th17 cell differentiation and function in an AR mouse model, and that anti-IL-36α treatment significantly attenuated symptoms, with a reduction in Th17 cell infiltration and down-regulation of Th17 cytokine expression.^[42]^ Studies have shown that alterations in the microbiome are similarly associated with the pathogenesis of both. IBD^[5]^ is caused by changes in the gut microbiome, which serves as a crucial controller of the immune system. In patients with AD, microbiome diversity is reduced,^[43]^and further studies have suggested that alterations in the gut microbiome often accompany these patients, and that immune disorders caused by ecological dysregulation of the gut-skin axis contribute to the development of AD.^[44]^ The gut microbiome also plays a significant role in the communication between the gut and lungs. It is a key factor in the formation of Th1 and Th2 cells, and the use of antimicrobial agents delays the maturation of Th1 cells and decreases the Th2 response. This, in turn, raises the likelihood of developing asthma and other allergic conditions.^[45]^ Studies have reported the importance of observing the dynamics of tight junction proteins (TJ) in IBD and allergic diseases, where TJ dysfunction increases paracellular permeability, leading to an increase in flux between TJ. Chronic inflammation of the lungs, skin, and intestines is also a result of immune cell activation caused by dysfunction in epithelial TJ. The relationship between atopic disease and IBD is complex and intertwined in terms of pathogenesis, and additional research is necessary to acquire a more profound comprehension of their connection.

The primary advantage of this study is that, as far as we know, we evaluated for the initial occasion the cause-and-effect influence of atopic illness on the formation of IBD and vice versa by employing a 2-sample MR methodology. The advantage of this approach is that it is less susceptible to confounding, reverse causality, and exposure to undifferentiated measurements than observational studies.^[46]^ In addition, we conducted stratified studies. CD and UC are 2 subtypes of IBD. Although previous research has shown that CD and UC have similar positive outcomes, their validity differs. Our study found that in AR, UC and CD have different outcomes.

This study has some limitations. Although we selected data exclusively from the European cohort, significant heterogeneity was observed. Moreover, the IVs witnessed in European communities cannot be equated with those observed in non-European communities because of dissimilarities in genetic vulnerability and environmental stimuli between Eastern and Western populations.^[47]^ Moreover, there are variations in genetic vulnerability and environmental stimuli among Eastern and Western populations. However, recent epidemiologic investigations have shown that non-European populations are emerging as new victims of IBD and atopic disease.

## 5. Conclusions

To summarize, our findings indicate that both AR and asthma elevate the likelihood of developing IBD and its subcategories. Additionally, AD is associated with an increased risk of IBD, potentially linked to CD. In reverse causation, CD increases the risk of AR. To ensure early diagnosis and initiate targeted therapy, physicians must recognize the increased susceptibility to IBD in patients with atopic conditions. Further research is needed to examine the pathophysiologic mechanisms behind this relationship.

## Acknowledgments

The authors thank the studies or consortiums referenced and included in the present analysis for providing public datasets.

## Author contributions

**Data curation:** Dongyuan Zheng.

**Formal analysis:** Yingchao Liu.

**Investigation:** Dongyuan Zheng.

**Methodology:** Dongyuan Zheng, Qinke Xu.

**Project administration:** Yingchao Liu.

**Supervision:** Yingchao Liu.

**Validation:** Dongyuan Zheng.

**Visualization:** Dongyuan Zheng, Qinke Xu.

**Writing – original draft:** Dongyuan Zheng, Qinke Xu.

**Writing – review & editing:** Qinke Xu.

## Supplementary Material


