# Supplementary material for: Atopic disease and inflammatory bowel disease: A bidirectional Mendelian randomization study
Source: Medicine (Baltimore). 2024 Oct 18;103(42):e40143. doi: 10.1097/MD.0000000000040143 (PMC11495711; doi:10.1097/MD.0000000000040143)
Supplement: Supplementary file 1 [file medi-103-e40143-s001.docx]

**Table1 SNPs selected as IVs for IBD**

| **exposure** | **SNP** | **effect_allele** | **other_allele** | **chr** | **beta** | **se** | **samplesize** | **pval** | **F_statistic** |
| --- | --- | --- | --- | --- | --- | --- | --- | --- | --- |
| AR | rs10131567 | T | G | 14 | 3.86E-02 | 5.83E-03 | 407746 | 3.77E-11 | 4.37E+01 |
| AR | rs10244416 | T | C | 7 | -4.89E-02 | 5.24E-03 | 407746 | 9.87E-21 | 8.72E+01 |
| AR | rs10245867 | T | G | 7 | 4.55E-02 | 5.56E-03 | 407746 | 3.14E-16 | 6.67E+01 |
| AR | rs1032841 | G | A | 2 | -3.25E-02 | 5.79E-03 | 407746 | 2.03E-08 | 3.15E+01 |
| AR | rs10468514 | G | A | 17 | 3.11E-02 | 5.35E-03 | 407746 | 6.04E-09 | 3.38E+01 |
| AR | rs10485354 | G | A | 6 | -3.05E-02 | 5.27E-03 | 407746 | 7.09E-09 | 3.35E+01 |
| AR | rs10519068 | A | G | 15 | -7.12E-02 | 7.82E-03 | 407746 | 8.50E-20 | 8.29E+01 |
| AR | rs1057258 | T | C | 2 | -4.92E-02 | 6.82E-03 | 407746 | 5.47E-13 | 5.20E+01 |
| AR | rs10791824 | G | A | 11 | 5.14E-02 | 5.29E-03 | 407746 | 2.47E-22 | 9.45E+01 |
| AR | rs10795672 | A | G | 10 | 3.29E-02 | 5.32E-03 | 407746 | 6.43E-10 | 3.82E+01 |
| AR | rs10910093 | T | C | 1 | -5.76E-02 | 7.67E-03 | 407746 | 6.21E-14 | 5.63E+01 |
| AR | rs10912564 | T | C | 1 | 4.79E-02 | 5.69E-03 | 407746 | 3.83E-17 | 7.09E+01 |
| AR | rs1102737 | A | G | 1 | -5.29E-02 | 8.47E-03 | 407746 | 4.00E-10 | 3.91E+01 |
| AR | rs111267073 | T | C | 5 | -9.69E-02 | 1.70E-02 | 407746 | 1.20E-08 | 3.25E+01 |
| AR | rs112008378 | A | C | 10 | -3.27E-02 | 5.37E-03 | 407746 | 1.16E-09 | 3.70E+01 |
| AR | rs11255753 | T | G | 10 | 3.29E-02 | 5.84E-03 | 407746 | 1.85E-08 | 3.17E+01 |
| AR | rs113377887 | T | C | 13 | 4.72E-02 | 6.40E-03 | 407746 | 1.59E-13 | 5.45E+01 |
| AR | rs114695117 | A | C | 1 | -9.40E-02 | 1.61E-02 | 407746 | 5.92E-09 | 3.39E+01 |
| AR | rs1150658 | T | C | 6 | 4.48E-02 | 6.13E-03 | 407746 | 2.70E-13 | 5.34E+01 |
| AR | rs115288876 | A | G | 1 | 1.33E-01 | 1.30E-02 | 407746 | 1.45E-24 | 1.05E+02 |
| AR | rs11626205 | A | G | 14 | 3.45E-02 | 5.72E-03 | 407746 | 1.63E-09 | 3.64E+01 |
| AR | rs117137535 | A | G | 9 | 1.22E-01 | 1.80E-02 | 407746 | 9.32E-12 | 4.65E+01 |
| AR | rs11742240 | T | G | 5 | -9.17E-02 | 5.86E-03 | 407746 | 3.21E-55 | 2.45E+02 |
| AR | rs117710327 | A | C | 19 | -9.32E-02 | 1.07E-02 | 407746 | 2.47E-18 | 7.63E+01 |
| AR | rs12068304 | G | A | 1 | 4.99E-02 | 7.13E-03 | 407746 | 2.74E-12 | 4.89E+01 |
| AR | rs1214598 | A | G | 1 | -5.49E-02 | 5.37E-03 | 407746 | 1.63E-24 | 1.04E+02 |
| AR | rs12152276 | G | A | 3 | -5.65E-02 | 9.53E-03 | 407746 | 3.09E-09 | 3.51E+01 |
| AR | rs12185242 | C | A | 17 | 3.36E-02 | 5.25E-03 | 407746 | 1.68E-10 | 4.08E+01 |
| AR | rs12282231 | T | C | 11 | 6.83E-02 | 1.05E-02 | 407746 | 7.83E-11 | 4.23E+01 |
| AR | rs12365699 | A | G | 11 | -5.06E-02 | 7.04E-03 | 407746 | 7.03E-13 | 5.15E+01 |
| AR | rs12440045 | C | A | 15 | 4.48E-02 | 5.29E-03 | 407746 | 2.51E-17 | 7.17E+01 |
| AR | rs1260294 | C | T | 12 | -3.79E-02 | 6.54E-03 | 407746 | 6.83E-09 | 3.36E+01 |
| AR | rs12625547 | G | T | 20 | -4.30E-02 | 6.92E-03 | 407746 | 5.10E-10 | 3.86E+01 |
| AR | rs12948911 | T | C | 17 | -4.25E-02 | 6.68E-03 | 407746 | 1.95E-10 | 4.05E+01 |
| AR | rs16858573 | C | T | 2 | -5.54E-02 | 7.92E-03 | 407746 | 2.62E-12 | 4.90E+01 |
| AR | rs1850984 | A | G | 2 | 3.60E-02 | 6.19E-03 | 407746 | 6.01E-09 | 3.38E+01 |
| AR | rs1904522 | A | G | 4 | 6.47E-02 | 5.54E-03 | 407746 | 1.57E-31 | 1.36E+02 |
| AR | rs2070901 | T | G | 1 | 4.36E-02 | 5.89E-03 | 407746 | 1.29E-13 | 5.49E+01 |
| AR | rs2074585 | A | G | 15 | -5.31E-02 | 5.23E-03 | 407746 | 2.74E-24 | 1.03E+02 |
| AR | rs2190097 | T | C | 7 | -4.45E-02 | 6.12E-03 | 407746 | 3.73E-13 | 5.28E+01 |
| AR | rs2228145 | C | A | 1 | 3.86E-02 | 5.29E-03 | 407746 | 3.18E-13 | 5.31E+01 |
| AR | rs2235864 | A | G | 20 | 3.43E-02 | 6.03E-03 | 407746 | 1.34E-08 | 3.23E+01 |
| AR | rs2255088 | C | T | 10 | 3.37E-02 | 5.57E-03 | 407746 | 1.44E-09 | 3.66E+01 |
| AR | rs2455101 | G | A | 1 | -3.97E-02 | 6.67E-03 | 407746 | 2.53E-09 | 3.55E+01 |
| AR | rs249677 | A | C | 5 | 3.46E-02 | 5.43E-03 | 407746 | 1.88E-10 | 4.06E+01 |
| AR | rs250298 | A | G | 5 | -3.22E-02 | 5.28E-03 | 407746 | 1.08E-09 | 3.72E+01 |
| AR | rs2516518 | A | G | 6 | 8.79E-02 | 8.72E-03 | 407746 | 6.45E-24 | 1.02E+02 |
| AR | rs2723197 | A | G | 2 | -3.04E-02 | 5.27E-03 | 407746 | 7.42E-09 | 3.34E+01 |
| AR | rs2766680 | A | G | 20 | -5.51E-02 | 6.54E-03 | 407746 | 3.57E-17 | 7.10E+01 |
| AR | rs2846072 | C | T | 11 | -7.17E-02 | 1.24E-02 | 407746 | 7.64E-09 | 3.34E+01 |
| AR | rs2961920 | A | C | 5 | 4.76E-02 | 6.16E-03 | 407746 | 1.17E-14 | 5.96E+01 |
| AR | rs301806 | T | C | 1 | 3.82E-02 | 5.30E-03 | 407746 | 5.46E-13 | 5.20E+01 |
| AR | rs3024665 | T | C | 16 | 6.31E-02 | 1.10E-02 | 407746 | 8.93E-09 | 3.31E+01 |
| AR | rs3024971 | G | T | 12 | -8.31E-02 | 8.46E-03 | 407746 | 9.23E-23 | 9.64E+01 |
| AR | rs3135020 | A | C | 6 | -8.92E-02 | 8.10E-03 | 407746 | 3.16E-28 | 1.21E+02 |
| AR | rs3213567 | C | T | 12 | -3.81E-02 | 5.24E-03 | 407746 | 3.34E-13 | 5.30E+01 |
| AR | rs34119476 | G | A | 2 | -3.03E-02 | 5.31E-03 | 407746 | 1.12E-08 | 3.26E+01 |
| AR | rs34231012 | T | C | 1 | -3.45E-02 | 5.98E-03 | 407746 | 8.22E-09 | 3.32E+01 |
| AR | rs34290285 | A | G | 2 | -9.06E-02 | 5.99E-03 | 407746 | 9.16E-52 | 2.29E+02 |
| AR | rs35928002 | G | A | 19 | 3.37E-02 | 5.85E-03 | 407746 | 8.16E-09 | 3.32E+01 |
| AR | rs3730783 | G | A | 18 | -3.26E-02 | 5.33E-03 | 407746 | 9.53E-10 | 3.74E+01 |
| AR | rs3918226 | T | C | 7 | -7.06E-02 | 9.69E-03 | 407746 | 3.22E-13 | 5.31E+01 |
| AR | rs3985697 | C | T | 18 | -4.37E-02 | 6.86E-03 | 407746 | 1.92E-10 | 4.06E+01 |
| AR | rs41190 | A | C | 16 | 3.46E-02 | 6.16E-03 | 407746 | 1.90E-08 | 3.16E+01 |
| AR | rs4369774 | C | A | 18 | 3.23E-02 | 5.27E-03 | 407746 | 8.80E-10 | 3.76E+01 |
| AR | rs4379740 | C | T | 10 | -6.74E-02 | 6.77E-03 | 407746 | 2.29E-23 | 9.92E+01 |
| AR | rs45613035 | C | T | 4 | 8.67E-02 | 8.77E-03 | 407746 | 4.40E-23 | 9.79E+01 |
| AR | rs4686466 | G | A | 3 | -4.13E-02 | 6.56E-03 | 407746 | 3.10E-10 | 3.96E+01 |
| AR | rs4705869 | T | C | 5 | 4.88E-02 | 8.63E-03 | 407746 | 1.58E-08 | 3.20E+01 |
| AR | rs4769975 | A | G | 13 | -7.39E-02 | 1.34E-02 | 407746 | 3.58E-08 | 3.04E+01 |
| AR | rs4771332 | C | T | 13 | 3.13E-02 | 5.67E-03 | 407746 | 3.42E-08 | 3.05E+01 |
| AR | rs4939489 | T | C | 11 | -3.30E-02 | 5.35E-03 | 407746 | 7.31E-10 | 3.79E+01 |
| AR | rs4948563 | A | G | 10 | 3.45E-02 | 5.97E-03 | 407746 | 7.57E-09 | 3.34E+01 |
| AR | rs4995269 | G | A | 15 | 3.53E-02 | 5.54E-03 | 407746 | 1.82E-10 | 4.07E+01 |
| AR | rs5029949 | G | A | 6 | -9.37E-02 | 1.48E-02 | 407746 | 2.69E-10 | 3.99E+01 |
| AR | rs519973 | A | G | 3 | 5.09E-02 | 5.52E-03 | 407746 | 2.80E-20 | 8.51E+01 |
| AR | rs56183820 | T | C | 5 | 4.36E-02 | 6.68E-03 | 407746 | 6.37E-11 | 4.27E+01 |
| AR | rs563118370 | G | A | 11 | 1.04E-01 | 1.89E-02 | 407746 | 3.88E-08 | 3.02E+01 |
| AR | rs56854963 | T | C | 16 | -5.48E-02 | 8.59E-03 | 407746 | 1.75E-10 | 4.07E+01 |
| AR | rs5743618 | A | C | 4 | -1.29E-01 | 6.26E-03 | 407746 | 3.65E-94 | 4.24E+02 |
| AR | rs57453899 | A | G | 7 | 3.95E-02 | 6.47E-03 | 407746 | 1.02E-09 | 3.73E+01 |
| AR | rs59183580 | G | A | 11 | -4.93E-02 | 7.78E-03 | 407746 | 2.37E-10 | 4.01E+01 |
| AR | rs5997389 | A | G | 22 | 5.74E-02 | 9.60E-03 | 407746 | 2.19E-09 | 3.58E+01 |
| AR | rs60946162 | T | C | 3 | 3.49E-02 | 5.27E-03 | 407746 | 3.73E-11 | 4.37E+01 |
| AR | rs61192126 | C | T | 3 | -3.60E-02 | 5.77E-03 | 407746 | 4.49E-10 | 3.89E+01 |
| AR | rs61811906 | G | A | 1 | -2.94E-02 | 5.33E-03 | 407746 | 3.49E-08 | 3.04E+01 |
| AR | rs61813293 | T | G | 1 | -4.54E-02 | 7.44E-03 | 407746 | 1.01E-09 | 3.73E+01 |
| AR | rs61816766 | C | T | 1 | 1.48E-01 | 1.50E-02 | 407746 | 4.22E-23 | 9.80E+01 |
| AR | rs61839660 | T | C | 10 | 1.07E-01 | 8.74E-03 | 407746 | 3.36E-34 | 1.49E+02 |
| AR | rs61907710 | T | C | 11 | -4.76E-02 | 6.31E-03 | 407746 | 4.64E-14 | 5.69E+01 |
| AR | rs61938962 | T | C | 12 | 5.56E-02 | 5.51E-03 | 407746 | 5.32E-24 | 1.02E+02 |
| AR | rs62064086 | G | A | 17 | 5.13E-02 | 7.50E-03 | 407746 | 7.65E-12 | 4.69E+01 |
| AR | rs62117160 | A | G | 19 | -7.50E-02 | 1.24E-02 | 407746 | 1.70E-09 | 3.63E+01 |
| AR | rs62162287 | C | T | 2 | -3.93E-02 | 5.34E-03 | 407746 | 1.69E-13 | 5.43E+01 |
| AR | rs62328536 | A | C | 4 | -3.80E-02 | 5.48E-03 | 407746 | 3.90E-12 | 4.82E+01 |
| AR | rs62375554 | A | G | 5 | 8.34E-02 | 1.42E-02 | 407746 | 4.11E-09 | 3.46E+01 |
| AR | rs62379371 | A | G | 5 | -8.53E-02 | 1.24E-02 | 407746 | 5.10E-12 | 4.76E+01 |
| AR | rs62623446 | T | C | 12 | 6.80E-02 | 1.01E-02 | 407746 | 1.73E-11 | 4.53E+01 |
| AR | rs6594499 | A | C | 5 | -9.08E-02 | 5.24E-03 | 407746 | 3.21E-67 | 3.00E+02 |
| AR | rs6596860 | G | A | 6 | -3.33E-02 | 6.07E-03 | 407746 | 4.14E-08 | 3.01E+01 |
| AR | rs66632892 | A | G | 9 | 3.84E-02 | 5.98E-03 | 407746 | 1.36E-10 | 4.12E+01 |
| AR | rs67385163 | G | A | 12 | 3.27E-02 | 5.50E-03 | 407746 | 2.72E-09 | 3.54E+01 |
| AR | rs6938239 | A | G | 6 | 4.14E-02 | 7.53E-03 | 407746 | 3.83E-08 | 3.02E+01 |
| AR | rs709498 | G | A | 3 | 3.07E-02 | 5.26E-03 | 407746 | 5.50E-09 | 3.40E+01 |
| AR | rs71368508 | A | C | 17 | -1.38E-01 | 1.84E-02 | 407746 | 6.90E-14 | 5.61E+01 |
| AR | rs7140939 | G | A | 14 | -4.13E-02 | 5.41E-03 | 407746 | 2.34E-14 | 5.82E+01 |
| AR | rs72701682 | G | A | 9 | 3.33E-02 | 5.79E-03 | 407746 | 8.44E-09 | 3.32E+01 |
| AR | rs72702772 | A | G | 14 | -4.83E-02 | 6.55E-03 | 407746 | 1.67E-13 | 5.44E+01 |
| AR | rs73196739 | T | C | 3 | -4.75E-02 | 6.99E-03 | 407746 | 1.14E-11 | 4.61E+01 |
| AR | rs73285914 | G | A | 20 | -4.89E-02 | 8.60E-03 | 407746 | 1.31E-08 | 3.23E+01 |
| AR | rs73390208 | T | C | 6 | -8.66E-02 | 5.92E-03 | 407746 | 1.75E-48 | 2.14E+02 |
| AR | rs7410883 | C | T | 1 | -4.54E-02 | 8.31E-03 | 407746 | 4.76E-08 | 2.98E+01 |
| AR | rs7606480 | A | G | 2 | -3.16E-02 | 5.28E-03 | 407746 | 2.08E-09 | 3.59E+01 |
| AR | rs7606520 | C | A | 2 | 3.14E-02 | 5.24E-03 | 407746 | 1.95E-09 | 3.60E+01 |
| AR | rs7625643 | G | A | 3 | 3.66E-02 | 5.33E-03 | 407746 | 6.42E-12 | 4.72E+01 |
| AR | rs76282174 | T | C | 11 | 1.08E-01 | 1.60E-02 | 407746 | 1.43E-11 | 4.56E+01 |
| AR | rs7735519 | A | C | 5 | 1.23E-01 | 7.40E-03 | 407746 | 2.45E-62 | 2.78E+02 |
| AR | rs77665773 | T | C | 5 | 6.56E-02 | 1.11E-02 | 407746 | 3.88E-09 | 3.47E+01 |
| AR | rs77795650 | A | G | 6 | 7.20E-02 | 1.22E-02 | 407746 | 3.62E-09 | 3.48E+01 |
| AR | rs77796836 | T | C | 5 | 1.25E-01 | 2.01E-02 | 407746 | 5.69E-10 | 3.84E+01 |
| AR | rs77804393 | T | G | 16 | 1.14E-01 | 1.99E-02 | 407746 | 1.08E-08 | 3.27E+01 |
| AR | rs7824394 | C | A | 8 | -3.21E-02 | 5.46E-03 | 407746 | 4.15E-09 | 3.46E+01 |
| AR | rs7899004 | C | T | 10 | 3.94E-02 | 5.28E-03 | 407746 | 8.38E-14 | 5.57E+01 |
| AR | rs7925585 | G | A | 11 | -3.17E-02 | 5.43E-03 | 407746 | 5.30E-09 | 3.41E+01 |
| AR | rs7968808 | T | C | 12 | 3.69E-02 | 5.25E-03 | 407746 | 1.95E-12 | 4.95E+01 |
| AR | rs79803873 | C | T | 1 | 1.78E-01 | 2.58E-02 | 407746 | 5.24E-12 | 4.76E+01 |
| AR | rs7989041 | G | T | 13 | -3.86E-02 | 5.50E-03 | 407746 | 2.30E-12 | 4.92E+01 |
| AR | rs806338 | C | T | 13 | -3.10E-02 | 5.49E-03 | 407746 | 1.66E-08 | 3.19E+01 |
| AR | rs829636 | G | T | 2 | -3.40E-02 | 6.05E-03 | 407746 | 2.03E-08 | 3.15E+01 |
| AR | rs846977 | A | G | 6 | 5.10E-02 | 9.14E-03 | 407746 | 2.46E-08 | 3.11E+01 |
| AR | rs891058 | A | G | 2 | -6.57E-02 | 5.72E-03 | 407746 | 1.82E-30 | 1.32E+02 |
| AR | rs905670 | A | G | 6 | -5.13E-02 | 5.46E-03 | 407746 | 5.77E-21 | 8.82E+01 |
| AR | rs9268251 | A | G | 6 | -7.11E-02 | 6.16E-03 | 407746 | 8.35E-31 | 1.33E+02 |
| AR | rs9273058 | C | T | 6 | -9.22E-02 | 5.68E-03 | 407746 | 3.01E-59 | 2.63E+02 |
| AR | rs9297768 | G | A | 8 | -4.11E-02 | 5.56E-03 | 407746 | 1.49E-13 | 5.46E+01 |
| AR | rs940341 | G | A | 2 | 4.18E-02 | 6.10E-03 | 407746 | 7.64E-12 | 4.69E+01 |
| AR | rs946806 | G | A | 9 | -3.07E-02 | 5.28E-03 | 407746 | 5.98E-09 | 3.38E+01 |
| AR | rs9775039 | A | G | 9 | 7.14E-02 | 7.10E-03 | 407746 | 8.96E-24 | 1.01E+02 |
| AR | rs9808132 | C | T | 2 | -4.33E-02 | 7.54E-03 | 407746 | 9.14E-09 | 3.30E+01 |
| AR | rs9816110 | A | C | 3 | -3.66E-02 | 5.52E-03 | 407746 | 3.23E-11 | 4.40E+01 |
| AR | rs9868257 | T | C | 3 | -4.44E-02 | 5.30E-03 | 407746 | 5.24E-17 | 7.02E+01 |
| AR | rs9906189 | G | A | 17 | -3.12E-02 | 5.24E-03 | 407746 | 2.60E-09 | 3.55E+01 |
| AR | rs9978775 | G | A | 21 | -2.91E-02 | 5.28E-03 | 407746 | 3.62E-08 | 3.03E+01 |

SNPs, single nucleotide polymorphisms

IVs, qualified instrumental variables

IBD, inflammatory bowel disease

AR, allergic rhinitis

**Table2 SNPs selected as IVs for CD**

| **exposure** | **SNP** | **effect_allele** | **other_allele** | **chr** | **beta** | **se** | **samplesize** | **pval** | **F_statistic** |
| --- | --- | --- | --- | --- | --- | --- | --- | --- | --- |
| AR | rs10131567 | T | G | 14 | 3.86E-02 | 5.83E-03 | 407746 | 3.77E-11 | 4.37E+01 |
| AR | rs10244416 | T | C | 7 | -4.89E-02 | 5.24E-03 | 407746 | 9.87E-21 | 8.72E+01 |
| AR | rs10245867 | T | G | 7 | 4.55E-02 | 5.56E-03 | 407746 | 3.14E-16 | 6.67E+01 |
| AR | rs1032841 | G | A | 2 | -3.25E-02 | 5.79E-03 | 407746 | 2.03E-08 | 3.15E+01 |
| AR | rs10468514 | G | A | 17 | 3.11E-02 | 5.35E-03 | 407746 | 6.04E-09 | 3.38E+01 |
| AR | rs10485354 | G | A | 6 | -3.05E-02 | 5.27E-03 | 407746 | 7.09E-09 | 3.35E+01 |
| AR | rs10519068 | A | G | 15 | -7.12E-02 | 7.82E-03 | 407746 | 8.50E-20 | 8.29E+01 |
| AR | rs1057258 | T | C | 2 | -4.92E-02 | 6.82E-03 | 407746 | 5.47E-13 | 5.20E+01 |
| AR | rs10791824 | G | A | 11 | 5.14E-02 | 5.29E-03 | 407746 | 2.47E-22 | 9.45E+01 |
| AR | rs10795672 | A | G | 10 | 3.29E-02 | 5.32E-03 | 407746 | 6.43E-10 | 3.82E+01 |
| AR | rs10910093 | T | C | 1 | -5.76E-02 | 7.67E-03 | 407746 | 6.21E-14 | 5.63E+01 |
| AR | rs10912564 | T | C | 1 | 4.79E-02 | 5.69E-03 | 407746 | 3.83E-17 | 7.09E+01 |
| AR | rs1102737 | A | G | 1 | -5.29E-02 | 8.47E-03 | 407746 | 4.00E-10 | 3.91E+01 |
| AR | rs111267073 | T | C | 5 | -9.69E-02 | 1.70E-02 | 407746 | 1.20E-08 | 3.25E+01 |
| AR | rs112008378 | A | C | 10 | -3.27E-02 | 5.37E-03 | 407746 | 1.16E-09 | 3.70E+01 |
| AR | rs11255753 | T | G | 10 | 3.29E-02 | 5.84E-03 | 407746 | 1.85E-08 | 3.17E+01 |
| AR | rs113377887 | T | C | 13 | 4.72E-02 | 6.40E-03 | 407746 | 1.59E-13 | 5.45E+01 |
| AR | rs114695117 | A | C | 1 | -9.40E-02 | 1.61E-02 | 407746 | 5.92E-09 | 3.39E+01 |
| AR | rs1150658 | T | C | 6 | 4.48E-02 | 6.13E-03 | 407746 | 2.70E-13 | 5.34E+01 |
| AR | rs115288876 | A | G | 1 | 1.33E-01 | 1.30E-02 | 407746 | 1.45E-24 | 1.05E+02 |
| AR | rs11626205 | A | G | 14 | 3.45E-02 | 5.72E-03 | 407746 | 1.63E-09 | 3.64E+01 |
| AR | rs117137535 | A | G | 9 | 1.22E-01 | 1.80E-02 | 407746 | 9.32E-12 | 4.65E+01 |
| AR | rs11742240 | T | G | 5 | -9.17E-02 | 5.86E-03 | 407746 | 3.21E-55 | 2.45E+02 |
| AR | rs117710327 | A | C | 19 | -9.32E-02 | 1.07E-02 | 407746 | 2.47E-18 | 7.63E+01 |
| AR | rs12068304 | G | A | 1 | 4.99E-02 | 7.13E-03 | 407746 | 2.74E-12 | 4.89E+01 |
| AR | rs1214598 | A | G | 1 | -5.49E-02 | 5.37E-03 | 407746 | 1.63E-24 | 1.04E+02 |
| AR | rs12152276 | G | A | 3 | -5.65E-02 | 9.53E-03 | 407746 | 3.09E-09 | 3.51E+01 |
| AR | rs12185242 | C | A | 17 | 3.36E-02 | 5.25E-03 | 407746 | 1.68E-10 | 4.08E+01 |
| AR | rs12282231 | T | C | 11 | 6.83E-02 | 1.05E-02 | 407746 | 7.83E-11 | 4.23E+01 |
| AR | rs12365699 | A | G | 11 | -5.06E-02 | 7.04E-03 | 407746 | 7.03E-13 | 5.15E+01 |
| AR | rs12440045 | C | A | 15 | 4.48E-02 | 5.29E-03 | 407746 | 2.51E-17 | 7.17E+01 |
| AR | rs1260294 | C | T | 12 | -3.79E-02 | 6.54E-03 | 407746 | 6.83E-09 | 3.36E+01 |
| AR | rs12625547 | G | T | 20 | -4.30E-02 | 6.92E-03 | 407746 | 5.10E-10 | 3.86E+01 |
| AR | rs12948911 | T | C | 17 | -4.25E-02 | 6.68E-03 | 407746 | 1.95E-10 | 4.05E+01 |
| AR | rs16858573 | C | T | 2 | -5.54E-02 | 7.92E-03 | 407746 | 2.62E-12 | 4.90E+01 |
| AR | rs1850984 | A | G | 2 | 3.60E-02 | 6.19E-03 | 407746 | 6.01E-09 | 3.38E+01 |
| AR | rs1904522 | A | G | 4 | 6.47E-02 | 5.54E-03 | 407746 | 1.57E-31 | 1.36E+02 |
| AR | rs2070901 | T | G | 1 | 4.36E-02 | 5.89E-03 | 407746 | 1.29E-13 | 5.49E+01 |
| AR | rs2074585 | A | G | 15 | -5.31E-02 | 5.23E-03 | 407746 | 2.74E-24 | 1.03E+02 |
| AR | rs2190097 | T | C | 7 | -4.45E-02 | 6.12E-03 | 407746 | 3.73E-13 | 5.28E+01 |
| AR | rs2228145 | C | A | 1 | 3.86E-02 | 5.29E-03 | 407746 | 3.18E-13 | 5.31E+01 |
| AR | rs2235864 | A | G | 20 | 3.43E-02 | 6.03E-03 | 407746 | 1.34E-08 | 3.23E+01 |
| AR | rs2255088 | C | T | 10 | 3.37E-02 | 5.57E-03 | 407746 | 1.44E-09 | 3.66E+01 |
| AR | rs2455101 | G | A | 1 | -3.97E-02 | 6.67E-03 | 407746 | 2.53E-09 | 3.55E+01 |
| AR | rs249677 | A | C | 5 | 3.46E-02 | 5.43E-03 | 407746 | 1.88E-10 | 4.06E+01 |
| AR | rs250298 | A | G | 5 | -3.22E-02 | 5.28E-03 | 407746 | 1.08E-09 | 3.72E+01 |
| AR | rs2516518 | A | G | 6 | 8.79E-02 | 8.72E-03 | 407746 | 6.45E-24 | 1.02E+02 |
| AR | rs2723197 | A | G | 2 | -3.04E-02 | 5.27E-03 | 407746 | 7.42E-09 | 3.34E+01 |
| AR | rs2766680 | A | G | 20 | -5.51E-02 | 6.54E-03 | 407746 | 3.57E-17 | 7.10E+01 |
| AR | rs2846072 | C | T | 11 | -7.17E-02 | 1.24E-02 | 407746 | 7.64E-09 | 3.34E+01 |
| AR | rs2961920 | A | C | 5 | 4.76E-02 | 6.16E-03 | 407746 | 1.17E-14 | 5.96E+01 |
| AR | rs301806 | T | C | 1 | 3.82E-02 | 5.30E-03 | 407746 | 5.46E-13 | 5.20E+01 |
| AR | rs3024665 | T | C | 16 | 6.31E-02 | 1.10E-02 | 407746 | 8.93E-09 | 3.31E+01 |
| AR | rs3024971 | G | T | 12 | -8.31E-02 | 8.46E-03 | 407746 | 9.23E-23 | 9.64E+01 |
| AR | rs3135020 | A | C | 6 | -8.92E-02 | 8.10E-03 | 407746 | 3.16E-28 | 1.21E+02 |
| AR | rs3213567 | C | T | 12 | -3.81E-02 | 5.24E-03 | 407746 | 3.34E-13 | 5.30E+01 |
| AR | rs34119476 | G | A | 2 | -3.03E-02 | 5.31E-03 | 407746 | 1.12E-08 | 3.26E+01 |
| AR | rs34231012 | T | C | 1 | -3.45E-02 | 5.98E-03 | 407746 | 8.22E-09 | 3.32E+01 |
| AR | rs34290285 | A | G | 2 | -9.06E-02 | 5.99E-03 | 407746 | 9.16E-52 | 2.29E+02 |
| AR | rs35928002 | G | A | 19 | 3.37E-02 | 5.85E-03 | 407746 | 8.16E-09 | 3.32E+01 |
| AR | rs3730783 | G | A | 18 | -3.26E-02 | 5.33E-03 | 407746 | 9.53E-10 | 3.74E+01 |
| AR | rs3918226 | T | C | 7 | -7.06E-02 | 9.69E-03 | 407746 | 3.22E-13 | 5.31E+01 |
| AR | rs3985697 | C | T | 18 | -4.37E-02 | 6.86E-03 | 407746 | 1.92E-10 | 4.06E+01 |
| AR | rs41190 | A | C | 16 | 3.46E-02 | 6.16E-03 | 407746 | 1.90E-08 | 3.16E+01 |
| AR | rs4369774 | C | A | 18 | 3.23E-02 | 5.27E-03 | 407746 | 8.80E-10 | 3.76E+01 |
| AR | rs4379740 | C | T | 10 | -6.74E-02 | 6.77E-03 | 407746 | 2.29E-23 | 9.92E+01 |
| AR | rs45613035 | C | T | 4 | 8.67E-02 | 8.77E-03 | 407746 | 4.40E-23 | 9.79E+01 |
| AR | rs4686466 | G | A | 3 | -4.13E-02 | 6.56E-03 | 407746 | 3.10E-10 | 3.96E+01 |
| AR | rs4705869 | T | C | 5 | 4.88E-02 | 8.63E-03 | 407746 | 1.58E-08 | 3.20E+01 |
| AR | rs4769975 | A | G | 13 | -7.39E-02 | 1.34E-02 | 407746 | 3.58E-08 | 3.04E+01 |
| AR | rs4771332 | C | T | 13 | 3.13E-02 | 5.67E-03 | 407746 | 3.42E-08 | 3.05E+01 |
| AR | rs4939489 | T | C | 11 | -3.30E-02 | 5.35E-03 | 407746 | 7.31E-10 | 3.79E+01 |
| AR | rs4948563 | A | G | 10 | 3.45E-02 | 5.97E-03 | 407746 | 7.57E-09 | 3.34E+01 |
| AR | rs4995269 | G | A | 15 | 3.53E-02 | 5.54E-03 | 407746 | 1.82E-10 | 4.07E+01 |
| AR | rs5029949 | G | A | 6 | -9.37E-02 | 1.48E-02 | 407746 | 2.69E-10 | 3.99E+01 |
| AR | rs519973 | A | G | 3 | 5.09E-02 | 5.52E-03 | 407746 | 2.80E-20 | 8.51E+01 |
| AR | rs56183820 | T | C | 5 | 4.36E-02 | 6.68E-03 | 407746 | 6.37E-11 | 4.27E+01 |
| AR | rs563118370 | G | A | 11 | 1.04E-01 | 1.89E-02 | 407746 | 3.88E-08 | 3.02E+01 |
| AR | rs56854963 | T | C | 16 | -5.48E-02 | 8.59E-03 | 407746 | 1.75E-10 | 4.07E+01 |
| AR | rs5743618 | A | C | 4 | -1.29E-01 | 6.26E-03 | 407746 | 3.65E-94 | 4.24E+02 |
| AR | rs57453899 | A | G | 7 | 3.95E-02 | 6.47E-03 | 407746 | 1.02E-09 | 3.73E+01 |
| AR | rs59183580 | G | A | 11 | -4.93E-02 | 7.78E-03 | 407746 | 2.37E-10 | 4.01E+01 |
| AR | rs5997389 | A | G | 22 | 5.74E-02 | 9.60E-03 | 407746 | 2.19E-09 | 3.58E+01 |
| AR | rs60946162 | T | C | 3 | 3.49E-02 | 5.27E-03 | 407746 | 3.73E-11 | 4.37E+01 |
| AR | rs61192126 | C | T | 3 | -3.60E-02 | 5.77E-03 | 407746 | 4.49E-10 | 3.89E+01 |
| AR | rs61811906 | G | A | 1 | -2.94E-02 | 5.33E-03 | 407746 | 3.49E-08 | 3.04E+01 |
| AR | rs61813293 | T | G | 1 | -4.54E-02 | 7.44E-03 | 407746 | 1.01E-09 | 3.73E+01 |
| AR | rs61816766 | C | T | 1 | 1.48E-01 | 1.50E-02 | 407746 | 4.22E-23 | 9.80E+01 |
| AR | rs61839660 | T | C | 10 | 1.07E-01 | 8.74E-03 | 407746 | 3.36E-34 | 1.49E+02 |
| AR | rs61907710 | T | C | 11 | -4.76E-02 | 6.31E-03 | 407746 | 4.64E-14 | 5.69E+01 |
| AR | rs61938962 | T | C | 12 | 5.56E-02 | 5.51E-03 | 407746 | 5.32E-24 | 1.02E+02 |
| AR | rs62064086 | G | A | 17 | 5.13E-02 | 7.50E-03 | 407746 | 7.65E-12 | 4.69E+01 |
| AR | rs62117160 | A | G | 19 | -7.50E-02 | 1.24E-02 | 407746 | 1.70E-09 | 3.63E+01 |
| AR | rs62162287 | C | T | 2 | -3.93E-02 | 5.34E-03 | 407746 | 1.69E-13 | 5.43E+01 |
| AR | rs62328536 | A | C | 4 | -3.80E-02 | 5.48E-03 | 407746 | 3.90E-12 | 4.82E+01 |
| AR | rs62375554 | A | G | 5 | 8.34E-02 | 1.42E-02 | 407746 | 4.11E-09 | 3.46E+01 |
| AR | rs62379371 | A | G | 5 | -8.53E-02 | 1.24E-02 | 407746 | 5.10E-12 | 4.76E+01 |
| AR | rs62623446 | T | C | 12 | 6.80E-02 | 1.01E-02 | 407746 | 1.73E-11 | 4.53E+01 |
| AR | rs6594499 | A | C | 5 | -9.08E-02 | 5.24E-03 | 407746 | 3.21E-67 | 3.00E+02 |
| AR | rs6596860 | G | A | 6 | -3.33E-02 | 6.07E-03 | 407746 | 4.14E-08 | 3.01E+01 |
| AR | rs66632892 | A | G | 9 | 3.84E-02 | 5.98E-03 | 407746 | 1.36E-10 | 4.12E+01 |
| AR | rs67385163 | G | A | 12 | 3.27E-02 | 5.50E-03 | 407746 | 2.72E-09 | 3.54E+01 |
| AR | rs6938239 | A | G | 6 | 4.14E-02 | 7.53E-03 | 407746 | 3.83E-08 | 3.02E+01 |
| AR | rs709498 | G | A | 3 | 3.07E-02 | 5.26E-03 | 407746 | 5.50E-09 | 3.40E+01 |
| AR | rs71368508 | A | C | 17 | -1.38E-01 | 1.84E-02 | 407746 | 6.90E-14 | 5.61E+01 |
| AR | rs7140939 | G | A | 14 | -4.13E-02 | 5.41E-03 | 407746 | 2.34E-14 | 5.82E+01 |
| AR | rs72701682 | G | A | 9 | 3.33E-02 | 5.79E-03 | 407746 | 8.44E-09 | 3.32E+01 |
| AR | rs72702772 | A | G | 14 | -4.83E-02 | 6.55E-03 | 407746 | 1.67E-13 | 5.44E+01 |
| AR | rs73196739 | T | C | 3 | -4.75E-02 | 6.99E-03 | 407746 | 1.14E-11 | 4.61E+01 |
| AR | rs73285914 | G | A | 20 | -4.89E-02 | 8.60E-03 | 407746 | 1.31E-08 | 3.23E+01 |
| AR | rs73390208 | T | C | 6 | -8.66E-02 | 5.92E-03 | 407746 | 1.75E-48 | 2.14E+02 |
| AR | rs7410883 | C | T | 1 | -4.54E-02 | 8.31E-03 | 407746 | 4.76E-08 | 2.98E+01 |
| AR | rs7606480 | A | G | 2 | -3.16E-02 | 5.28E-03 | 407746 | 2.08E-09 | 3.59E+01 |
| AR | rs7606520 | C | A | 2 | 3.14E-02 | 5.24E-03 | 407746 | 1.95E-09 | 3.60E+01 |
| AR | rs7625643 | G | A | 3 | 3.66E-02 | 5.33E-03 | 407746 | 6.42E-12 | 4.72E+01 |
| AR | rs76282174 | T | C | 11 | 1.08E-01 | 1.60E-02 | 407746 | 1.43E-11 | 4.56E+01 |
| AR | rs7735519 | A | C | 5 | 1.23E-01 | 7.40E-03 | 407746 | 2.45E-62 | 2.78E+02 |
| AR | rs77665773 | T | C | 5 | 6.56E-02 | 1.11E-02 | 407746 | 3.88E-09 | 3.47E+01 |
| AR | rs77795650 | A | G | 6 | 7.20E-02 | 1.22E-02 | 407746 | 3.62E-09 | 3.48E+01 |
| AR | rs77796836 | T | C | 5 | 1.25E-01 | 2.01E-02 | 407746 | 5.69E-10 | 3.84E+01 |
| AR | rs77804393 | T | G | 16 | 1.14E-01 | 1.99E-02 | 407746 | 1.08E-08 | 3.27E+01 |
| AR | rs7824394 | C | A | 8 | -3.21E-02 | 5.46E-03 | 407746 | 4.15E-09 | 3.46E+01 |
| AR | rs7899004 | C | T | 10 | 3.94E-02 | 5.28E-03 | 407746 | 8.38E-14 | 5.57E+01 |
| AR | rs7925585 | G | A | 11 | -3.17E-02 | 5.43E-03 | 407746 | 5.30E-09 | 3.41E+01 |
| AR | rs7968808 | T | C | 12 | 3.69E-02 | 5.25E-03 | 407746 | 1.95E-12 | 4.95E+01 |
| AR | rs79803873 | C | T | 1 | 1.78E-01 | 2.58E-02 | 407746 | 5.24E-12 | 4.76E+01 |
| AR | rs7989041 | G | T | 13 | -3.86E-02 | 5.50E-03 | 407746 | 2.30E-12 | 4.92E+01 |
| AR | rs806338 | C | T | 13 | -3.10E-02 | 5.49E-03 | 407746 | 1.66E-08 | 3.19E+01 |
| AR | rs829636 | G | T | 2 | -3.40E-02 | 6.05E-03 | 407746 | 2.03E-08 | 3.15E+01 |
| AR | rs846977 | A | G | 6 | 5.10E-02 | 9.14E-03 | 407746 | 2.46E-08 | 3.11E+01 |
| AR | rs891058 | A | G | 2 | -6.57E-02 | 5.72E-03 | 407746 | 1.82E-30 | 1.32E+02 |
| AR | rs905670 | A | G | 6 | -5.13E-02 | 5.46E-03 | 407746 | 5.77E-21 | 8.82E+01 |
| AR | rs9268251 | A | G | 6 | -7.11E-02 | 6.16E-03 | 407746 | 8.35E-31 | 1.33E+02 |
| AR | rs9273058 | C | T | 6 | -9.22E-02 | 5.68E-03 | 407746 | 3.01E-59 | 2.63E+02 |
| AR | rs9297768 | G | A | 8 | -4.11E-02 | 5.56E-03 | 407746 | 1.49E-13 | 5.46E+01 |
| AR | rs940341 | G | A | 2 | 4.18E-02 | 6.10E-03 | 407746 | 7.64E-12 | 4.69E+01 |
| AR | rs946806 | G | A | 9 | -3.07E-02 | 5.28E-03 | 407746 | 5.98E-09 | 3.38E+01 |
| AR | rs9775039 | A | G | 9 | 7.14E-02 | 7.10E-03 | 407746 | 8.96E-24 | 1.01E+02 |
| AR | rs9808132 | C | T | 2 | -4.33E-02 | 7.54E-03 | 407746 | 9.14E-09 | 3.30E+01 |
| AR | rs9816110 | A | C | 3 | -3.66E-02 | 5.52E-03 | 407746 | 3.23E-11 | 4.40E+01 |
| AR | rs9868257 | T | C | 3 | -4.44E-02 | 5.30E-03 | 407746 | 5.24E-17 | 7.02E+01 |
| AR | rs9906189 | G | A | 17 | -3.12E-02 | 5.24E-03 | 407746 | 2.60E-09 | 3.55E+01 |
| AR | rs9978775 | G | A | 21 | -2.91E-02 | 5.28E-03 | 407746 | 3.62E-08 | 3.03E+01 |

SNPs, single nucleotide polymorphisms

IVs, qualified instrumental variables

CD, Crohn's disease

AR, allergic rhinitis

**Table3 SNPs selected as IVs for UC**

| **exposure** | **SNP** | **effect_allele** | **other_allele** | **chr** | **beta** | **se** | **samplesize** | **pval** | **F_statistic** |
| --- | --- | --- | --- | --- | --- | --- | --- | --- | --- |
| AR | rs10131567 | T | G | 14 | 3.86E-02 | 5.83E-03 | 407746 | 3.77E-11 | 4.37E+01 |
| AR | rs10244416 | T | C | 7 | -4.89E-02 | 5.24E-03 | 407746 | 9.87E-21 | 8.72E+01 |
| AR | rs10245867 | T | G | 7 | 4.55E-02 | 5.56E-03 | 407746 | 3.14E-16 | 6.67E+01 |
| AR | rs1032841 | G | A | 2 | -3.25E-02 | 5.79E-03 | 407746 | 2.03E-08 | 3.15E+01 |
| AR | rs10468514 | G | A | 17 | 3.11E-02 | 5.35E-03 | 407746 | 6.04E-09 | 3.38E+01 |
| AR | rs10485354 | G | A | 6 | -3.05E-02 | 5.27E-03 | 407746 | 7.09E-09 | 3.35E+01 |
| AR | rs10519068 | A | G | 15 | -7.12E-02 | 7.82E-03 | 407746 | 8.50E-20 | 8.29E+01 |
| AR | rs1057258 | T | C | 2 | -4.92E-02 | 6.82E-03 | 407746 | 5.47E-13 | 5.20E+01 |
| AR | rs10791824 | G | A | 11 | 5.14E-02 | 5.29E-03 | 407746 | 2.47E-22 | 9.45E+01 |
| AR | rs10795672 | A | G | 10 | 3.29E-02 | 5.32E-03 | 407746 | 6.43E-10 | 3.82E+01 |
| AR | rs10910093 | T | C | 1 | -5.76E-02 | 7.67E-03 | 407746 | 6.21E-14 | 5.63E+01 |
| AR | rs10912564 | T | C | 1 | 4.79E-02 | 5.69E-03 | 407746 | 3.83E-17 | 7.09E+01 |
| AR | rs1102737 | A | G | 1 | -5.29E-02 | 8.47E-03 | 407746 | 4.00E-10 | 3.91E+01 |
| AR | rs111267073 | T | C | 5 | -9.69E-02 | 1.70E-02 | 407746 | 1.20E-08 | 3.25E+01 |
| AR | rs112008378 | A | C | 10 | -3.27E-02 | 5.37E-03 | 407746 | 1.16E-09 | 3.70E+01 |
| AR | rs11255753 | T | G | 10 | 3.29E-02 | 5.84E-03 | 407746 | 1.85E-08 | 3.17E+01 |
| AR | rs113377887 | T | C | 13 | 4.72E-02 | 6.40E-03 | 407746 | 1.59E-13 | 5.45E+01 |
| AR | rs114695117 | A | C | 1 | -9.40E-02 | 1.61E-02 | 407746 | 5.92E-09 | 3.39E+01 |
| AR | rs1150658 | T | C | 6 | 4.48E-02 | 6.13E-03 | 407746 | 2.70E-13 | 5.34E+01 |
| AR | rs115288876 | A | G | 1 | 1.33E-01 | 1.30E-02 | 407746 | 1.45E-24 | 1.05E+02 |
| AR | rs11626205 | A | G | 14 | 3.45E-02 | 5.72E-03 | 407746 | 1.63E-09 | 3.64E+01 |
| AR | rs117137535 | A | G | 9 | 1.22E-01 | 1.80E-02 | 407746 | 9.32E-12 | 4.65E+01 |
| AR | rs11742240 | T | G | 5 | -9.17E-02 | 5.86E-03 | 407746 | 3.21E-55 | 2.45E+02 |
| AR | rs117710327 | A | C | 19 | -9.32E-02 | 1.07E-02 | 407746 | 2.47E-18 | 7.63E+01 |
| AR | rs12068304 | G | A | 1 | 4.99E-02 | 7.13E-03 | 407746 | 2.74E-12 | 4.89E+01 |
| AR | rs1214598 | A | G | 1 | -5.49E-02 | 5.37E-03 | 407746 | 1.63E-24 | 1.04E+02 |
| AR | rs12152276 | G | A | 3 | -5.65E-02 | 9.53E-03 | 407746 | 3.09E-09 | 3.51E+01 |
| AR | rs12185242 | C | A | 17 | 3.36E-02 | 5.25E-03 | 407746 | 1.68E-10 | 4.08E+01 |
| AR | rs12282231 | T | C | 11 | 6.83E-02 | 1.05E-02 | 407746 | 7.83E-11 | 4.23E+01 |
| AR | rs12365699 | A | G | 11 | -5.06E-02 | 7.04E-03 | 407746 | 7.03E-13 | 5.15E+01 |
| AR | rs12440045 | C | A | 15 | 4.48E-02 | 5.29E-03 | 407746 | 2.51E-17 | 7.17E+01 |
| AR | rs1260294 | C | T | 12 | -3.79E-02 | 6.54E-03 | 407746 | 6.83E-09 | 3.36E+01 |
| AR | rs12625547 | G | T | 20 | -4.30E-02 | 6.92E-03 | 407746 | 5.10E-10 | 3.86E+01 |
| AR | rs12948911 | T | C | 17 | -4.25E-02 | 6.68E-03 | 407746 | 1.95E-10 | 4.05E+01 |
| AR | rs16858573 | C | T | 2 | -5.54E-02 | 7.92E-03 | 407746 | 2.62E-12 | 4.90E+01 |
| AR | rs1850984 | A | G | 2 | 3.60E-02 | 6.19E-03 | 407746 | 6.01E-09 | 3.38E+01 |
| AR | rs1904522 | A | G | 4 | 6.47E-02 | 5.54E-03 | 407746 | 1.57E-31 | 1.36E+02 |
| AR | rs2070901 | T | G | 1 | 4.36E-02 | 5.89E-03 | 407746 | 1.29E-13 | 5.49E+01 |
| AR | rs2074585 | A | G | 15 | -5.31E-02 | 5.23E-03 | 407746 | 2.74E-24 | 1.03E+02 |
| AR | rs2190097 | T | C | 7 | -4.45E-02 | 6.12E-03 | 407746 | 3.73E-13 | 5.28E+01 |
| AR | rs2228145 | C | A | 1 | 3.86E-02 | 5.29E-03 | 407746 | 3.18E-13 | 5.31E+01 |
| AR | rs2235864 | A | G | 20 | 3.43E-02 | 6.03E-03 | 407746 | 1.34E-08 | 3.23E+01 |
| AR | rs2255088 | C | T | 10 | 3.37E-02 | 5.57E-03 | 407746 | 1.44E-09 | 3.66E+01 |
| AR | rs2455101 | G | A | 1 | -3.97E-02 | 6.67E-03 | 407746 | 2.53E-09 | 3.55E+01 |
| AR | rs249677 | A | C | 5 | 3.46E-02 | 5.43E-03 | 407746 | 1.88E-10 | 4.06E+01 |
| AR | rs250298 | A | G | 5 | -3.22E-02 | 5.28E-03 | 407746 | 1.08E-09 | 3.72E+01 |
| AR | rs2516518 | A | G | 6 | 8.79E-02 | 8.72E-03 | 407746 | 6.45E-24 | 1.02E+02 |
| AR | rs2723197 | A | G | 2 | -3.04E-02 | 5.27E-03 | 407746 | 7.42E-09 | 3.34E+01 |
| AR | rs2766680 | A | G | 20 | -5.51E-02 | 6.54E-03 | 407746 | 3.57E-17 | 7.10E+01 |
| AR | rs2846072 | C | T | 11 | -7.17E-02 | 1.24E-02 | 407746 | 7.64E-09 | 3.34E+01 |
| AR | rs2961920 | A | C | 5 | 4.76E-02 | 6.16E-03 | 407746 | 1.17E-14 | 5.96E+01 |
| AR | rs301806 | T | C | 1 | 3.82E-02 | 5.30E-03 | 407746 | 5.46E-13 | 5.20E+01 |
| AR | rs3024665 | T | C | 16 | 6.31E-02 | 1.10E-02 | 407746 | 8.93E-09 | 3.31E+01 |
| AR | rs3024971 | G | T | 12 | -8.31E-02 | 8.46E-03 | 407746 | 9.23E-23 | 9.64E+01 |
| AR | rs3135020 | A | C | 6 | -8.92E-02 | 8.10E-03 | 407746 | 3.16E-28 | 1.21E+02 |
| AR | rs3213567 | C | T | 12 | -3.81E-02 | 5.24E-03 | 407746 | 3.34E-13 | 5.30E+01 |
| AR | rs34119476 | G | A | 2 | -3.03E-02 | 5.31E-03 | 407746 | 1.12E-08 | 3.26E+01 |
| AR | rs34231012 | T | C | 1 | -3.45E-02 | 5.98E-03 | 407746 | 8.22E-09 | 3.32E+01 |
| AR | rs35928002 | G | A | 19 | 3.37E-02 | 5.85E-03 | 407746 | 8.16E-09 | 3.32E+01 |
| AR | rs3730783 | G | A | 18 | -3.26E-02 | 5.33E-03 | 407746 | 9.53E-10 | 3.74E+01 |
| AR | rs3918226 | T | C | 7 | -7.06E-02 | 9.69E-03 | 407746 | 3.22E-13 | 5.31E+01 |
| AR | rs3985697 | C | T | 18 | -4.37E-02 | 6.86E-03 | 407746 | 1.92E-10 | 4.06E+01 |
| AR | rs41190 | A | C | 16 | 3.46E-02 | 6.16E-03 | 407746 | 1.90E-08 | 3.16E+01 |
| AR | rs4369774 | C | A | 18 | 3.23E-02 | 5.27E-03 | 407746 | 8.80E-10 | 3.76E+01 |
| AR | rs4379740 | C | T | 10 | -6.74E-02 | 6.77E-03 | 407746 | 2.29E-23 | 9.92E+01 |
| AR | rs45613035 | C | T | 4 | 8.67E-02 | 8.77E-03 | 407746 | 4.40E-23 | 9.79E+01 |
| AR | rs4686466 | G | A | 3 | -4.13E-02 | 6.56E-03 | 407746 | 3.10E-10 | 3.96E+01 |
| AR | rs4705869 | T | C | 5 | 4.88E-02 | 8.63E-03 | 407746 | 1.58E-08 | 3.20E+01 |
| AR | rs4769975 | A | G | 13 | -7.39E-02 | 1.34E-02 | 407746 | 3.58E-08 | 3.04E+01 |
| AR | rs4771332 | C | T | 13 | 3.13E-02 | 5.67E-03 | 407746 | 3.42E-08 | 3.05E+01 |
| AR | rs4939489 | T | C | 11 | -3.30E-02 | 5.35E-03 | 407746 | 7.31E-10 | 3.79E+01 |
| AR | rs4948563 | A | G | 10 | 3.45E-02 | 5.97E-03 | 407746 | 7.57E-09 | 3.34E+01 |
| AR | rs4995269 | G | A | 15 | 3.53E-02 | 5.54E-03 | 407746 | 1.82E-10 | 4.07E+01 |
| AR | rs5029949 | G | A | 6 | -9.37E-02 | 1.48E-02 | 407746 | 2.69E-10 | 3.99E+01 |
| AR | rs519973 | A | G | 3 | 5.09E-02 | 5.52E-03 | 407746 | 2.80E-20 | 8.51E+01 |
| AR | rs56183820 | T | C | 5 | 4.36E-02 | 6.68E-03 | 407746 | 6.37E-11 | 4.27E+01 |
| AR | rs563118370 | G | A | 11 | 1.04E-01 | 1.89E-02 | 407746 | 3.88E-08 | 3.02E+01 |
| AR | rs56854963 | T | C | 16 | -5.48E-02 | 8.59E-03 | 407746 | 1.75E-10 | 4.07E+01 |
| AR | rs5743618 | A | C | 4 | -1.29E-01 | 6.26E-03 | 407746 | 3.65E-94 | 4.24E+02 |
| AR | rs57453899 | A | G | 7 | 3.95E-02 | 6.47E-03 | 407746 | 1.02E-09 | 3.73E+01 |
| AR | rs59183580 | G | A | 11 | -4.93E-02 | 7.78E-03 | 407746 | 2.37E-10 | 4.01E+01 |
| AR | rs5997389 | A | G | 22 | 5.74E-02 | 9.60E-03 | 407746 | 2.19E-09 | 3.58E+01 |
| AR | rs60946162 | T | C | 3 | 3.49E-02 | 5.27E-03 | 407746 | 3.73E-11 | 4.37E+01 |
| AR | rs61192126 | C | T | 3 | -3.60E-02 | 5.77E-03 | 407746 | 4.49E-10 | 3.89E+01 |
| AR | rs61811906 | G | A | 1 | -2.94E-02 | 5.33E-03 | 407746 | 3.49E-08 | 3.04E+01 |
| AR | rs61813293 | T | G | 1 | -4.54E-02 | 7.44E-03 | 407746 | 1.01E-09 | 3.73E+01 |
| AR | rs61816766 | C | T | 1 | 1.48E-01 | 1.50E-02 | 407746 | 4.22E-23 | 9.80E+01 |
| AR | rs61839660 | T | C | 10 | 1.07E-01 | 8.74E-03 | 407746 | 3.36E-34 | 1.49E+02 |
| AR | rs61907710 | T | C | 11 | -4.76E-02 | 6.31E-03 | 407746 | 4.64E-14 | 5.69E+01 |
| AR | rs61938962 | T | C | 12 | 5.56E-02 | 5.51E-03 | 407746 | 5.32E-24 | 1.02E+02 |
| AR | rs62064086 | G | A | 17 | 5.13E-02 | 7.50E-03 | 407746 | 7.65E-12 | 4.69E+01 |
| AR | rs62117160 | A | G | 19 | -7.50E-02 | 1.24E-02 | 407746 | 1.70E-09 | 3.63E+01 |
| AR | rs62162287 | C | T | 2 | -3.93E-02 | 5.34E-03 | 407746 | 1.69E-13 | 5.43E+01 |
| AR | rs62328536 | A | C | 4 | -3.80E-02 | 5.48E-03 | 407746 | 3.90E-12 | 4.82E+01 |
| AR | rs62375554 | A | G | 5 | 8.34E-02 | 1.42E-02 | 407746 | 4.11E-09 | 3.46E+01 |
| AR | rs62379371 | A | G | 5 | -8.53E-02 | 1.24E-02 | 407746 | 5.10E-12 | 4.76E+01 |
| AR | rs62623446 | T | C | 12 | 6.80E-02 | 1.01E-02 | 407746 | 1.73E-11 | 4.53E+01 |
| AR | rs6594499 | A | C | 5 | -9.08E-02 | 5.24E-03 | 407746 | 3.21E-67 | 3.00E+02 |
| AR | rs6596860 | G | A | 6 | -3.33E-02 | 6.07E-03 | 407746 | 4.14E-08 | 3.01E+01 |
| AR | rs66632892 | A | G | 9 | 3.84E-02 | 5.98E-03 | 407746 | 1.36E-10 | 4.12E+01 |
| AR | rs67385163 | G | A | 12 | 3.27E-02 | 5.50E-03 | 407746 | 2.72E-09 | 3.54E+01 |
| AR | rs6938239 | A | G | 6 | 4.14E-02 | 7.53E-03 | 407746 | 3.83E-08 | 3.02E+01 |
| AR | rs709498 | G | A | 3 | 3.07E-02 | 5.26E-03 | 407746 | 5.50E-09 | 3.40E+01 |
| AR | rs71368508 | A | C | 17 | -1.38E-01 | 1.84E-02 | 407746 | 6.90E-14 | 5.61E+01 |
| AR | rs7140939 | G | A | 14 | -4.13E-02 | 5.41E-03 | 407746 | 2.34E-14 | 5.82E+01 |
| AR | rs72701682 | G | A | 9 | 3.33E-02 | 5.79E-03 | 407746 | 8.44E-09 | 3.32E+01 |
| AR | rs72702772 | A | G | 14 | -4.83E-02 | 6.55E-03 | 407746 | 1.67E-13 | 5.44E+01 |
| AR | rs73196739 | T | C | 3 | -4.75E-02 | 6.99E-03 | 407746 | 1.14E-11 | 4.61E+01 |
| AR | rs73285914 | G | A | 20 | -4.89E-02 | 8.60E-03 | 407746 | 1.31E-08 | 3.23E+01 |
| AR | rs73390208 | T | C | 6 | -8.66E-02 | 5.92E-03 | 407746 | 1.75E-48 | 2.14E+02 |
| AR | rs7410883 | C | T | 1 | -4.54E-02 | 8.31E-03 | 407746 | 4.76E-08 | 2.98E+01 |
| AR | rs7606480 | A | G | 2 | -3.16E-02 | 5.28E-03 | 407746 | 2.08E-09 | 3.59E+01 |
| AR | rs7606520 | C | A | 2 | 3.14E-02 | 5.24E-03 | 407746 | 1.95E-09 | 3.60E+01 |
| AR | rs7625643 | G | A | 3 | 3.66E-02 | 5.33E-03 | 407746 | 6.42E-12 | 4.72E+01 |
| AR | rs76282174 | T | C | 11 | 1.08E-01 | 1.60E-02 | 407746 | 1.43E-11 | 4.56E+01 |
| AR | rs7735519 | A | C | 5 | 1.23E-01 | 7.40E-03 | 407746 | 2.45E-62 | 2.78E+02 |
| AR | rs77665773 | T | C | 5 | 6.56E-02 | 1.11E-02 | 407746 | 3.88E-09 | 3.47E+01 |
| AR | rs77795650 | A | G | 6 | 7.20E-02 | 1.22E-02 | 407746 | 3.62E-09 | 3.48E+01 |
| AR | rs77796836 | T | C | 5 | 1.25E-01 | 2.01E-02 | 407746 | 5.69E-10 | 3.84E+01 |
| AR | rs77804393 | T | G | 16 | 1.14E-01 | 1.99E-02 | 407746 | 1.08E-08 | 3.27E+01 |
| AR | rs7824394 | C | A | 8 | -3.21E-02 | 5.46E-03 | 407746 | 4.15E-09 | 3.46E+01 |
| AR | rs7899004 | C | T | 10 | 3.94E-02 | 5.28E-03 | 407746 | 8.38E-14 | 5.57E+01 |
| AR | rs7925585 | G | A | 11 | -3.17E-02 | 5.43E-03 | 407746 | 5.30E-09 | 3.41E+01 |
| AR | rs7968808 | T | C | 12 | 3.69E-02 | 5.25E-03 | 407746 | 1.95E-12 | 4.95E+01 |
| AR | rs79803873 | C | T | 1 | 1.78E-01 | 2.58E-02 | 407746 | 5.24E-12 | 4.76E+01 |
| AR | rs7989041 | G | T | 13 | -3.86E-02 | 5.50E-03 | 407746 | 2.30E-12 | 4.92E+01 |
| AR | rs806338 | C | T | 13 | -3.10E-02 | 5.49E-03 | 407746 | 1.66E-08 | 3.19E+01 |
| AR | rs829636 | G | T | 2 | -3.40E-02 | 6.05E-03 | 407746 | 2.03E-08 | 3.15E+01 |
| AR | rs846977 | A | G | 6 | 5.10E-02 | 9.14E-03 | 407746 | 2.46E-08 | 3.11E+01 |
| AR | rs891058 | A | G | 2 | -6.57E-02 | 5.72E-03 | 407746 | 1.82E-30 | 1.32E+02 |
| AR | rs905670 | A | G | 6 | -5.13E-02 | 5.46E-03 | 407746 | 5.77E-21 | 8.82E+01 |
| AR | rs9268251 | A | G | 6 | -7.11E-02 | 6.16E-03 | 407746 | 8.35E-31 | 1.33E+02 |
| AR | rs9297768 | G | A | 8 | -4.11E-02 | 5.56E-03 | 407746 | 1.49E-13 | 5.46E+01 |
| AR | rs940341 | G | A | 2 | 4.18E-02 | 6.10E-03 | 407746 | 7.64E-12 | 4.69E+01 |
| AR | rs946806 | G | A | 9 | -3.07E-02 | 5.28E-03 | 407746 | 5.98E-09 | 3.38E+01 |
| AR | rs9775039 | A | G | 9 | 7.14E-02 | 7.10E-03 | 407746 | 8.96E-24 | 1.01E+02 |
| AR | rs9808132 | C | T | 2 | -4.33E-02 | 7.54E-03 | 407746 | 9.14E-09 | 3.30E+01 |
| AR | rs9816110 | A | C | 3 | -3.66E-02 | 5.52E-03 | 407746 | 3.23E-11 | 4.40E+01 |
| AR | rs9868257 | T | C | 3 | -4.44E-02 | 5.30E-03 | 407746 | 5.24E-17 | 7.02E+01 |
| AR | rs9906189 | G | A | 17 | -3.12E-02 | 5.24E-03 | 407746 | 2.60E-09 | 3.55E+01 |
| AR | rs9978775 | G | A | 21 | -2.91E-02 | 5.28E-03 | 407746 | 3.62E-08 | 3.03E+01 |

SNPs, single nucleotide polymorphisms

IVs, qualified instrumental variables

UC, ulcerative colitis

AR, allergic rhinitis

**Table4 SNPs selected as IVs for IBD**

| **exposure** | **SNP** | **effect_allele** | **other_allele** | **chr** | **beta** | **se** | **samplesize** | **pval** | **F_statistic** |
| --- | --- | --- | --- | --- | --- | --- | --- | --- | --- |
| Asthma | rs10456939 | A | G | 6 | -5.09E-03 | 8.99E-04 | 336782 | 1.53E-08 | 3.20E+01 |
| Asthma | rs10795633 | C | T | 10 | -5.33E-03 | 8.01E-04 | 336782 | 2.85E-11 | 4.43E+01 |
| Asthma | rs10836537 | A | G | 11 | -4.53E-03 | 8.19E-04 | 336782 | 3.12E-08 | 3.06E+01 |
| Asthma | rs11071559 | T | C | 15 | -9.58E-03 | 1.16E-03 | 336782 | 1.69E-16 | 6.79E+01 |
| Asthma | rs11178649 | T | G | 12 | -5.11E-03 | 7.93E-04 | 336782 | 1.22E-10 | 4.14E+01 |
| Asthma | rs11255753 | T | G | 10 | 5.80E-03 | 8.69E-04 | 336782 | 2.53E-11 | 4.45E+01 |
| Asthma | rs1136778 | G | A | 6 | 1.82E-02 | 1.50E-03 | 336782 | 7.96E-34 | 1.47E+02 |
| Asthma | rs115018313 | C | T | 6 | 1.60E-02 | 2.55E-03 | 336782 | 4.06E-10 | 3.91E+01 |
| Asthma | rs116548543 | G | T | 6 | -1.82E-02 | 3.07E-03 | 336782 | 3.17E-09 | 3.51E+01 |
| Asthma | rs117710327 | A | C | 19 | -1.52E-02 | 1.59E-03 | 336782 | 8.31E-22 | 9.21E+01 |
| Asthma | rs12123821 | T | C | 1 | 2.02E-02 | 1.82E-03 | 336782 | 8.17E-29 | 1.24E+02 |
| Asthma | rs12356840 | A | G | 10 | 1.02E-02 | 1.59E-03 | 336782 | 1.37E-10 | 4.12E+01 |
| Asthma | rs12365699 | A | G | 11 | -5.74E-03 | 1.05E-03 | 336782 | 4.36E-08 | 3.00E+01 |
| Asthma | rs12700215 | A | G | 7 | -5.25E-03 | 8.13E-04 | 336782 | 1.09E-10 | 4.17E+01 |
| Asthma | rs12788104 | G | A | 11 | 4.63E-03 | 8.41E-04 | 336782 | 3.59E-08 | 3.04E+01 |
| Asthma | rs12952581 | A | G | 17 | 6.58E-03 | 8.10E-04 | 336782 | 4.84E-16 | 6.59E+01 |
| Asthma | rs12964116 | G | A | 18 | 1.17E-02 | 2.10E-03 | 336782 | 2.61E-08 | 3.10E+01 |
| Asthma | rs13208164 | A | G | 6 | 5.22E-03 | 8.81E-04 | 336782 | 3.26E-09 | 3.50E+01 |
| Asthma | rs1321859 | T | C | 6 | -8.36E-03 | 8.20E-04 | 336782 | 2.00E-24 | 1.04E+02 |
| Asthma | rs13355228 | T | C | 5 | 6.86E-03 | 1.15E-03 | 336782 | 2.34E-09 | 3.57E+01 |
| Asthma | rs146003472 | C | T | 6 | 2.09E-02 | 3.05E-03 | 336782 | 8.77E-12 | 4.66E+01 |
| Asthma | rs148586558 | T | C | 15 | 1.14E-02 | 1.80E-03 | 336782 | 2.36E-10 | 4.01E+01 |
| Asthma | rs150289743 | A | G | 19 | 1.36E-02 | 2.45E-03 | 336782 | 2.75E-08 | 3.09E+01 |
| Asthma | rs150971244 | T | G | 7 | 5.13E-03 | 8.95E-04 | 336782 | 1.01E-08 | 3.28E+01 |
| Asthma | rs1684466 | A | G | 3 | -5.19E-03 | 8.37E-04 | 336782 | 5.59E-10 | 3.85E+01 |
| Asthma | rs174535 | C | T | 11 | -5.12E-03 | 8.16E-04 | 336782 | 3.35E-10 | 3.95E+01 |
| Asthma | rs17454584 | G | A | 4 | 7.27E-03 | 9.41E-04 | 336782 | 1.07E-14 | 5.98E+01 |
| Asthma | rs1789358 | A | G | 11 | 5.50E-03 | 8.59E-04 | 336782 | 1.49E-10 | 4.10E+01 |
| Asthma | rs1837253 | C | T | 5 | 1.14E-02 | 8.87E-04 | 336782 | 4.20E-38 | 1.67E+02 |
| Asthma | rs2025758 | C | T | 10 | -6.05E-03 | 7.83E-04 | 336782 | 1.14E-14 | 5.96E+01 |
| Asthma | rs2197415 | G | T | 10 | 1.02E-02 | 7.89E-04 | 336782 | 5.39E-38 | 1.66E+02 |
| Asthma | rs2296618 | G | A | 1 | -6.63E-03 | 1.15E-03 | 336782 | 7.81E-09 | 3.33E+01 |
| Asthma | rs2299012 | C | A | 5 | 1.04E-02 | 9.88E-04 | 336782 | 4.50E-26 | 1.12E+02 |
| Asthma | rs2338819 | T | C | 5 | -5.24E-03 | 8.59E-04 | 336782 | 1.08E-09 | 3.72E+01 |
| Asthma | rs3024664 | C | T | 16 | 1.25E-02 | 1.64E-03 | 336782 | 2.62E-14 | 5.80E+01 |
| Asthma | rs3024971 | G | T | 12 | -1.31E-02 | 1.26E-03 | 336782 | 2.81E-25 | 1.08E+02 |
| Asthma | rs34173062 | A | G | 8 | 9.71E-03 | 1.60E-03 | 336782 | 1.17E-09 | 3.70E+01 |
| Asthma | rs34290285 | A | G | 2 | -1.10E-02 | 8.92E-04 | 336782 | 9.33E-35 | 1.51E+02 |
| Asthma | rs35570272 | T | G | 3 | 5.59E-03 | 8.00E-04 | 336782 | 2.83E-12 | 4.88E+01 |
| Asthma | rs3781094 | C | A | 10 | -4.74E-03 | 8.28E-04 | 336782 | 1.07E-08 | 3.27E+01 |
| Asthma | rs3784099 | A | G | 14 | 6.13E-03 | 8.67E-04 | 336782 | 1.54E-12 | 5.00E+01 |
| Asthma | rs3806933 | T | C | 5 | -7.06E-03 | 7.86E-04 | 336782 | 2.50E-19 | 8.08E+01 |
| Asthma | rs3807947 | G | T | 7 | -5.03E-03 | 7.95E-04 | 336782 | 2.47E-10 | 4.01E+01 |
| Asthma | rs3825568 | T | C | 14 | -4.70E-03 | 7.81E-04 | 336782 | 1.75E-09 | 3.62E+01 |
| Asthma | rs3856439 | T | C | 2 | -6.04E-03 | 8.23E-04 | 336782 | 2.09E-13 | 5.39E+01 |
| Asthma | rs4073332 | A | G | 16 | 4.87E-03 | 8.19E-04 | 336782 | 2.79E-09 | 3.53E+01 |
| Asthma | rs41283642 | T | C | 9 | -1.34E-02 | 2.15E-03 | 336782 | 5.32E-10 | 3.86E+01 |
| Asthma | rs41284471 | A | G | 10 | 5.74E-03 | 9.71E-04 | 336782 | 3.52E-09 | 3.49E+01 |
| Asthma | rs429916 | A | C | 6 | 8.30E-03 | 1.37E-03 | 336782 | 1.36E-09 | 3.67E+01 |
| Asthma | rs4480384 | G | A | 1 | 4.81E-03 | 8.12E-04 | 336782 | 3.15E-09 | 3.51E+01 |
| Asthma | rs4594881 | T | G | 5 | -4.82E-03 | 8.20E-04 | 336782 | 4.18E-09 | 3.45E+01 |
| Asthma | rs4739738 | A | G | 8 | -7.35E-03 | 8.12E-04 | 336782 | 1.31E-19 | 8.21E+01 |
| Asthma | rs479844 | G | A | 11 | 4.92E-03 | 7.82E-04 | 336782 | 3.05E-10 | 3.96E+01 |
| Asthma | rs4842921 | A | G | 15 | -4.49E-03 | 7.98E-04 | 336782 | 1.79E-08 | 3.17E+01 |
| Asthma | rs55661102 | G | A | 3 | -5.99E-03 | 1.04E-03 | 336782 | 8.49E-09 | 3.32E+01 |
| Asthma | rs58029167 | G | A | 9 | 6.55E-03 | 8.74E-04 | 336782 | 6.58E-14 | 5.62E+01 |
| Asthma | rs61816766 | C | T | 1 | 1.65E-02 | 2.23E-03 | 336782 | 1.35E-13 | 5.48E+01 |
| Asthma | rs61946384 | A | C | 12 | -4.48E-03 | 8.18E-04 | 336782 | 4.30E-08 | 3.00E+01 |
| Asthma | rs6729966 | G | A | 2 | -4.49E-03 | 8.01E-04 | 336782 | 2.16E-08 | 3.13E+01 |
| Asthma | rs7030351 | C | T | 9 | 4.69E-03 | 8.53E-04 | 336782 | 3.75E-08 | 3.03E+01 |
| Asthma | rs7134784 | C | T | 12 | 6.52E-03 | 1.09E-03 | 336782 | 2.36E-09 | 3.56E+01 |
| Asthma | rs7166081 | A | G | 15 | 6.79E-03 | 9.22E-04 | 336782 | 1.73E-13 | 5.43E+01 |
| Asthma | rs72799426 | G | A | 5 | 1.03E-02 | 1.56E-03 | 336782 | 3.46E-11 | 4.39E+01 |
| Asthma | rs75125788 | T | C | 10 | -8.66E-03 | 1.43E-03 | 336782 | 1.46E-09 | 3.66E+01 |
| Asthma | rs75332898 | A | G | 12 | 9.01E-03 | 1.61E-03 | 336782 | 2.23E-08 | 3.13E+01 |
| Asthma | rs76282174 | T | C | 11 | 1.80E-02 | 2.38E-03 | 336782 | 3.54E-14 | 5.74E+01 |
| Asthma | rs7735519 | A | C | 5 | 9.77E-03 | 1.10E-03 | 336782 | 7.08E-19 | 7.88E+01 |
| Asthma | rs8103278 | A | G | 19 | -5.08E-03 | 8.18E-04 | 336782 | 5.02E-10 | 3.87E+01 |
| Asthma | rs840012 | T | C | 1 | -5.94E-03 | 7.95E-04 | 336782 | 8.03E-14 | 5.58E+01 |
| Asthma | rs903361 | A | G | 1 | 5.02E-03 | 8.23E-04 | 336782 | 1.06E-09 | 3.72E+01 |
| Asthma | rs912131 | G | A | 13 | 7.22E-03 | 8.51E-04 | 336782 | 2.19E-17 | 7.20E+01 |
| Asthma | rs917115 | C | T | 7 | 6.72E-03 | 9.58E-04 | 336782 | 2.25E-12 | 4.93E+01 |
| Asthma | rs9344188 | T | C | 6 | 4.61E-03 | 7.83E-04 | 336782 | 3.91E-09 | 3.47E+01 |
| Asthma | rs9368851 | G | A | 6 | 6.43E-03 | 1.05E-03 | 336782 | 1.04E-09 | 3.72E+01 |
| Asthma | rs947591 | A | C | 10 | -4.46E-03 | 7.92E-04 | 336782 | 1.78E-08 | 3.17E+01 |
| Asthma | rs992969 | G | A | 9 | -1.42E-02 | 8.97E-04 | 336782 | 1.12E-56 | 2.52E+02 |

SNPs, single nucleotide polymorphisms

IVs, qualified instrumental variables

IBD, inflammatory bowel disease

**Table5 SNPs selected as IVs for CD**

| **exposure** | **SNP** | **effect_allele** | **other_allele** | **chr** | **beta** | **se** | **samplesize** | **pval** | **F_statistic** |
| --- | --- | --- | --- | --- | --- | --- | --- | --- | --- |
| Asthma | rs10456939 | A | G | 6 | -5.09E-03 | 8.99E-04 | 336782 | 1.53E-08 | 3.20E+01 |
| Asthma | rs10795633 | C | T | 10 | -5.33E-03 | 8.01E-04 | 336782 | 2.85E-11 | 4.43E+01 |
| Asthma | rs10836537 | A | G | 11 | -4.53E-03 | 8.19E-04 | 336782 | 3.12E-08 | 3.06E+01 |
| Asthma | rs11071559 | T | C | 15 | -9.58E-03 | 1.16E-03 | 336782 | 1.69E-16 | 6.79E+01 |
| Asthma | rs11178649 | T | G | 12 | -5.11E-03 | 7.93E-04 | 336782 | 1.22E-10 | 4.14E+01 |
| Asthma | rs11255753 | T | G | 10 | 5.80E-03 | 8.69E-04 | 336782 | 2.53E-11 | 4.45E+01 |
| Asthma | rs1136778 | G | A | 6 | 1.82E-02 | 1.50E-03 | 336782 | 7.96E-34 | 1.47E+02 |
| Asthma | rs115018313 | C | T | 6 | 1.60E-02 | 2.55E-03 | 336782 | 4.06E-10 | 3.91E+01 |
| Asthma | rs116548543 | G | T | 6 | -1.82E-02 | 3.07E-03 | 336782 | 3.17E-09 | 3.51E+01 |
| Asthma | rs117710327 | A | C | 19 | -1.52E-02 | 1.59E-03 | 336782 | 8.31E-22 | 9.21E+01 |
| Asthma | rs12123821 | T | C | 1 | 2.02E-02 | 1.82E-03 | 336782 | 8.17E-29 | 1.24E+02 |
| Asthma | rs12356840 | A | G | 10 | 1.02E-02 | 1.59E-03 | 336782 | 1.37E-10 | 4.12E+01 |
| Asthma | rs12365699 | A | G | 11 | -5.74E-03 | 1.05E-03 | 336782 | 4.36E-08 | 3.00E+01 |
| Asthma | rs12700215 | A | G | 7 | -5.25E-03 | 8.13E-04 | 336782 | 1.09E-10 | 4.17E+01 |
| Asthma | rs12788104 | G | A | 11 | 4.63E-03 | 8.41E-04 | 336782 | 3.59E-08 | 3.04E+01 |
| Asthma | rs12952581 | A | G | 17 | 6.58E-03 | 8.10E-04 | 336782 | 4.84E-16 | 6.59E+01 |
| Asthma | rs12964116 | G | A | 18 | 1.17E-02 | 2.10E-03 | 336782 | 2.61E-08 | 3.10E+01 |
| Asthma | rs13208164 | A | G | 6 | 5.22E-03 | 8.81E-04 | 336782 | 3.26E-09 | 3.50E+01 |
| Asthma | rs1321859 | T | C | 6 | -8.36E-03 | 8.20E-04 | 336782 | 2.00E-24 | 1.04E+02 |
| Asthma | rs13355228 | T | C | 5 | 6.86E-03 | 1.15E-03 | 336782 | 2.34E-09 | 3.57E+01 |
| Asthma | rs146003472 | C | T | 6 | 2.09E-02 | 3.05E-03 | 336782 | 8.77E-12 | 4.66E+01 |
| Asthma | rs148586558 | T | C | 15 | 1.14E-02 | 1.80E-03 | 336782 | 2.36E-10 | 4.01E+01 |
| Asthma | rs150289743 | A | G | 19 | 1.36E-02 | 2.45E-03 | 336782 | 2.75E-08 | 3.09E+01 |
| Asthma | rs150971244 | T | G | 7 | 5.13E-03 | 8.95E-04 | 336782 | 1.01E-08 | 3.28E+01 |
| Asthma | rs1684466 | A | G | 3 | -5.19E-03 | 8.37E-04 | 336782 | 5.59E-10 | 3.85E+01 |
| Asthma | rs174535 | C | T | 11 | -5.12E-03 | 8.16E-04 | 336782 | 3.35E-10 | 3.95E+01 |
| Asthma | rs17454584 | G | A | 4 | 7.27E-03 | 9.41E-04 | 336782 | 1.07E-14 | 5.98E+01 |
| Asthma | rs1789358 | A | G | 11 | 5.50E-03 | 8.59E-04 | 336782 | 1.49E-10 | 4.10E+01 |
| Asthma | rs1837253 | C | T | 5 | 1.14E-02 | 8.87E-04 | 336782 | 4.20E-38 | 1.67E+02 |
| Asthma | rs2025758 | C | T | 10 | -6.05E-03 | 7.83E-04 | 336782 | 1.14E-14 | 5.96E+01 |
| Asthma | rs2197415 | G | T | 10 | 1.02E-02 | 7.89E-04 | 336782 | 5.39E-38 | 1.66E+02 |
| Asthma | rs2296618 | G | A | 1 | -6.63E-03 | 1.15E-03 | 336782 | 7.81E-09 | 3.33E+01 |
| Asthma | rs2299012 | C | A | 5 | 1.04E-02 | 9.88E-04 | 336782 | 4.50E-26 | 1.12E+02 |
| Asthma | rs2338819 | T | C | 5 | -5.24E-03 | 8.59E-04 | 336782 | 1.08E-09 | 3.72E+01 |
| Asthma | rs3024664 | C | T | 16 | 1.25E-02 | 1.64E-03 | 336782 | 2.62E-14 | 5.80E+01 |
| Asthma | rs3024971 | G | T | 12 | -1.31E-02 | 1.26E-03 | 336782 | 2.81E-25 | 1.08E+02 |
| Asthma | rs34173062 | A | G | 8 | 9.71E-03 | 1.60E-03 | 336782 | 1.17E-09 | 3.70E+01 |
| Asthma | rs34290285 | A | G | 2 | -1.10E-02 | 8.92E-04 | 336782 | 9.33E-35 | 1.51E+02 |
| Asthma | rs35570272 | T | G | 3 | 5.59E-03 | 8.00E-04 | 336782 | 2.83E-12 | 4.88E+01 |
| Asthma | rs3781094 | C | A | 10 | -4.74E-03 | 8.28E-04 | 336782 | 1.07E-08 | 3.27E+01 |
| Asthma | rs3784099 | A | G | 14 | 6.13E-03 | 8.67E-04 | 336782 | 1.54E-12 | 5.00E+01 |
| Asthma | rs3806933 | T | C | 5 | -7.06E-03 | 7.86E-04 | 336782 | 2.50E-19 | 8.08E+01 |
| Asthma | rs3807947 | G | T | 7 | -5.03E-03 | 7.95E-04 | 336782 | 2.47E-10 | 4.01E+01 |
| Asthma | rs3825568 | T | C | 14 | -4.70E-03 | 7.81E-04 | 336782 | 1.75E-09 | 3.62E+01 |
| Asthma | rs3856439 | T | C | 2 | -6.04E-03 | 8.23E-04 | 336782 | 2.09E-13 | 5.39E+01 |
| Asthma | rs4073332 | A | G | 16 | 4.87E-03 | 8.19E-04 | 336782 | 2.79E-09 | 3.53E+01 |
| Asthma | rs41283642 | T | C | 9 | -1.34E-02 | 2.15E-03 | 336782 | 5.32E-10 | 3.86E+01 |
| Asthma | rs41284471 | A | G | 10 | 5.74E-03 | 9.71E-04 | 336782 | 3.52E-09 | 3.49E+01 |
| Asthma | rs429916 | A | C | 6 | 8.30E-03 | 1.37E-03 | 336782 | 1.36E-09 | 3.67E+01 |
| Asthma | rs4480384 | G | A | 1 | 4.81E-03 | 8.12E-04 | 336782 | 3.15E-09 | 3.51E+01 |
| Asthma | rs4594881 | T | G | 5 | -4.82E-03 | 8.20E-04 | 336782 | 4.18E-09 | 3.45E+01 |
| Asthma | rs4739738 | A | G | 8 | -7.35E-03 | 8.12E-04 | 336782 | 1.31E-19 | 8.21E+01 |
| Asthma | rs479844 | G | A | 11 | 4.92E-03 | 7.82E-04 | 336782 | 3.05E-10 | 3.96E+01 |
| Asthma | rs4842921 | A | G | 15 | -4.49E-03 | 7.98E-04 | 336782 | 1.79E-08 | 3.17E+01 |
| Asthma | rs55661102 | G | A | 3 | -5.99E-03 | 1.04E-03 | 336782 | 8.49E-09 | 3.32E+01 |
| Asthma | rs58029167 | G | A | 9 | 6.55E-03 | 8.74E-04 | 336782 | 6.58E-14 | 5.62E+01 |
| Asthma | rs61816766 | C | T | 1 | 1.65E-02 | 2.23E-03 | 336782 | 1.35E-13 | 5.48E+01 |
| Asthma | rs61946384 | A | C | 12 | -4.48E-03 | 8.18E-04 | 336782 | 4.30E-08 | 3.00E+01 |
| Asthma | rs6729966 | G | A | 2 | -4.49E-03 | 8.01E-04 | 336782 | 2.16E-08 | 3.13E+01 |
| Asthma | rs7030351 | C | T | 9 | 4.69E-03 | 8.53E-04 | 336782 | 3.75E-08 | 3.03E+01 |
| Asthma | rs7134784 | C | T | 12 | 6.52E-03 | 1.09E-03 | 336782 | 2.36E-09 | 3.56E+01 |
| Asthma | rs7166081 | A | G | 15 | 6.79E-03 | 9.22E-04 | 336782 | 1.73E-13 | 5.43E+01 |
| Asthma | rs72799426 | G | A | 5 | 1.03E-02 | 1.56E-03 | 336782 | 3.46E-11 | 4.39E+01 |
| Asthma | rs75125788 | T | C | 10 | -8.66E-03 | 1.43E-03 | 336782 | 1.46E-09 | 3.66E+01 |
| Asthma | rs75332898 | A | G | 12 | 9.01E-03 | 1.61E-03 | 336782 | 2.23E-08 | 3.13E+01 |
| Asthma | rs76282174 | T | C | 11 | 1.80E-02 | 2.38E-03 | 336782 | 3.54E-14 | 5.74E+01 |
| Asthma | rs7735519 | A | C | 5 | 9.77E-03 | 1.10E-03 | 336782 | 7.08E-19 | 7.88E+01 |
| Asthma | rs8103278 | A | G | 19 | -5.08E-03 | 8.18E-04 | 336782 | 5.02E-10 | 3.87E+01 |
| Asthma | rs840012 | T | C | 1 | -5.94E-03 | 7.95E-04 | 336782 | 8.03E-14 | 5.58E+01 |
| Asthma | rs903361 | A | G | 1 | 5.02E-03 | 8.23E-04 | 336782 | 1.06E-09 | 3.72E+01 |
| Asthma | rs912131 | G | A | 13 | 7.22E-03 | 8.51E-04 | 336782 | 2.19E-17 | 7.20E+01 |
| Asthma | rs917115 | C | T | 7 | 6.72E-03 | 9.58E-04 | 336782 | 2.25E-12 | 4.93E+01 |
| Asthma | rs9344188 | T | C | 6 | 4.61E-03 | 7.83E-04 | 336782 | 3.91E-09 | 3.47E+01 |
| Asthma | rs9368851 | G | A | 6 | 6.43E-03 | 1.05E-03 | 336782 | 1.04E-09 | 3.72E+01 |
| Asthma | rs947591 | A | C | 10 | -4.46E-03 | 7.92E-04 | 336782 | 1.78E-08 | 3.17E+01 |
| Asthma | rs992969 | G | A | 9 | -1.42E-02 | 8.97E-04 | 336782 | 1.12E-56 | 2.52E+02 |

SNPs, single nucleotide polymorphisms

IVs, qualified instrumental variables

CD, Crohn's disease

**Table6 SNPs selected as IVs for UC**

| **exposure** | **SNP** | **effect_allele** | **other_allele** | **chr** | **beta** | **se** | **samplesize** | **pval** | **F_statistic** |
| --- | --- | --- | --- | --- | --- | --- | --- | --- | --- |
| Asthma | rs10456939 | A | G | 6 | -5.09E-03 | 8.99E-04 | 336782 | 1.53E-08 | 3.20E+01 |
| Asthma | rs10795633 | C | T | 10 | -5.33E-03 | 8.01E-04 | 336782 | 2.85E-11 | 4.43E+01 |
| Asthma | rs10836537 | A | G | 11 | -4.53E-03 | 8.19E-04 | 336782 | 3.12E-08 | 3.06E+01 |
| Asthma | rs11071559 | T | C | 15 | -9.58E-03 | 1.16E-03 | 336782 | 1.69E-16 | 6.79E+01 |
| Asthma | rs11178649 | T | G | 12 | -5.11E-03 | 7.93E-04 | 336782 | 1.22E-10 | 4.14E+01 |
| Asthma | rs11255753 | T | G | 10 | 5.80E-03 | 8.69E-04 | 336782 | 2.53E-11 | 4.45E+01 |
| Asthma | rs1136778 | G | A | 6 | 1.82E-02 | 1.50E-03 | 336782 | 7.96E-34 | 1.47E+02 |
| Asthma | rs115018313 | C | T | 6 | 1.60E-02 | 2.55E-03 | 336782 | 4.06E-10 | 3.91E+01 |
| Asthma | rs116548543 | G | T | 6 | -1.82E-02 | 3.07E-03 | 336782 | 3.17E-09 | 3.51E+01 |
| Asthma | rs117710327 | A | C | 19 | -1.52E-02 | 1.59E-03 | 336782 | 8.31E-22 | 9.21E+01 |
| Asthma | rs12123821 | T | C | 1 | 2.02E-02 | 1.82E-03 | 336782 | 8.17E-29 | 1.24E+02 |
| Asthma | rs12356840 | A | G | 10 | 1.02E-02 | 1.59E-03 | 336782 | 1.37E-10 | 4.12E+01 |
| Asthma | rs12365699 | A | G | 11 | -5.74E-03 | 1.05E-03 | 336782 | 4.36E-08 | 3.00E+01 |
| Asthma | rs12700215 | A | G | 7 | -5.25E-03 | 8.13E-04 | 336782 | 1.09E-10 | 4.17E+01 |
| Asthma | rs12788104 | G | A | 11 | 4.63E-03 | 8.41E-04 | 336782 | 3.59E-08 | 3.04E+01 |
| Asthma | rs12952581 | A | G | 17 | 6.58E-03 | 8.10E-04 | 336782 | 4.84E-16 | 6.59E+01 |
| Asthma | rs12964116 | G | A | 18 | 1.17E-02 | 2.10E-03 | 336782 | 2.61E-08 | 3.10E+01 |
| Asthma | rs13208164 | A | G | 6 | 5.22E-03 | 8.81E-04 | 336782 | 3.26E-09 | 3.50E+01 |
| Asthma | rs1321859 | T | C | 6 | -8.36E-03 | 8.20E-04 | 336782 | 2.00E-24 | 1.04E+02 |
| Asthma | rs13355228 | T | C | 5 | 6.86E-03 | 1.15E-03 | 336782 | 2.34E-09 | 3.57E+01 |
| Asthma | rs146003472 | C | T | 6 | 2.09E-02 | 3.05E-03 | 336782 | 8.77E-12 | 4.66E+01 |
| Asthma | rs148586558 | T | C | 15 | 1.14E-02 | 1.80E-03 | 336782 | 2.36E-10 | 4.01E+01 |
| Asthma | rs150289743 | A | G | 19 | 1.36E-02 | 2.45E-03 | 336782 | 2.75E-08 | 3.09E+01 |
| Asthma | rs150971244 | T | G | 7 | 5.13E-03 | 8.95E-04 | 336782 | 1.01E-08 | 3.28E+01 |
| Asthma | rs1684466 | A | G | 3 | -5.19E-03 | 8.37E-04 | 336782 | 5.59E-10 | 3.85E+01 |
| Asthma | rs174535 | C | T | 11 | -5.12E-03 | 8.16E-04 | 336782 | 3.35E-10 | 3.95E+01 |
| Asthma | rs17454584 | G | A | 4 | 7.27E-03 | 9.41E-04 | 336782 | 1.07E-14 | 5.98E+01 |
| Asthma | rs1789358 | A | G | 11 | 5.50E-03 | 8.59E-04 | 336782 | 1.49E-10 | 4.10E+01 |
| Asthma | rs1837253 | C | T | 5 | 1.14E-02 | 8.87E-04 | 336782 | 4.20E-38 | 1.67E+02 |
| Asthma | rs2025758 | C | T | 10 | -6.05E-03 | 7.83E-04 | 336782 | 1.14E-14 | 5.96E+01 |
| Asthma | rs2197415 | G | T | 10 | 1.02E-02 | 7.89E-04 | 336782 | 5.39E-38 | 1.66E+02 |
| Asthma | rs2296618 | G | A | 1 | -6.63E-03 | 1.15E-03 | 336782 | 7.81E-09 | 3.33E+01 |
| Asthma | rs2299012 | C | A | 5 | 1.04E-02 | 9.88E-04 | 336782 | 4.50E-26 | 1.12E+02 |
| Asthma | rs2338819 | T | C | 5 | -5.24E-03 | 8.59E-04 | 336782 | 1.08E-09 | 3.72E+01 |
| Asthma | rs3024664 | C | T | 16 | 1.25E-02 | 1.64E-03 | 336782 | 2.62E-14 | 5.80E+01 |
| Asthma | rs3024971 | G | T | 12 | -1.31E-02 | 1.26E-03 | 336782 | 2.81E-25 | 1.08E+02 |
| Asthma | rs34173062 | A | G | 8 | 9.71E-03 | 1.60E-03 | 336782 | 1.17E-09 | 3.70E+01 |
| Asthma | rs34290285 | A | G | 2 | -1.10E-02 | 8.92E-04 | 336782 | 9.33E-35 | 1.51E+02 |
| Asthma | rs35570272 | T | G | 3 | 5.59E-03 | 8.00E-04 | 336782 | 2.83E-12 | 4.88E+01 |
| Asthma | rs3781094 | C | A | 10 | -4.74E-03 | 8.28E-04 | 336782 | 1.07E-08 | 3.27E+01 |
| Asthma | rs3784099 | A | G | 14 | 6.13E-03 | 8.67E-04 | 336782 | 1.54E-12 | 5.00E+01 |
| Asthma | rs3806933 | T | C | 5 | -7.06E-03 | 7.86E-04 | 336782 | 2.50E-19 | 8.08E+01 |
| Asthma | rs3807947 | G | T | 7 | -5.03E-03 | 7.95E-04 | 336782 | 2.47E-10 | 4.01E+01 |
| Asthma | rs3825568 | T | C | 14 | -4.70E-03 | 7.81E-04 | 336782 | 1.75E-09 | 3.62E+01 |
| Asthma | rs3856439 | T | C | 2 | -6.04E-03 | 8.23E-04 | 336782 | 2.09E-13 | 5.39E+01 |
| Asthma | rs4073332 | A | G | 16 | 4.87E-03 | 8.19E-04 | 336782 | 2.79E-09 | 3.53E+01 |
| Asthma | rs41283642 | T | C | 9 | -1.34E-02 | 2.15E-03 | 336782 | 5.32E-10 | 3.86E+01 |
| Asthma | rs41284471 | A | G | 10 | 5.74E-03 | 9.71E-04 | 336782 | 3.52E-09 | 3.49E+01 |
| Asthma | rs429916 | A | C | 6 | 8.30E-03 | 1.37E-03 | 336782 | 1.36E-09 | 3.67E+01 |
| Asthma | rs4480384 | G | A | 1 | 4.81E-03 | 8.12E-04 | 336782 | 3.15E-09 | 3.51E+01 |
| Asthma | rs4594881 | T | G | 5 | -4.82E-03 | 8.20E-04 | 336782 | 4.18E-09 | 3.45E+01 |
| Asthma | rs479844 | G | A | 11 | 4.92E-03 | 7.82E-04 | 336782 | 3.05E-10 | 3.96E+01 |
| Asthma | rs4842921 | A | G | 15 | -4.49E-03 | 7.98E-04 | 336782 | 1.79E-08 | 3.17E+01 |
| Asthma | rs55661102 | G | A | 3 | -5.99E-03 | 1.04E-03 | 336782 | 8.49E-09 | 3.32E+01 |
| Asthma | rs58029167 | G | A | 9 | 6.55E-03 | 8.74E-04 | 336782 | 6.58E-14 | 5.62E+01 |
| Asthma | rs61816766 | C | T | 1 | 1.65E-02 | 2.23E-03 | 336782 | 1.35E-13 | 5.48E+01 |
| Asthma | rs61946384 | A | C | 12 | -4.48E-03 | 8.18E-04 | 336782 | 4.30E-08 | 3.00E+01 |
| Asthma | rs7030351 | C | T | 9 | 4.69E-03 | 8.53E-04 | 336782 | 3.75E-08 | 3.03E+01 |
| Asthma | rs7134784 | C | T | 12 | 6.52E-03 | 1.09E-03 | 336782 | 2.36E-09 | 3.56E+01 |
| Asthma | rs7166081 | A | G | 15 | 6.79E-03 | 9.22E-04 | 336782 | 1.73E-13 | 5.43E+01 |
| Asthma | rs72799426 | G | A | 5 | 1.03E-02 | 1.56E-03 | 336782 | 3.46E-11 | 4.39E+01 |
| Asthma | rs75125788 | T | C | 10 | -8.66E-03 | 1.43E-03 | 336782 | 1.46E-09 | 3.66E+01 |
| Asthma | rs75332898 | A | G | 12 | 9.01E-03 | 1.61E-03 | 336782 | 2.23E-08 | 3.13E+01 |
| Asthma | rs76282174 | T | C | 11 | 1.80E-02 | 2.38E-03 | 336782 | 3.54E-14 | 5.74E+01 |
| Asthma | rs7735519 | A | C | 5 | 9.77E-03 | 1.10E-03 | 336782 | 7.08E-19 | 7.88E+01 |
| Asthma | rs8103278 | A | G | 19 | -5.08E-03 | 8.18E-04 | 336782 | 5.02E-10 | 3.87E+01 |
| Asthma | rs840012 | T | C | 1 | -5.94E-03 | 7.95E-04 | 336782 | 8.03E-14 | 5.58E+01 |
| Asthma | rs903361 | A | G | 1 | 5.02E-03 | 8.23E-04 | 336782 | 1.06E-09 | 3.72E+01 |
| Asthma | rs912131 | G | A | 13 | 7.22E-03 | 8.51E-04 | 336782 | 2.19E-17 | 7.20E+01 |
| Asthma | rs917115 | C | T | 7 | 6.72E-03 | 9.58E-04 | 336782 | 2.25E-12 | 4.93E+01 |
| Asthma | rs9344188 | T | C | 6 | 4.61E-03 | 7.83E-04 | 336782 | 3.91E-09 | 3.47E+01 |
| Asthma | rs9368851 | G | A | 6 | 6.43E-03 | 1.05E-03 | 336782 | 1.04E-09 | 3.72E+01 |
| Asthma | rs947591 | A | C | 10 | -4.46E-03 | 7.92E-04 | 336782 | 1.78E-08 | 3.17E+01 |
| Asthma | rs992969 | G | A | 9 | -1.42E-02 | 8.97E-04 | 336782 | 1.12E-56 | 2.52E+02 |

SNPs, single nucleotide polymorphisms

IVs, qualified instrumental variables

UC, ulcerative colitis

**Table7 SNPs selected as IVs for IBD**

| **exposure** | **SNP** | **effect_allele** | **other_allele** | **chr** | **beta** | **se** | **samplesize** | **pval** | **F_statistic** |
| --- | --- | --- | --- | --- | --- | --- | --- | --- | --- |
| AD | rs1012415 | C | A | 5 | 5.60E-02 | 1.06E-02 | 796661 | 1.17E-07 | 2.79E+01 |
| AD | rs11033603 | A | G | 11 | 1.65E-01 | 2.32E-02 | 796661 | 1.33E-12 | 5.03E+01 |
| AD | rs11130215 | G | A | 3 | -6.07E-02 | 1.20E-02 | 796661 | 4.03E-07 | 2.56E+01 |
| AD | rs113537508 | A | G | 5 | 8.11E-02 | 1.66E-02 | 796661 | 9.72E-07 | 2.39E+01 |
| AD | rs11649344 | T | C | 16 | -8.61E-02 | 1.81E-02 | 796661 | 1.99E-06 | 2.26E+01 |
| AD | rs117137535 | A | G | 9 | 1.50E-01 | 2.99E-02 | 796661 | 5.32E-07 | 2.52E+01 |
| AD | rs11786685 | G | A | 8 | -7.21E-02 | 1.08E-02 | 796661 | 1.99E-11 | 4.46E+01 |
| AD | rs12123821 | T | C | 1 | 2.70E-01 | 2.54E-02 | 796661 | 2.20E-26 | 1.13E+02 |
| AD | rs12126142 | A | G | 1 | 6.26E-02 | 1.08E-02 | 796661 | 6.21E-09 | 3.36E+01 |
| AD | rs12409961 | G | A | 1 | -5.73E-02 | 1.23E-02 | 796661 | 3.05E-06 | 2.17E+01 |
| AD | rs12544266 | G | A | 8 | 6.44E-02 | 1.26E-02 | 796661 | 3.46E-07 | 2.61E+01 |
| AD | rs12586305 | G | T | 14 | 9.10E-02 | 1.40E-02 | 796661 | 7.92E-11 | 4.22E+01 |
| AD | rs13222852 | T | C | 7 | 7.17E-02 | 1.47E-02 | 796661 | 1.02E-06 | 2.38E+01 |
| AD | rs13343107 | G | A | 1 | 7.54E-02 | 1.21E-02 | 796661 | 5.11E-10 | 3.88E+01 |
| AD | rs142614912 | T | G | 20 | 1.18E-01 | 2.30E-02 | 796661 | 3.16E-07 | 2.62E+01 |
| AD | rs142795541 | A | G | 11 | -4.49E-01 | 9.83E-02 | 796661 | 4.97E-06 | 2.08E+01 |
| AD | rs147451981 | T | G | 1 | -1.87E-01 | 3.97E-02 | 796661 | 2.48E-06 | 2.22E+01 |
| AD | rs1504215 | A | G | 6 | -5.70E-02 | 1.14E-02 | 796661 | 6.16E-07 | 2.50E+01 |
| AD | rs16843927 | G | T | 3 | 4.90E-02 | 1.04E-02 | 796661 | 2.27E-06 | 2.22E+01 |
| AD | rs16949006 | T | C | 16 | -1.80E-01 | 3.83E-02 | 796661 | 2.68E-06 | 2.20E+01 |
| AD | rs17371133 | C | A | 1 | 6.51E-02 | 1.02E-02 | 796661 | 2.07E-10 | 4.07E+01 |
| AD | rs17881320 | T | G | 17 | 8.98E-02 | 1.92E-02 | 796661 | 2.79E-06 | 2.19E+01 |
| AD | rs2075943 | A | G | 22 | 6.48E-02 | 1.08E-02 | 796661 | 2.09E-09 | 3.60E+01 |
| AD | rs271620 | C | A | 19 | 6.70E-02 | 1.28E-02 | 796661 | 1.70E-07 | 2.74E+01 |
| AD | rs28383323 | A | G | 6 | -1.19E-01 | 1.60E-02 | 796661 | 1.10E-13 | 5.51E+01 |
| AD | rs285257 | G | A | 19 | -5.11E-02 | 1.11E-02 | 796661 | 4.19E-06 | 2.12E+01 |
| AD | rs28538178 | G | T | 1 | 2.05E-01 | 4.40E-02 | 796661 | 3.09E-06 | 2.17E+01 |
| AD | rs28752924 | C | T | 6 | -9.85E-02 | 1.50E-02 | 796661 | 4.95E-11 | 4.31E+01 |
| AD | rs2918302 | A | G | 19 | 9.15E-02 | 1.33E-02 | 796661 | 5.10E-12 | 4.73E+01 |
| AD | rs3135024 | C | T | 6 | 5.94E-02 | 1.28E-02 | 796661 | 3.31E-06 | 2.15E+01 |
| AD | rs34064097 | A | G | 10 | -5.51E-02 | 1.09E-02 | 796661 | 4.53E-07 | 2.56E+01 |
| AD | rs35073649 | T | C | 17 | 7.85E-02 | 1.03E-02 | 796661 | 2.04E-14 | 5.81E+01 |
| AD | rs353397 | A | G | 5 | 5.11E-02 | 1.02E-02 | 796661 | 5.92E-07 | 2.51E+01 |
| AD | rs3793638 | T | G | 9 | -4.82E-02 | 1.05E-02 | 796661 | 4.21E-06 | 2.11E+01 |
| AD | rs3847804 | T | C | 12 | 6.70E-02 | 1.30E-02 | 796661 | 2.59E-07 | 2.66E+01 |
| AD | rs3862469 | T | C | 16 | -7.59E-02 | 1.09E-02 | 796661 | 3.59E-12 | 4.85E+01 |
| AD | rs3947727 | T | C | 12 | 7.14E-02 | 1.05E-02 | 796661 | 1.09E-11 | 4.62E+01 |
| AD | rs479844 | G | A | 11 | 7.19E-02 | 1.03E-02 | 796661 | 2.41E-12 | 4.87E+01 |
| AD | rs4842704 | G | A | 12 | -2.66E-01 | 5.79E-02 | 796661 | 4.56E-06 | 2.10E+01 |
| AD | rs553644 | A | G | 6 | 6.09E-02 | 1.28E-02 | 796661 | 1.88E-06 | 2.26E+01 |
| AD | rs57248180 | T | C | 7 | -6.47E-02 | 1.21E-02 | 796661 | 9.42E-08 | 2.86E+01 |
| AD | rs57389015 | C | T | 20 | 1.16E-01 | 2.50E-02 | 796661 | 3.40E-06 | 2.16E+01 |
| AD | rs61889950 | G | A | 11 | 6.15E-02 | 1.24E-02 | 796661 | 7.57E-07 | 2.46E+01 |
| AD | rs61893488 | A | G | 11 | -1.17E-01 | 1.76E-02 | 796661 | 2.91E-11 | 4.41E+01 |
| AD | rs61898347 | A | G | 11 | 6.43E-02 | 1.25E-02 | 796661 | 2.68E-07 | 2.65E+01 |
| AD | rs61907710 | T | C | 11 | -6.19E-02 | 1.16E-02 | 796661 | 1.00E-07 | 2.85E+01 |
| AD | rs61922516 | A | G | 12 | 3.38E-01 | 6.65E-02 | 796661 | 3.86E-07 | 2.58E+01 |
| AD | rs6996614 | A | C | 8 | 8.05E-02 | 1.42E-02 | 796661 | 1.55E-08 | 3.21E+01 |
| AD | rs7133768 | C | T | 12 | -5.20E-02 | 1.13E-02 | 796661 | 4.55E-06 | 2.12E+01 |
| AD | rs72957353 | T | C | 3 | -1.59E-01 | 3.14E-02 | 796661 | 4.07E-07 | 2.56E+01 |
| AD | rs7521672 | G | A | 1 | -1.67E-01 | 3.44E-02 | 796661 | 1.27E-06 | 2.35E+01 |
| AD | rs7522243 | G | A | 1 | -5.30E-02 | 1.03E-02 | 796661 | 2.83E-07 | 2.65E+01 |
| AD | rs75843100 | T | C | 5 | -7.60E-02 | 1.54E-02 | 796661 | 8.59E-07 | 2.44E+01 |
| AD | rs76936650 | C | T | 3 | -1.71E-01 | 3.70E-02 | 796661 | 3.68E-06 | 2.14E+01 |
| AD | rs7699742 | C | T | 4 | 6.03E-02 | 1.03E-02 | 796661 | 5.45E-09 | 3.43E+01 |
| AD | rs7925585 | G | A | 11 | -5.06E-02 | 1.06E-02 | 796661 | 1.81E-06 | 2.28E+01 |
| AD | rs847 | C | T | 5 | -9.33E-02 | 1.11E-02 | 796661 | 3.75E-17 | 7.07E+01 |
| AD | rs903203 | A | G | 16 | 5.86E-02 | 1.13E-02 | 796661 | 2.16E-07 | 2.69E+01 |
| AD | rs943451 | C | T | 10 | -5.59E-02 | 1.16E-02 | 796661 | 1.34E-06 | 2.32E+01 |
| AD | rs9435973 | G | A | 1 | 5.57E-02 | 1.18E-02 | 796661 | 2.33E-06 | 2.23E+01 |
| AD | rs9889953 | A | G | 17 | 5.25E-02 | 1.05E-02 | 796661 | 5.53E-07 | 2.50E+01 |

SNPs, single nucleotide polymorphisms

IVs, qualified instrumental variables

IBD, inflammatory bowel disease

AD, atopic dermatitis

**Table8 SNPs selected as IVs for CD**

| **exposure** | **SNP** | **effect_allele** | **other_allele** | **chr** | **beta** | **se** | **samplesize** | **pval** | **F_statistic** |
| --- | --- | --- | --- | --- | --- | --- | --- | --- | --- |
| AD | rs1012415 | C | A | 5 | 5.60E-02 | 1.06E-02 | 796661 | 1.17E-07 | 2.79E+01 |
| AD | rs11033603 | A | G | 11 | 1.65E-01 | 2.32E-02 | 796661 | 1.33E-12 | 5.03E+01 |
| AD | rs11130215 | G | A | 3 | -6.07E-02 | 1.20E-02 | 796661 | 4.03E-07 | 2.56E+01 |
| AD | rs113537508 | A | G | 5 | 8.11E-02 | 1.66E-02 | 796661 | 9.72E-07 | 2.39E+01 |
| AD | rs11649344 | T | C | 16 | -8.61E-02 | 1.81E-02 | 796661 | 1.99E-06 | 2.26E+01 |
| AD | rs117137535 | A | G | 9 | 1.50E-01 | 2.99E-02 | 796661 | 5.32E-07 | 2.52E+01 |
| AD | rs11786685 | G | A | 8 | -7.21E-02 | 1.08E-02 | 796661 | 1.99E-11 | 4.46E+01 |
| AD | rs12123821 | T | C | 1 | 2.70E-01 | 2.54E-02 | 796661 | 2.20E-26 | 1.13E+02 |
| AD | rs12126142 | A | G | 1 | 6.26E-02 | 1.08E-02 | 796661 | 6.21E-09 | 3.36E+01 |
| AD | rs12409961 | G | A | 1 | -5.73E-02 | 1.23E-02 | 796661 | 3.05E-06 | 2.17E+01 |
| AD | rs12544266 | G | A | 8 | 6.44E-02 | 1.26E-02 | 796661 | 3.46E-07 | 2.61E+01 |
| AD | rs12586305 | G | T | 14 | 9.10E-02 | 1.40E-02 | 796661 | 7.92E-11 | 4.22E+01 |
| AD | rs13222852 | T | C | 7 | 7.17E-02 | 1.47E-02 | 796661 | 1.02E-06 | 2.38E+01 |
| AD | rs13343107 | G | A | 1 | 7.54E-02 | 1.21E-02 | 796661 | 5.11E-10 | 3.88E+01 |
| AD | rs142614912 | T | G | 20 | 1.18E-01 | 2.30E-02 | 796661 | 3.16E-07 | 2.62E+01 |
| AD | rs142795541 | A | G | 11 | -4.49E-01 | 9.83E-02 | 796661 | 4.97E-06 | 2.08E+01 |
| AD | rs147451981 | T | G | 1 | -1.87E-01 | 3.97E-02 | 796661 | 2.48E-06 | 2.22E+01 |
| AD | rs1504215 | A | G | 6 | -5.70E-02 | 1.14E-02 | 796661 | 6.16E-07 | 2.50E+01 |
| AD | rs16843927 | G | T | 3 | 4.90E-02 | 1.04E-02 | 796661 | 2.27E-06 | 2.22E+01 |
| AD | rs16949006 | T | C | 16 | -1.80E-01 | 3.83E-02 | 796661 | 2.68E-06 | 2.20E+01 |
| AD | rs17371133 | C | A | 1 | 6.51E-02 | 1.02E-02 | 796661 | 2.07E-10 | 4.07E+01 |
| AD | rs17881320 | T | G | 17 | 8.98E-02 | 1.92E-02 | 796661 | 2.79E-06 | 2.19E+01 |
| AD | rs2075943 | A | G | 22 | 6.48E-02 | 1.08E-02 | 796661 | 2.09E-09 | 3.60E+01 |
| AD | rs271620 | C | A | 19 | 6.70E-02 | 1.28E-02 | 796661 | 1.70E-07 | 2.74E+01 |
| AD | rs28383323 | A | G | 6 | -1.19E-01 | 1.60E-02 | 796661 | 1.10E-13 | 5.51E+01 |
| AD | rs285257 | G | A | 19 | -5.11E-02 | 1.11E-02 | 796661 | 4.19E-06 | 2.12E+01 |
| AD | rs28538178 | G | T | 1 | 2.05E-01 | 4.40E-02 | 796661 | 3.09E-06 | 2.17E+01 |
| AD | rs28752924 | C | T | 6 | -9.85E-02 | 1.50E-02 | 796661 | 4.95E-11 | 4.31E+01 |
| AD | rs2918302 | A | G | 19 | 9.15E-02 | 1.33E-02 | 796661 | 5.10E-12 | 4.73E+01 |
| AD | rs3135024 | C | T | 6 | 5.94E-02 | 1.28E-02 | 796661 | 3.31E-06 | 2.15E+01 |
| AD | rs34064097 | A | G | 10 | -5.51E-02 | 1.09E-02 | 796661 | 4.53E-07 | 2.56E+01 |
| AD | rs35073649 | T | C | 17 | 7.85E-02 | 1.03E-02 | 796661 | 2.04E-14 | 5.81E+01 |
| AD | rs353397 | A | G | 5 | 5.11E-02 | 1.02E-02 | 796661 | 5.92E-07 | 2.51E+01 |
| AD | rs3793638 | T | G | 9 | -4.82E-02 | 1.05E-02 | 796661 | 4.21E-06 | 2.11E+01 |
| AD | rs3847804 | T | C | 12 | 6.70E-02 | 1.30E-02 | 796661 | 2.59E-07 | 2.66E+01 |
| AD | rs3862469 | T | C | 16 | -7.59E-02 | 1.09E-02 | 796661 | 3.59E-12 | 4.85E+01 |
| AD | rs3947727 | T | C | 12 | 7.14E-02 | 1.05E-02 | 796661 | 1.09E-11 | 4.62E+01 |
| AD | rs479844 | G | A | 11 | 7.19E-02 | 1.03E-02 | 796661 | 2.41E-12 | 4.87E+01 |
| AD | rs4842704 | G | A | 12 | -2.66E-01 | 5.79E-02 | 796661 | 4.56E-06 | 2.10E+01 |
| AD | rs553644 | A | G | 6 | 6.09E-02 | 1.28E-02 | 796661 | 1.88E-06 | 2.26E+01 |
| AD | rs57248180 | T | C | 7 | -6.47E-02 | 1.21E-02 | 796661 | 9.42E-08 | 2.86E+01 |
| AD | rs57389015 | C | T | 20 | 1.16E-01 | 2.50E-02 | 796661 | 3.40E-06 | 2.16E+01 |
| AD | rs61889950 | G | A | 11 | 6.15E-02 | 1.24E-02 | 796661 | 7.57E-07 | 2.46E+01 |
| AD | rs61893488 | A | G | 11 | -1.17E-01 | 1.76E-02 | 796661 | 2.91E-11 | 4.41E+01 |
| AD | rs61898347 | A | G | 11 | 6.43E-02 | 1.25E-02 | 796661 | 2.68E-07 | 2.65E+01 |
| AD | rs61907710 | T | C | 11 | -6.19E-02 | 1.16E-02 | 796661 | 1.00E-07 | 2.85E+01 |
| AD | rs61922516 | A | G | 12 | 3.38E-01 | 6.65E-02 | 796661 | 3.86E-07 | 2.58E+01 |
| AD | rs6996614 | A | C | 8 | 8.05E-02 | 1.42E-02 | 796661 | 1.55E-08 | 3.21E+01 |
| AD | rs7133768 | C | T | 12 | -5.20E-02 | 1.13E-02 | 796661 | 4.55E-06 | 2.12E+01 |
| AD | rs72957353 | T | C | 3 | -1.59E-01 | 3.14E-02 | 796661 | 4.07E-07 | 2.56E+01 |
| AD | rs7521672 | G | A | 1 | -1.67E-01 | 3.44E-02 | 796661 | 1.27E-06 | 2.35E+01 |
| AD | rs7522243 | G | A | 1 | -5.30E-02 | 1.03E-02 | 796661 | 2.83E-07 | 2.65E+01 |
| AD | rs75843100 | T | C | 5 | -7.60E-02 | 1.54E-02 | 796661 | 8.59E-07 | 2.44E+01 |
| AD | rs76936650 | C | T | 3 | -1.71E-01 | 3.70E-02 | 796661 | 3.68E-06 | 2.14E+01 |
| AD | rs7699742 | C | T | 4 | 6.03E-02 | 1.03E-02 | 796661 | 5.45E-09 | 3.43E+01 |
| AD | rs7925585 | G | A | 11 | -5.06E-02 | 1.06E-02 | 796661 | 1.81E-06 | 2.28E+01 |
| AD | rs847 | C | T | 5 | -9.33E-02 | 1.11E-02 | 796661 | 3.75E-17 | 7.07E+01 |
| AD | rs903203 | A | G | 16 | 5.86E-02 | 1.13E-02 | 796661 | 2.16E-07 | 2.69E+01 |
| AD | rs943451 | C | T | 10 | -5.59E-02 | 1.16E-02 | 796661 | 1.34E-06 | 2.32E+01 |
| AD | rs9435973 | G | A | 1 | 5.57E-02 | 1.18E-02 | 796661 | 2.33E-06 | 2.23E+01 |
| AD | rs9889953 | A | G | 17 | 5.25E-02 | 1.05E-02 | 796661 | 5.53E-07 | 2.50E+01 |

SNPs, single nucleotide polymorphisms

IVs, qualified instrumental variables

CD, Crohn's disease

AD, atopic dermatitis

**Table9 SNPs selected as IVs for UC**

| **exposure** | **SNP** | **effect_allele** | **other_allele** | **chr** | **beta** | **se** | **samplesize** | **pval** | **F_statistic** |
| --- | --- | --- | --- | --- | --- | --- | --- | --- | --- |
| AD | rs1012415 | C | A | 5 | 5.60E-02 | 1.06E-02 | 796661 | 1.17E-07 | 2.79E+01 |
| AD | rs11033603 | A | G | 11 | 1.65E-01 | 2.32E-02 | 796661 | 1.33E-12 | 5.03E+01 |
| AD | rs11130215 | G | A | 3 | -6.07E-02 | 1.20E-02 | 796661 | 4.03E-07 | 2.56E+01 |
| AD | rs113537508 | A | G | 5 | 8.11E-02 | 1.66E-02 | 796661 | 9.72E-07 | 2.39E+01 |
| AD | rs11649344 | T | C | 16 | -8.61E-02 | 1.81E-02 | 796661 | 1.99E-06 | 2.26E+01 |
| AD | rs117137535 | A | G | 9 | 1.50E-01 | 2.99E-02 | 796661 | 5.32E-07 | 2.52E+01 |
| AD | rs11786685 | G | A | 8 | -7.21E-02 | 1.08E-02 | 796661 | 1.99E-11 | 4.46E+01 |
| AD | rs12123821 | T | C | 1 | 2.70E-01 | 2.54E-02 | 796661 | 2.20E-26 | 1.13E+02 |
| AD | rs12126142 | A | G | 1 | 6.26E-02 | 1.08E-02 | 796661 | 6.21E-09 | 3.36E+01 |
| AD | rs12409961 | G | A | 1 | -5.73E-02 | 1.23E-02 | 796661 | 3.05E-06 | 2.17E+01 |
| AD | rs12544266 | G | A | 8 | 6.44E-02 | 1.26E-02 | 796661 | 3.46E-07 | 2.61E+01 |
| AD | rs12586305 | G | T | 14 | 9.10E-02 | 1.40E-02 | 796661 | 7.92E-11 | 4.22E+01 |
| AD | rs13222852 | T | C | 7 | 7.17E-02 | 1.47E-02 | 796661 | 1.02E-06 | 2.38E+01 |
| AD | rs13343107 | G | A | 1 | 7.54E-02 | 1.21E-02 | 796661 | 5.11E-10 | 3.88E+01 |
| AD | rs142614912 | T | G | 20 | 1.18E-01 | 2.30E-02 | 796661 | 3.16E-07 | 2.62E+01 |
| AD | rs142795541 | A | G | 11 | -4.49E-01 | 9.83E-02 | 796661 | 4.97E-06 | 2.08E+01 |
| AD | rs147451981 | T | G | 1 | -1.87E-01 | 3.97E-02 | 796661 | 2.48E-06 | 2.22E+01 |
| AD | rs1504215 | A | G | 6 | -5.70E-02 | 1.14E-02 | 796661 | 6.16E-07 | 2.50E+01 |
| AD | rs16843927 | G | T | 3 | 4.90E-02 | 1.04E-02 | 796661 | 2.27E-06 | 2.22E+01 |
| AD | rs16949006 | T | C | 16 | -1.80E-01 | 3.83E-02 | 796661 | 2.68E-06 | 2.20E+01 |
| AD | rs17371133 | C | A | 1 | 6.51E-02 | 1.02E-02 | 796661 | 2.07E-10 | 4.07E+01 |
| AD | rs17881320 | T | G | 17 | 8.98E-02 | 1.92E-02 | 796661 | 2.79E-06 | 2.19E+01 |
| AD | rs1861246 | C | T | 2 | -1.13E-01 | 1.22E-02 | 796661 | 1.51E-20 | 8.64E+01 |
| AD | rs2075943 | A | G | 22 | 6.48E-02 | 1.08E-02 | 796661 | 2.09E-09 | 3.60E+01 |
| AD | rs271620 | C | A | 19 | 6.70E-02 | 1.28E-02 | 796661 | 1.70E-07 | 2.74E+01 |
| AD | rs28383323 | A | G | 6 | -1.19E-01 | 1.60E-02 | 796661 | 1.10E-13 | 5.51E+01 |
| AD | rs285257 | G | A | 19 | -5.11E-02 | 1.11E-02 | 796661 | 4.19E-06 | 2.12E+01 |
| AD | rs28538178 | G | T | 1 | 2.05E-01 | 4.40E-02 | 796661 | 3.09E-06 | 2.17E+01 |
| AD | rs28752924 | C | T | 6 | -9.85E-02 | 1.50E-02 | 796661 | 4.95E-11 | 4.31E+01 |
| AD | rs2918302 | A | G | 19 | 9.15E-02 | 1.33E-02 | 796661 | 5.10E-12 | 4.73E+01 |
| AD | rs3135024 | C | T | 6 | 5.94E-02 | 1.28E-02 | 796661 | 3.31E-06 | 2.15E+01 |
| AD | rs34064097 | A | G | 10 | -5.51E-02 | 1.09E-02 | 796661 | 4.53E-07 | 2.56E+01 |
| AD | rs35073649 | T | C | 17 | 7.85E-02 | 1.03E-02 | 796661 | 2.04E-14 | 5.81E+01 |
| AD | rs353397 | A | G | 5 | 5.11E-02 | 1.02E-02 | 796661 | 5.92E-07 | 2.51E+01 |
| AD | rs3793638 | T | G | 9 | -4.82E-02 | 1.05E-02 | 796661 | 4.21E-06 | 2.11E+01 |
| AD | rs3847804 | T | C | 12 | 6.70E-02 | 1.30E-02 | 796661 | 2.59E-07 | 2.66E+01 |
| AD | rs3862469 | T | C | 16 | -7.59E-02 | 1.09E-02 | 796661 | 3.59E-12 | 4.85E+01 |
| AD | rs3947727 | T | C | 12 | 7.14E-02 | 1.05E-02 | 796661 | 1.09E-11 | 4.62E+01 |
| AD | rs479844 | G | A | 11 | 7.19E-02 | 1.03E-02 | 796661 | 2.41E-12 | 4.87E+01 |
| AD | rs4842704 | G | A | 12 | -2.66E-01 | 5.79E-02 | 796661 | 4.56E-06 | 2.10E+01 |
| AD | rs553644 | A | G | 6 | 6.09E-02 | 1.28E-02 | 796661 | 1.88E-06 | 2.26E+01 |
| AD | rs57248180 | T | C | 7 | -6.47E-02 | 1.21E-02 | 796661 | 9.42E-08 | 2.86E+01 |
| AD | rs57389015 | C | T | 20 | 1.16E-01 | 2.50E-02 | 796661 | 3.40E-06 | 2.16E+01 |
| AD | rs61839660 | T | C | 10 | 1.14E-01 | 2.23E-02 | 796661 | 2.98E-07 | 2.62E+01 |
| AD | rs61889950 | G | A | 11 | 6.15E-02 | 1.24E-02 | 796661 | 7.57E-07 | 2.46E+01 |
| AD | rs61893488 | A | G | 11 | -1.17E-01 | 1.76E-02 | 796661 | 2.91E-11 | 4.41E+01 |
| AD | rs61898347 | A | G | 11 | 6.43E-02 | 1.25E-02 | 796661 | 2.68E-07 | 2.65E+01 |
| AD | rs61907710 | T | C | 11 | -6.19E-02 | 1.16E-02 | 796661 | 1.00E-07 | 2.85E+01 |
| AD | rs61922516 | A | G | 12 | 3.38E-01 | 6.65E-02 | 796661 | 3.86E-07 | 2.58E+01 |
| AD | rs6996614 | A | C | 8 | 8.05E-02 | 1.42E-02 | 796661 | 1.55E-08 | 3.21E+01 |
| AD | rs7133768 | C | T | 12 | -5.20E-02 | 1.13E-02 | 796661 | 4.55E-06 | 2.12E+01 |
| AD | rs72957353 | T | C | 3 | -1.59E-01 | 3.14E-02 | 796661 | 4.07E-07 | 2.56E+01 |
| AD | rs7521672 | G | A | 1 | -1.67E-01 | 3.44E-02 | 796661 | 1.27E-06 | 2.35E+01 |
| AD | rs7522243 | G | A | 1 | -5.30E-02 | 1.03E-02 | 796661 | 2.83E-07 | 2.65E+01 |
| AD | rs75843100 | T | C | 5 | -7.60E-02 | 1.54E-02 | 796661 | 8.59E-07 | 2.44E+01 |
| AD | rs76936650 | C | T | 3 | -1.71E-01 | 3.70E-02 | 796661 | 3.68E-06 | 2.14E+01 |
| AD | rs7699742 | C | T | 4 | 6.03E-02 | 1.03E-02 | 796661 | 5.45E-09 | 3.43E+01 |
| AD | rs7925585 | G | A | 11 | -5.06E-02 | 1.06E-02 | 796661 | 1.81E-06 | 2.28E+01 |
| AD | rs847 | C | T | 5 | -9.33E-02 | 1.11E-02 | 796661 | 3.75E-17 | 7.07E+01 |
| AD | rs903203 | A | G | 16 | 5.86E-02 | 1.13E-02 | 796661 | 2.16E-07 | 2.69E+01 |
| AD | rs943451 | C | T | 10 | -5.59E-02 | 1.16E-02 | 796661 | 1.34E-06 | 2.32E+01 |
| AD | rs9435973 | G | A | 1 | 5.57E-02 | 1.18E-02 | 796661 | 2.33E-06 | 2.23E+01 |
| AD | rs9889953 | A | G | 17 | 5.25E-02 | 1.05E-02 | 796661 | 5.53E-07 | 2.50E+01 |

SNPs, single nucleotide polymorphisms

IVs, qualified instrumental variables

UC, ulcerative colitis

AD, atopic dermatitis

**Table10 SNPs selected as IVs for AR**

| **exposure** | **SNP** | **effect_allele** | **other_allele** | **chr** | **beta** | **se** | **samplesize** | **pval** | **F_statistic** |
| --- | --- | --- | --- | --- | --- | --- | --- | --- | --- |
| IBD | rs10246518 | A | G | 7 | 1.57E-01 | 2.95E-02 | 218792 | 1.06E-07 | 2.83E+01 |
| IBD | rs10737481 | G | T | 1 | 1.23E-01 | 1.99E-02 | 218793 | 6.20E-10 | 3.81E+01 |
| IBD | rs10748781 | A | C | 10 | -1.33E-01 | 2.09E-02 | 218794 | 1.97E-10 | 4.07E+01 |
| IBD | rs10761659 | G | A | 10 | 9.63E-02 | 1.99E-02 | 218795 | 1.26E-06 | 2.34E+01 |
| IBD | rs10807943 | C | T | 7 | -3.67E-01 | 4.25E-02 | 218796 | 6.20E-18 | 7.44E+01 |
| IBD | rs11204894 | T | G | 1 | -1.22E-01 | 2.23E-02 | 218797 | 5.22E-08 | 2.97E+01 |
| IBD | rs112266302 | A | G | 7 | 1.84E-01 | 2.78E-02 | 218798 | 3.23E-11 | 4.39E+01 |
| IBD | rs11244204 | G | A | 9 | -1.93E-01 | 4.11E-02 | 218799 | 2.56E-06 | 2.21E+01 |
| IBD | rs11583398 | A | C | 1 | 1.37E-01 | 2.83E-02 | 218800 | 1.45E-06 | 2.33E+01 |
| IBD | rs116929608 | G | A | 7 | 2.71E-01 | 3.85E-02 | 218801 | 2.08E-12 | 4.94E+01 |
| IBD | rs117245455 | G | T | 17 | 3.50E-01 | 7.64E-02 | 218802 | 4.47E-06 | 2.10E+01 |
| IBD | rs117959250 | T | G | 7 | 2.46E-01 | 4.92E-02 | 218803 | 5.67E-07 | 2.50E+01 |
| IBD | rs12175489 | A | G | 6 | 1.45E-01 | 2.76E-02 | 218804 | 1.57E-07 | 2.75E+01 |
| IBD | rs12581964 | T | C | 12 | 2.46E-01 | 5.37E-02 | 218805 | 4.59E-06 | 2.10E+01 |
| IBD | rs13300483 | T | C | 9 | 9.82E-02 | 2.12E-02 | 218806 | 3.56E-06 | 2.15E+01 |
| IBD | rs139348065 | A | G | 1 | 5.60E-01 | 1.17E-01 | 218807 | 1.67E-06 | 2.29E+01 |
| IBD | rs145568234 | G | T | 6 | 1.31E+00 | 2.03E-01 | 218808 | 1.10E-10 | 4.16E+01 |
| IBD | rs146249753 | T | C | 7 | 1.74E-01 | 3.11E-02 | 218809 | 2.26E-08 | 3.12E+01 |
| IBD | rs149368937 | G | A | 5 | 1.38E-01 | 2.68E-02 | 218810 | 2.48E-07 | 2.66E+01 |
| IBD | rs17689550 | T | C | 5 | -1.34E-01 | 2.45E-02 | 218811 | 4.68E-08 | 2.99E+01 |
| IBD | rs1801274 | G | A | 1 | -1.06E-01 | 1.98E-02 | 218812 | 8.89E-08 | 2.87E+01 |
| IBD | rs2149560 | T | G | 9 | 1.10E-01 | 2.00E-02 | 218813 | 3.63E-08 | 3.02E+01 |
| IBD | rs2480428 | G | A | 13 | -9.53E-02 | 2.03E-02 | 218814 | 2.63E-06 | 2.20E+01 |
| IBD | rs2836878 | A | G | 21 | -1.19E-01 | 2.31E-02 | 218815 | 2.96E-07 | 2.64E+01 |
| IBD | rs3197999 | A | G | 3 | 1.31E-01 | 2.04E-02 | 218816 | 1.38E-10 | 4.12E+01 |
| IBD | rs3799396 | G | A | 6 | 2.55E-01 | 5.55E-02 | 218817 | 4.42E-06 | 2.11E+01 |
| IBD | rs3823375 | C | T | 6 | 1.21E-01 | 2.05E-02 | 218818 | 4.30E-09 | 3.46E+01 |
| IBD | rs4259333 | T | C | 7 | -1.10E-01 | 2.38E-02 | 218819 | 3.96E-06 | 2.12E+01 |
| IBD | rs4676410 | A | G | 2 | 1.41E-01 | 2.25E-02 | 218820 | 3.16E-10 | 3.95E+01 |
| IBD | rs56116661 | T | C | 3 | -1.22E-01 | 2.39E-02 | 218821 | 3.66E-07 | 2.59E+01 |
| IBD | rs56343836 | A | G | 13 | 1.99E+00 | 4.12E-01 | 218822 | 1.37E-06 | 2.33E+01 |
| IBD | rs6062496 | A | G | 20 | 1.05E-01 | 2.08E-02 | 218823 | 5.16E-07 | 2.53E+01 |
| IBD | rs61997160 | A | G | 7 | 2.42E-01 | 3.40E-02 | 218824 | 1.10E-12 | 5.08E+01 |
| IBD | rs62443225 | A | G | 7 | 3.84E-01 | 3.87E-02 | 218825 | 3.37E-23 | 9.84E+01 |
| IBD | rs6696981 | T | G | 1 | -1.84E-01 | 3.77E-02 | 218826 | 1.09E-06 | 2.37E+01 |
| IBD | rs6966158 | A | C | 7 | -1.78E-01 | 3.42E-02 | 218827 | 1.94E-07 | 2.71E+01 |
| IBD | rs6974185 | A | G | 7 | -1.49E-01 | 2.15E-02 | 218828 | 3.79E-12 | 4.82E+01 |
| IBD | rs73067789 | T | C | 20 | 2.83E-01 | 5.75E-02 | 218829 | 9.08E-07 | 2.41E+01 |
| IBD | rs7781433 | A | G | 7 | 2.44E-01 | 3.79E-02 | 218830 | 1.25E-10 | 4.14E+01 |
| IBD | rs7782542 | C | T | 7 | 1.34E-01 | 2.92E-02 | 218831 | 4.08E-06 | 2.12E+01 |
| IBD | rs9467715 | C | T | 6 | 1.17E-01 | 2.29E-02 | 218832 | 3.29E-07 | 2.61E+01 |
| IBD | rs9988642 | C | T | 1 | -2.87E-01 | 4.87E-02 | 218833 | 4.11E-09 | 3.46E+01 |

SNPs, single nucleotide polymorphisms

IVs, qualified instrumental variables

AR, allergic rhinitis

IBD, inflammatory bowel disease

**Table11 SNPs selected as IVs for AR**

| **exposure** | **SNP** | **effect_allele** | **other_allele** | **chr** | **beta** | **se** | **samplesize** | **pval** | **F_statistic** |
| --- | --- | --- | --- | --- | --- | --- | --- | --- | --- |
| CD | rs10265392 | T | C | 7 | -2.32E-01 | 4.90E-02 | 212356 | 2.17E-06 | 2.25E+01 |
| CD | rs10807943 | C | T | 7 | -4.85E-01 | 6.95E-02 | 212357 | 3.16E-12 | 4.86E+01 |
| CD | rs115766821 | G | A | 2 | 4.69E-01 | 9.99E-02 | 212358 | 2.71E-06 | 2.20E+01 |
| CD | rs116929608 | G | A | 7 | 3.88E-01 | 6.31E-02 | 212359 | 7.64E-10 | 3.78E+01 |
| CD | rs117071851 | T | C | 7 | 4.57E-01 | 8.25E-02 | 212360 | 3.12E-08 | 3.06E+01 |
| CD | rs117579512 | A | G | 9 | 3.59E-01 | 7.16E-02 | 212361 | 5.22E-07 | 2.52E+01 |
| CD | rs12119179 | C | A | 1 | 1.78E-01 | 3.30E-02 | 212362 | 7.00E-08 | 2.91E+01 |
| CD | rs13194642 | T | C | 6 | 3.26E-01 | 5.89E-02 | 212363 | 3.12E-08 | 3.07E+01 |
| CD | rs139221752 | A | G | 5 | 9.53E-01 | 2.03E-01 | 212364 | 2.65E-06 | 2.21E+01 |
| CD | rs141527765 | G | A | 22 | 3.29E-01 | 6.97E-02 | 212365 | 2.36E-06 | 2.23E+01 |
| CD | rs145568234 | G | T | 6 | 1.56E+00 | 3.39E-01 | 212366 | 4.28E-06 | 2.11E+01 |
| CD | rs148265706 | A | C | 3 | 6.71E-01 | 1.45E-01 | 212367 | 3.39E-06 | 2.16E+01 |
| CD | rs181841045 | A | G | 5 | 4.20E-01 | 8.63E-02 | 212368 | 1.11E-06 | 2.37E+01 |
| CD | rs1858941 | C | T | 7 | -1.53E-01 | 3.25E-02 | 212369 | 2.45E-06 | 2.22E+01 |
| CD | rs2042154 | C | T | 5 | 2.78E-01 | 5.78E-02 | 212370 | 1.47E-06 | 2.32E+01 |
| CD | rs2274595 | G | A | 9 | 2.37E-01 | 4.83E-02 | 212371 | 9.51E-07 | 2.40E+01 |
| CD | rs2735062 | C | T | 6 | 1.80E-01 | 3.26E-02 | 212372 | 3.18E-08 | 3.06E+01 |
| CD | rs35989721 | C | T | 6 | 2.91E-01 | 5.36E-02 | 212373 | 5.86E-08 | 2.94E+01 |
| CD | rs4855881 | A | G | 3 | -1.54E-01 | 3.22E-02 | 212374 | 1.81E-06 | 2.28E+01 |
| CD | rs517958 | T | C | 11 | 1.64E-01 | 3.54E-02 | 212375 | 3.92E-06 | 2.13E+01 |
| CD | rs55885727 | T | C | 18 | 2.74E-01 | 5.80E-02 | 212376 | 2.30E-06 | 2.23E+01 |
| CD | rs57882523 | T | C | 7 | 2.32E-01 | 4.78E-02 | 212377 | 1.24E-06 | 2.35E+01 |
| CD | rs6005343 | G | A | 22 | -1.77E-01 | 3.55E-02 | 212378 | 6.70E-07 | 2.47E+01 |
| CD | rs61997160 | A | G | 7 | 2.78E-01 | 5.49E-02 | 212379 | 4.21E-07 | 2.56E+01 |
| CD | rs62443225 | A | G | 7 | 4.74E-01 | 6.32E-02 | 212380 | 6.70E-14 | 5.62E+01 |
| CD | rs6463427 | C | T | 7 | -2.40E-01 | 3.52E-02 | 212381 | 8.49E-12 | 4.66E+01 |
| CD | rs6584283 | C | T | 10 | -1.79E-01 | 3.23E-02 | 212382 | 3.38E-08 | 3.05E+01 |
| CD | rs73099728 | C | T | 5 | 2.59E-01 | 4.10E-02 | 212383 | 2.66E-10 | 3.99E+01 |
| CD | rs73434535 | A | C | 6 | 4.34E-01 | 9.41E-02 | 212384 | 3.93E-06 | 2.13E+01 |
| CD | rs78127111 | T | C | 11 | 2.45E-01 | 5.35E-02 | 212385 | 4.51E-06 | 2.10E+01 |
| CD | rs9380317 | T | C | 6 | 2.18E-01 | 4.26E-02 | 212386 | 3.03E-07 | 2.62E+01 |

SNPs, single nucleotide polymorphisms

IVs, qualified instrumental variables

AR, allergic rhinitis

CD, Crohn's disease

**Table12 SNPs selected as IVs for AR**

| **exposure** | **SNP** | **effect_allele** | **other_allele** | **chr** | **beta** | **se** | **samplesize** | **pval** | **F_statistic** |
| --- | --- | --- | --- | --- | --- | --- | --- | --- | --- |
| UC | rs10737481 | G | T | 1 | 1.57E-01 | 2.25E-02 | 214620 | 2.76E-12 | 4.89E+01 |
| UC | rs10748781 | A | C | 10 | -1.26E-01 | 2.37E-02 | 214621 | 1.18E-07 | 2.82E+01 |
| UC | rs10807943 | C | T | 7 | -3.67E-01 | 4.82E-02 | 214622 | 2.78E-14 | 5.80E+01 |
| UC | rs112266302 | A | G | 7 | 1.48E-01 | 3.14E-02 | 214623 | 2.48E-06 | 2.21E+01 |
| UC | rs11488194 | G | A | 1 | -2.59E-01 | 5.46E-02 | 214624 | 2.14E-06 | 2.25E+01 |
| UC | rs117954350 | A | G | 7 | 4.30E-01 | 7.18E-02 | 214625 | 2.02E-09 | 3.59E+01 |
| UC | rs1240695 | C | T | 1 | 1.23E-01 | 2.48E-02 | 214626 | 7.55E-07 | 2.46E+01 |
| UC | rs12685705 | G | A | 9 | 2.24E-01 | 4.52E-02 | 214627 | 7.14E-07 | 2.46E+01 |
| UC | rs145568234 | G | T | 6 | 1.32E+00 | 2.31E-01 | 214628 | 1.15E-08 | 3.26E+01 |
| UC | rs146249753 | T | C | 7 | 1.78E-01 | 3.52E-02 | 214629 | 3.95E-07 | 2.57E+01 |
| UC | rs149789095 | G | A | 6 | 3.87E-01 | 7.71E-02 | 214630 | 5.12E-07 | 2.52E+01 |
| UC | rs1892979 | G | A | 11 | 1.61E-01 | 3.48E-02 | 214631 | 3.85E-06 | 2.14E+01 |
| UC | rs2836878 | A | G | 21 | -1.32E-01 | 2.62E-02 | 214632 | 4.79E-07 | 2.54E+01 |
| UC | rs3024493 | A | C | 1 | 1.64E-01 | 3.12E-02 | 214633 | 1.34E-07 | 2.77E+01 |
| UC | rs3197999 | A | G | 3 | 1.46E-01 | 2.31E-02 | 214634 | 3.14E-10 | 3.97E+01 |
| UC | rs36051895 | T | G | 9 | 1.20E-01 | 2.38E-02 | 214635 | 4.29E-07 | 2.55E+01 |
| UC | rs3799396 | G | A | 6 | 3.07E-01 | 6.32E-02 | 214636 | 1.18E-06 | 2.36E+01 |
| UC | rs3823375 | C | T | 6 | 1.26E-01 | 2.33E-02 | 214637 | 5.86E-08 | 2.94E+01 |
| UC | rs399907 | A | G | 21 | 1.05E-01 | 2.29E-02 | 214638 | 4.73E-06 | 2.09E+01 |
| UC | rs4676410 | A | G | 2 | 1.56E-01 | 2.55E-02 | 214639 | 9.07E-10 | 3.75E+01 |
| UC | rs61997160 | A | G | 7 | 2.52E-01 | 3.86E-02 | 214640 | 7.18E-11 | 4.25E+01 |
| UC | rs62443225 | A | G | 7 | 4.03E-01 | 4.41E-02 | 214641 | 5.66E-20 | 8.35E+01 |
| UC | rs6671847 | A | G | 1 | -1.25E-01 | 2.27E-02 | 214642 | 3.26E-08 | 3.05E+01 |
| UC | rs6693413 | A | G | 1 | 1.04E-01 | 2.27E-02 | 214643 | 4.80E-06 | 2.09E+01 |
| UC | rs6974185 | A | G | 7 | -1.28E-01 | 2.44E-02 | 214644 | 1.36E-07 | 2.77E+01 |
| UC | rs75169507 | G | T | 6 | 4.12E-01 | 8.26E-02 | 214645 | 6.24E-07 | 2.48E+01 |
| UC | rs75441319 | G | A | 4 | 1.72E-01 | 3.63E-02 | 214646 | 2.07E-06 | 2.26E+01 |
| UC | rs77066761 | A | G | 17 | 5.94E-01 | 1.23E-01 | 214647 | 1.37E-06 | 2.33E+01 |
| UC | rs77683650 | A | G | 3 | -2.00E-01 | 4.16E-02 | 214648 | 1.50E-06 | 2.32E+01 |
| UC | rs9275160 | A | G | 6 | -1.75E-01 | 2.49E-02 | 214649 | 2.38E-12 | 4.91E+01 |
| UC | rs9550660 | A | G | 13 | -1.08E-01 | 2.32E-02 | 214650 | 3.34E-06 | 2.16E+01 |

SNPs, single nucleotide polymorphisms

IVs, qualified instrumental variables

AR, allergic rhinitis

UC, ulcerative colitis

**Table13 SNPs selected as IVs for Asthma**

| **exposure** | **SNP** | **effect_allele** | **other_allele** | **chr** | **beta** | **se** | **samplesize** | **pval** | **F_statistic** |
| --- | --- | --- | --- | --- | --- | --- | --- | --- | --- |
| IBD | rs1003342 | G | A | 22 | -9.50E-02 | 1.68E-02 | 34652 | 1.67E-08 | 3.20E+01 |
| IBD | rs10045431 | C | A | 5 | 1.77E-01 | 1.89E-02 | 34652 | 6.59E-21 | 8.81E+01 |
| IBD | rs10408351 | A | G | 19 | 1.38E-01 | 2.21E-02 | 34652 | 4.23E-10 | 3.89E+01 |
| IBD | rs10737481 | G | T | 1 | 1.41E-01 | 1.70E-02 | 34652 | 1.19E-16 | 6.89E+01 |
| IBD | rs10761659 | G | A | 10 | 1.62E-01 | 1.72E-02 | 34652 | 4.07E-21 | 8.86E+01 |
| IBD | rs10826797 | T | G | 10 | -1.02E-01 | 1.85E-02 | 34652 | 3.47E-08 | 3.04E+01 |
| IBD | rs11209026 | A | G | 1 | -7.26E-01 | 4.22E-02 | 34652 | 1.76E-66 | 2.96E+02 |
| IBD | rs112401990 | A | G | 2 | 1.42E-01 | 1.74E-02 | 34652 | 2.84E-16 | 6.68E+01 |
| IBD | rs112874012 | T | C | 1 | -2.50E-01 | 4.50E-02 | 34652 | 2.60E-08 | 3.10E+01 |
| IBD | rs11548656 | G | A | 16 | -2.93E-01 | 5.07E-02 | 34652 | 7.72E-09 | 3.33E+01 |
| IBD | rs11677953 | A | G | 2 | 9.76E-02 | 1.71E-02 | 34652 | 1.05E-08 | 3.26E+01 |
| IBD | rs117292830 | A | G | 6 | 4.30E-01 | 5.81E-02 | 34652 | 1.42E-13 | 5.47E+01 |
| IBD | rs11949375 | C | T | 5 | 1.89E-01 | 2.84E-02 | 34652 | 3.00E-11 | 4.42E+01 |
| IBD | rs12446550 | A | G | 16 | 1.08E-01 | 1.71E-02 | 34652 | 2.78E-10 | 3.97E+01 |
| IBD | rs1250573 | A | G | 10 | -1.14E-01 | 1.90E-02 | 34652 | 2.21E-09 | 3.57E+01 |
| IBD | rs12764283 | A | G | 10 | 1.27E-01 | 1.79E-02 | 34652 | 1.57E-12 | 5.00E+01 |
| IBD | rs140892874 | C | T | 12 | 4.10E-01 | 5.12E-02 | 34652 | 1.28E-15 | 6.40E+01 |
| IBD | rs142770866 | A | G | 19 | 2.30E-01 | 3.37E-02 | 34652 | 8.14E-12 | 4.66E+01 |
| IBD | rs145126485 | C | A | 16 | 2.83E-01 | 4.99E-02 | 34652 | 1.40E-08 | 3.22E+01 |
| IBD | rs1551399 | C | A | 8 | 1.01E-01 | 1.73E-02 | 34652 | 5.01E-09 | 3.43E+01 |
| IBD | rs1736161 | A | G | 21 | -1.23E-01 | 1.74E-02 | 34652 | 1.34E-12 | 5.02E+01 |
| IBD | rs1873625 | A | C | 3 | 1.77E-01 | 1.79E-02 | 34652 | 3.71E-23 | 9.81E+01 |
| IBD | rs1886731 | C | T | 1 | -9.71E-02 | 1.75E-02 | 34652 | 3.08E-08 | 3.08E+01 |
| IBD | rs2076756 | G | A | 16 | 1.88E-01 | 1.86E-02 | 34652 | 5.59E-24 | 1.02E+02 |
| IBD | rs2129944 | G | T | 19 | -1.19E-01 | 1.98E-02 | 34652 | 1.57E-09 | 3.63E+01 |
| IBD | rs2193041 | G | A | 12 | 1.34E-01 | 1.72E-02 | 34652 | 6.91E-15 | 6.04E+01 |
| IBD | rs2230365 | T | C | 6 | 1.54E-01 | 2.29E-02 | 34652 | 1.97E-11 | 4.51E+01 |
| IBD | rs2241878 | C | T | 2 | 1.48E-01 | 1.69E-02 | 34652 | 1.75E-18 | 7.67E+01 |
| IBD | rs2542147 | T | G | 18 | -1.51E-01 | 2.27E-02 | 34652 | 2.78E-11 | 4.44E+01 |
| IBD | rs254559 | A | C | 5 | 1.03E-01 | 1.72E-02 | 34652 | 2.08E-09 | 3.57E+01 |
| IBD | rs2836882 | A | G | 21 | -1.96E-01 | 2.01E-02 | 34652 | 1.49E-22 | 9.54E+01 |
| IBD | rs3024493 | A | C | 1 | 2.13E-01 | 2.22E-02 | 34652 | 8.48E-22 | 9.20E+01 |
| IBD | rs3091316 | A | G | 17 | -1.12E-01 | 1.91E-02 | 34652 | 3.59E-09 | 3.47E+01 |
| IBD | rs34190331 | A | G | 6 | 1.77E-01 | 3.03E-02 | 34652 | 5.39E-09 | 3.41E+01 |
| IBD | rs34920465 | G | A | 1 | -1.39E-01 | 2.26E-02 | 34652 | 8.23E-10 | 3.76E+01 |
| IBD | rs3850378 | C | T | 14 | 1.55E-01 | 2.82E-02 | 34652 | 3.80E-08 | 3.02E+01 |
| IBD | rs4077515 | T | C | 9 | 1.79E-01 | 1.72E-02 | 34652 | 1.50E-25 | 1.09E+02 |
| IBD | rs4246905 | C | T | 9 | 1.63E-01 | 1.97E-02 | 34652 | 1.42E-16 | 6.85E+01 |
| IBD | rs444210 | G | A | 6 | 1.10E-01 | 1.68E-02 | 34652 | 7.39E-11 | 4.25E+01 |
| IBD | rs45528737 | T | C | 4 | 1.67E-01 | 3.00E-02 | 34652 | 2.66E-08 | 3.09E+01 |
| IBD | rs4676410 | A | G | 2 | 1.55E-01 | 2.27E-02 | 34652 | 8.85E-12 | 4.64E+01 |
| IBD | rs4730272 | G | A | 7 | -1.34E-01 | 1.78E-02 | 34652 | 4.50E-14 | 5.68E+01 |
| IBD | rs4807569 | C | A | 19 | 1.39E-01 | 2.08E-02 | 34652 | 2.37E-11 | 4.48E+01 |
| IBD | rs56167332 | A | C | 5 | 1.56E-01 | 1.85E-02 | 34652 | 3.26E-17 | 7.15E+01 |
| IBD | rs6062496 | A | G | 20 | 1.65E-01 | 1.80E-02 | 34652 | 5.48E-20 | 8.40E+01 |
| IBD | rs6584283 | C | T | 10 | -1.80E-01 | 1.69E-02 | 34652 | 1.70E-26 | 1.14E+02 |
| IBD | rs6826501 | T | C | 4 | -9.28E-02 | 1.69E-02 | 34652 | 4.12E-08 | 3.01E+01 |
| IBD | rs6880778 | G | A | 5 | 1.88E-01 | 1.73E-02 | 34652 | 2.14E-27 | 1.18E+02 |
| IBD | rs6911490 | C | T | 6 | -1.43E-01 | 2.08E-02 | 34652 | 6.82E-12 | 4.71E+01 |
| IBD | rs72798422 | C | T | 16 | 2.78E-01 | 4.31E-02 | 34652 | 1.19E-10 | 4.15E+01 |
| IBD | rs7282490 | A | G | 21 | -1.45E-01 | 1.70E-02 | 34652 | 1.28E-17 | 7.31E+01 |
| IBD | rs7285952 | G | T | 22 | -1.76E-01 | 2.35E-02 | 34652 | 7.60E-14 | 5.61E+01 |
| IBD | rs744166 | G | A | 17 | -1.21E-01 | 1.72E-02 | 34652 | 2.16E-12 | 4.92E+01 |
| IBD | rs7523335 | A | G | 1 | -1.41E-01 | 2.25E-02 | 34652 | 4.16E-10 | 3.90E+01 |
| IBD | rs9370774 | C | T | 6 | -1.31E-01 | 2.19E-02 | 34652 | 2.54E-09 | 3.56E+01 |
| IBD | rs9934775 | T | C | 16 | -1.40E-01 | 2.32E-02 | 34652 | 1.71E-09 | 3.62E+01 |

SNPs, single nucleotide polymorphisms

IVs, qualified instrumental variables

IBD, inflammatory bowel disease

**Table14 SNPs selected as IVs for Asthma**

| **exposure** | **SNP** | **effect_allele** | **other_allele** | **chr** | **beta** | **se** | **samplesize** | **pval** | **F_statistic** |
| --- | --- | --- | --- | --- | --- | --- | --- | --- | --- |
| CD | rs10045431 | C | A | 5 | 2.16E-01 | 2.60E-02 | 20883 | 9.06E-17 | 6.89E+01 |
| CD | rs10055349 | A | G | 5 | 2.14E-01 | 2.74E-02 | 20883 | 5.63E-15 | 6.12E+01 |
| CD | rs1056441 | C | T | 20 | 1.67E-01 | 2.55E-02 | 20883 | 5.44E-11 | 4.29E+01 |
| CD | rs10748781 | A | C | 10 | -2.19E-01 | 2.38E-02 | 20883 | 3.72E-20 | 8.47E+01 |
| CD | rs10761659 | G | A | 10 | 2.12E-01 | 2.37E-02 | 20883 | 3.42E-19 | 8.00E+01 |
| CD | rs111564463 | G | A | 16 | 5.63E-01 | 9.32E-02 | 20883 | 1.51E-09 | 3.65E+01 |
| CD | rs11209026 | A | G | 1 | -9.95E-01 | 6.39E-02 | 20883 | 1.05E-54 | 2.43E+02 |
| CD | rs112401990 | A | G | 2 | 1.32E-01 | 2.37E-02 | 20883 | 2.35E-08 | 3.11E+01 |
| CD | rs114607072 | T | G | 6 | 4.42E-01 | 6.29E-02 | 20883 | 2.20E-12 | 4.93E+01 |
| CD | rs1250573 | A | G | 10 | -1.71E-01 | 2.64E-02 | 20883 | 9.01E-11 | 4.19E+01 |
| CD | rs12717899 | T | G | 5 | 1.59E-01 | 2.89E-02 | 20883 | 3.59E-08 | 3.03E+01 |
| CD | rs1297271 | T | C | 21 | -1.55E-01 | 2.37E-02 | 20883 | 6.28E-11 | 4.27E+01 |
| CD | rs13135092 | G | A | 4 | 2.21E-01 | 3.89E-02 | 20883 | 1.21E-08 | 3.24E+01 |
| CD | rs143345302 | T | C | 6 | 2.12E-01 | 3.59E-02 | 20883 | 3.44E-09 | 3.49E+01 |
| CD | rs145126485 | C | A | 16 | 6.27E-01 | 6.15E-02 | 20883 | 2.26E-24 | 1.04E+02 |
| CD | rs1456896 | T | C | 7 | 1.39E-01 | 2.51E-02 | 20883 | 2.90E-08 | 3.08E+01 |
| CD | rs151314883 | A | G | 22 | -2.24E-01 | 3.27E-02 | 20883 | 7.12E-12 | 4.69E+01 |
| CD | rs1873625 | A | C | 3 | 1.81E-01 | 2.43E-02 | 20883 | 1.09E-13 | 5.53E+01 |
| CD | rs1932990 | T | C | 13 | 1.53E-01 | 2.63E-02 | 20883 | 6.02E-09 | 3.38E+01 |
| CD | rs2076756 | G | A | 16 | 4.00E-01 | 2.42E-02 | 20883 | 3.24E-61 | 2.73E+02 |
| CD | rs2129944 | G | T | 19 | -1.56E-01 | 2.71E-02 | 20883 | 7.81E-09 | 3.32E+01 |
| CD | rs2505640 | G | A | 10 | -1.46E-01 | 2.37E-02 | 20883 | 7.61E-10 | 3.78E+01 |
| CD | rs281379 | A | G | 19 | 1.40E-01 | 2.38E-02 | 20883 | 4.26E-09 | 3.45E+01 |
| CD | rs28701841 | A | G | 6 | 2.24E-01 | 3.73E-02 | 20883 | 1.85E-09 | 3.62E+01 |
| CD | rs3024505 | A | G | 1 | 1.78E-01 | 3.02E-02 | 20883 | 3.90E-09 | 3.47E+01 |
| CD | rs3091315 | G | A | 17 | -1.80E-01 | 2.63E-02 | 20883 | 9.52E-12 | 4.66E+01 |
| CD | rs3810936 | C | T | 9 | 2.08E-01 | 2.63E-02 | 20883 | 2.46E-15 | 6.24E+01 |
| CD | rs4077515 | T | C | 9 | 2.16E-01 | 2.35E-02 | 20883 | 4.37E-20 | 8.44E+01 |
| CD | rs444210 | G | A | 6 | 1.63E-01 | 2.29E-02 | 20883 | 1.02E-12 | 5.09E+01 |
| CD | rs4486887 | T | C | 16 | -1.96E-01 | 2.47E-02 | 20883 | 2.04E-15 | 6.30E+01 |
| CD | rs4820091 | G | T | 22 | 1.72E-01 | 2.82E-02 | 20883 | 1.22E-09 | 3.71E+01 |
| CD | rs4851586 | C | T | 2 | -1.69E-01 | 2.61E-02 | 20883 | 9.94E-11 | 4.19E+01 |
| CD | rs4902642 | A | G | 14 | -1.29E-01 | 2.36E-02 | 20883 | 4.34E-08 | 3.00E+01 |
| CD | rs56167332 | A | C | 5 | 1.70E-01 | 2.55E-02 | 20883 | 2.28E-11 | 4.47E+01 |
| CD | rs6588243 | C | A | 1 | 1.32E-01 | 2.34E-02 | 20883 | 1.78E-08 | 3.17E+01 |
| CD | rs6704109 | T | C | 1 | 2.02E-01 | 2.56E-02 | 20883 | 2.77E-15 | 6.23E+01 |
| CD | rs697693 | A | G | 1 | 1.72E-01 | 2.81E-02 | 20883 | 8.36E-10 | 3.76E+01 |
| CD | rs7276302 | G | A | 21 | -1.72E-01 | 2.31E-02 | 20883 | 1.23E-13 | 5.52E+01 |
| CD | rs72798422 | C | T | 16 | 5.90E-01 | 5.08E-02 | 20883 | 3.19E-31 | 1.35E+02 |
| CD | rs7423615 | T | C | 2 | 1.64E-01 | 2.86E-02 | 20883 | 1.12E-08 | 3.27E+01 |
| CD | rs744166 | G | A | 17 | -1.29E-01 | 2.33E-02 | 20883 | 2.92E-08 | 3.08E+01 |
| CD | rs7499231 | G | A | 16 | -2.39E-01 | 4.19E-02 | 20883 | 1.16E-08 | 3.25E+01 |
| CD | rs76532080 | T | C | 16 | 3.58E-01 | 5.22E-02 | 20883 | 6.47E-12 | 4.71E+01 |
| CD | rs7713270 | T | C | 5 | 2.97E-01 | 2.41E-02 | 20883 | 6.97E-35 | 1.51E+02 |
| CD | rs80262450 | A | G | 18 | 2.83E-01 | 3.53E-02 | 20883 | 1.08E-15 | 6.43E+01 |
| CD | rs921720 | G | A | 8 | 1.63E-01 | 2.37E-02 | 20883 | 6.40E-12 | 4.72E+01 |

SNPs, single nucleotide polymorphisms

IVs, qualified instrumental variables

CD, Crohn's disease

**Table15 SNPs selected as IVs for Asthma**

| **exposure** | **SNP** | **effect_allele** | **other_allele** | **chr** | **beta** | **se** | **samplesize** | **pval** | **F_statistic** |
| --- | --- | --- | --- | --- | --- | --- | --- | --- | --- |
| UC | rs10045431 | C | A | 5 | 1.45E-01 | 2.38E-02 | 27432 | 1.09E-09 | 3.71E+01 |
| UC | rs10182512 | A | G | 2 | 1.61E-01 | 2.23E-02 | 27432 | 5.19E-13 | 5.20E+01 |
| UC | rs10272963 | T | C | 7 | -1.72E-01 | 2.16E-02 | 27432 | 1.69E-15 | 6.33E+01 |
| UC | rs10737481 | G | T | 1 | 2.50E-01 | 2.16E-02 | 27432 | 4.37E-31 | 1.34E+02 |
| UC | rs11209026 | A | G | 1 | -5.62E-01 | 5.17E-02 | 27432 | 1.58E-27 | 1.18E+02 |
| UC | rs114152040 | A | G | 5 | 3.40E-01 | 6.23E-02 | 27432 | 4.95E-08 | 2.97E+01 |
| UC | rs117292830 | A | G | 6 | 6.15E-01 | 7.03E-02 | 27432 | 2.20E-18 | 7.65E+01 |
| UC | rs12612675 | G | A | 2 | 1.23E-01 | 2.19E-02 | 27432 | 1.98E-08 | 3.15E+01 |
| UC | rs12817473 | G | A | 12 | 1.91E-01 | 2.17E-02 | 27432 | 1.71E-18 | 7.72E+01 |
| UC | rs1317209 | A | G | 1 | 1.46E-01 | 2.65E-02 | 27432 | 3.47E-08 | 3.04E+01 |
| UC | rs1359946 | A | G | 13 | 1.58E-01 | 2.69E-02 | 27432 | 3.84E-09 | 3.46E+01 |
| UC | rs137845 | G | A | 22 | 1.18E-01 | 2.12E-02 | 27432 | 2.38E-08 | 3.11E+01 |
| UC | rs1801274 | G | A | 1 | -1.83E-01 | 2.17E-02 | 27432 | 3.78E-17 | 7.10E+01 |
| UC | rs1886731 | C | T | 1 | -1.41E-01 | 2.21E-02 | 27432 | 2.25E-10 | 4.04E+01 |
| UC | rs2301989 | A | G | 7 | -1.41E-01 | 2.17E-02 | 27432 | 8.55E-11 | 4.22E+01 |
| UC | rs254559 | A | C | 5 | 1.24E-01 | 2.15E-02 | 27432 | 7.63E-09 | 3.34E+01 |
| UC | rs3024493 | A | C | 1 | 2.36E-01 | 2.76E-02 | 27432 | 1.09E-17 | 7.33E+01 |
| UC | rs34920465 | G | A | 1 | -1.93E-01 | 2.90E-02 | 27432 | 2.93E-11 | 4.43E+01 |
| UC | rs3829111 | A | G | 9 | 1.56E-01 | 2.14E-02 | 27432 | 2.89E-13 | 5.33E+01 |
| UC | rs45627734 | A | G | 6 | 4.06E-01 | 6.34E-02 | 27432 | 1.47E-10 | 4.10E+01 |
| UC | rs4574921 | T | C | 9 | 1.51E-01 | 2.56E-02 | 27432 | 4.24E-09 | 3.46E+01 |
| UC | rs4676410 | A | G | 2 | 2.08E-01 | 2.84E-02 | 27432 | 2.46E-13 | 5.35E+01 |
| UC | rs483905 | A | G | 11 | 1.29E-01 | 2.28E-02 | 27432 | 1.57E-08 | 3.20E+01 |
| UC | rs56167332 | A | C | 5 | 1.52E-01 | 2.31E-02 | 27432 | 5.30E-11 | 4.31E+01 |
| UC | rs6017342 | C | A | 20 | 1.91E-01 | 2.40E-02 | 27432 | 1.38E-15 | 6.35E+01 |
| UC | rs6062496 | A | G | 20 | 1.58E-01 | 2.24E-02 | 27432 | 1.47E-12 | 5.01E+01 |
| UC | rs6933404 | C | T | 6 | 1.67E-01 | 2.52E-02 | 27432 | 3.68E-11 | 4.38E+01 |
| UC | rs7282490 | A | G | 21 | -1.40E-01 | 2.14E-02 | 27432 | 7.08E-11 | 4.26E+01 |
| UC | rs7523335 | A | G | 1 | -1.70E-01 | 2.85E-02 | 27432 | 2.29E-09 | 3.57E+01 |
| UC | rs7752873 | T | C | 6 | 1.82E-01 | 3.03E-02 | 27432 | 1.83E-09 | 3.62E+01 |
| UC | rs7911680 | C | A | 10 | -1.72E-01 | 2.13E-02 | 27432 | 8.27E-16 | 6.51E+01 |
| UC | rs798502 | C | A | 7 | -1.36E-01 | 2.39E-02 | 27432 | 1.21E-08 | 3.26E+01 |
| UC | rs9977672 | A | G | 21 | -2.45E-01 | 2.61E-02 | 27432 | 6.21E-21 | 8.81E+01 |

SNPs, single nucleotide polymorphisms

IVs, qualified instrumental variables

UC, ulcerative colitis

**Table16 SNPs selected as IVs for AD**

| **exposure** | **SNP** | **effect_allele** | **other_allele** | **chr** | **beta** | **se** | **samplesize** | **pval** | **F_statistic** |
| --- | --- | --- | --- | --- | --- | --- | --- | --- | --- |
| IBD | rs10246518 | A | G | 7 | 1.57E-01 | 2.95E-02 | 218792 | 1.06E-07 | 2.83E+01 |
| IBD | rs10737481 | G | T | 1 | 1.23E-01 | 1.99E-02 | 218793 | 6.20E-10 | 3.81E+01 |
| IBD | rs10748781 | A | C | 10 | -1.33E-01 | 2.09E-02 | 218794 | 1.97E-10 | 4.07E+01 |
| IBD | rs10761659 | G | A | 10 | 9.63E-02 | 1.99E-02 | 218795 | 1.26E-06 | 2.34E+01 |
| IBD | rs10807943 | C | T | 7 | -3.67E-01 | 4.25E-02 | 218796 | 6.20E-18 | 7.44E+01 |
| IBD | rs11204894 | T | G | 1 | -1.22E-01 | 2.23E-02 | 218797 | 5.22E-08 | 2.97E+01 |
| IBD | rs112266302 | A | G | 7 | 1.84E-01 | 2.78E-02 | 218798 | 3.23E-11 | 4.39E+01 |
| IBD | rs11244204 | G | A | 9 | -1.93E-01 | 4.11E-02 | 218799 | 2.56E-06 | 2.21E+01 |
| IBD | rs11583398 | A | C | 1 | 1.37E-01 | 2.83E-02 | 218800 | 1.45E-06 | 2.33E+01 |
| IBD | rs11658993 | T | C | 17 | 1.08E-01 | 1.99E-02 | 218801 | 6.53E-08 | 2.93E+01 |
| IBD | rs116929608 | G | A | 7 | 2.71E-01 | 3.85E-02 | 218802 | 2.08E-12 | 4.94E+01 |
| IBD | rs117245455 | G | T | 17 | 3.50E-01 | 7.64E-02 | 218803 | 4.47E-06 | 2.10E+01 |
| IBD | rs117959250 | T | G | 7 | 2.46E-01 | 4.92E-02 | 218804 | 5.67E-07 | 2.50E+01 |
| IBD | rs12175489 | A | G | 6 | 1.45E-01 | 2.76E-02 | 218805 | 1.57E-07 | 2.75E+01 |
| IBD | rs12581964 | T | C | 12 | 2.46E-01 | 5.37E-02 | 218806 | 4.59E-06 | 2.10E+01 |
| IBD | rs13300483 | T | C | 9 | 9.82E-02 | 2.12E-02 | 218807 | 3.56E-06 | 2.15E+01 |
| IBD | rs139348065 | A | G | 1 | 5.60E-01 | 1.17E-01 | 218808 | 1.67E-06 | 2.29E+01 |
| IBD | rs139613239 | G | A | 10 | -1.75E-01 | 3.72E-02 | 218809 | 2.72E-06 | 2.20E+01 |
| IBD | rs145568234 | G | T | 6 | 1.31E+00 | 2.03E-01 | 218810 | 1.10E-10 | 4.16E+01 |
| IBD | rs146249753 | T | C | 7 | 1.74E-01 | 3.11E-02 | 218811 | 2.26E-08 | 3.12E+01 |
| IBD | rs149368937 | G | A | 5 | 1.38E-01 | 2.68E-02 | 218812 | 2.48E-07 | 2.66E+01 |
| IBD | rs17689550 | T | C | 5 | -1.34E-01 | 2.45E-02 | 218813 | 4.68E-08 | 2.99E+01 |
| IBD | rs1801274 | G | A | 1 | -1.06E-01 | 1.98E-02 | 218814 | 8.89E-08 | 2.87E+01 |
| IBD | rs2149560 | T | G | 9 | 1.10E-01 | 2.00E-02 | 218815 | 3.63E-08 | 3.02E+01 |
| IBD | rs2480428 | G | A | 13 | -9.53E-02 | 2.03E-02 | 218816 | 2.63E-06 | 2.20E+01 |
| IBD | rs2836878 | A | G | 21 | -1.19E-01 | 2.31E-02 | 218817 | 2.96E-07 | 2.64E+01 |
| IBD | rs3197999 | A | G | 3 | 1.31E-01 | 2.04E-02 | 218818 | 1.38E-10 | 4.12E+01 |
| IBD | rs3799396 | G | A | 6 | 2.55E-01 | 5.55E-02 | 218819 | 4.42E-06 | 2.11E+01 |
| IBD | rs3823375 | C | T | 6 | 1.21E-01 | 2.05E-02 | 218820 | 4.30E-09 | 3.46E+01 |
| IBD | rs4259333 | T | C | 7 | -1.10E-01 | 2.38E-02 | 218821 | 3.96E-06 | 2.12E+01 |
| IBD | rs4676410 | A | G | 2 | 1.41E-01 | 2.25E-02 | 218822 | 3.16E-10 | 3.95E+01 |
| IBD | rs555332480 | T | G | 10 | 2.06E-01 | 4.18E-02 | 218823 | 8.27E-07 | 2.43E+01 |
| IBD | rs56116661 | T | C | 3 | -1.22E-01 | 2.39E-02 | 218824 | 3.66E-07 | 2.59E+01 |
| IBD | rs56343836 | A | G | 13 | 1.99E+00 | 4.12E-01 | 218825 | 1.37E-06 | 2.33E+01 |
| IBD | rs6062496 | A | G | 20 | 1.05E-01 | 2.08E-02 | 218826 | 5.16E-07 | 2.53E+01 |
| IBD | rs61997160 | A | G | 7 | 2.42E-01 | 3.40E-02 | 218827 | 1.10E-12 | 5.08E+01 |
| IBD | rs62443225 | A | G | 7 | 3.84E-01 | 3.87E-02 | 218828 | 3.37E-23 | 9.84E+01 |
| IBD | rs6696981 | T | G | 1 | -1.84E-01 | 3.77E-02 | 218829 | 1.09E-06 | 2.37E+01 |
| IBD | rs6966158 | A | C | 7 | -1.78E-01 | 3.42E-02 | 218830 | 1.94E-07 | 2.71E+01 |
| IBD | rs6974185 | A | G | 7 | -1.49E-01 | 2.15E-02 | 218831 | 3.79E-12 | 4.82E+01 |
| IBD | rs73067789 | T | C | 20 | 2.83E-01 | 5.75E-02 | 218832 | 9.08E-07 | 2.41E+01 |
| IBD | rs7781433 | A | G | 7 | 2.44E-01 | 3.79E-02 | 218833 | 1.25E-10 | 4.14E+01 |
| IBD | rs7782542 | C | T | 7 | 1.34E-01 | 2.92E-02 | 218834 | 4.08E-06 | 2.12E+01 |
| IBD | rs9467715 | C | T | 6 | 1.17E-01 | 2.29E-02 | 218835 | 3.29E-07 | 2.61E+01 |
| IBD | rs9988642 | C | T | 1 | -2.87E-01 | 4.87E-02 | 218836 | 4.11E-09 | 3.46E+01 |

SNPs, single nucleotide polymorphisms

IVs, qualified instrumental variables

AD, atopic dermatitis

IBD, inflammatory bowel disease

**Table17 SNPs selected as IVs for AD**

| **exposure** | **SNP** | **effect_allele** | **other_allele** | **chr** | **beta** | **se** | **samplesize** | **pval** | **F_statistic** |
| --- | --- | --- | --- | --- | --- | --- | --- | --- | --- |
| CD | rs10265392 | T | C | 7 | -2.32E-01 | 4.90E-02 | 212356 | 2.17E-06 | 2.25E+01 |
| CD | rs10807943 | C | T | 7 | -4.85E-01 | 6.95E-02 | 212357 | 3.16E-12 | 4.86E+01 |
| CD | rs112456327 | T | C | 16 | 3.25E-01 | 6.21E-02 | 212358 | 1.60E-07 | 2.74E+01 |
| CD | rs115766821 | G | A | 2 | 4.69E-01 | 9.99E-02 | 212359 | 2.71E-06 | 2.20E+01 |
| CD | rs116929608 | G | A | 7 | 3.88E-01 | 6.31E-02 | 212360 | 7.64E-10 | 3.78E+01 |
| CD | rs117071851 | T | C | 7 | 4.57E-01 | 8.25E-02 | 212361 | 3.12E-08 | 3.06E+01 |
| CD | rs117579512 | A | G | 9 | 3.59E-01 | 7.16E-02 | 212362 | 5.22E-07 | 2.52E+01 |
| CD | rs12119179 | C | A | 1 | 1.78E-01 | 3.30E-02 | 212363 | 7.00E-08 | 2.91E+01 |
| CD | rs13194642 | T | C | 6 | 3.26E-01 | 5.89E-02 | 212364 | 3.12E-08 | 3.07E+01 |
| CD | rs139221752 | A | G | 5 | 9.53E-01 | 2.03E-01 | 212365 | 2.65E-06 | 2.21E+01 |
| CD | rs141527765 | G | A | 22 | 3.29E-01 | 6.97E-02 | 212366 | 2.36E-06 | 2.23E+01 |
| CD | rs145568234 | G | T | 6 | 1.56E+00 | 3.39E-01 | 212367 | 4.28E-06 | 2.11E+01 |
| CD | rs148265706 | A | C | 3 | 6.71E-01 | 1.45E-01 | 212368 | 3.39E-06 | 2.16E+01 |
| CD | rs181841045 | A | G | 5 | 4.20E-01 | 8.63E-02 | 212369 | 1.11E-06 | 2.37E+01 |
| CD | rs1858941 | C | T | 7 | -1.53E-01 | 3.25E-02 | 212370 | 2.45E-06 | 2.22E+01 |
| CD | rs2042154 | C | T | 5 | 2.78E-01 | 5.78E-02 | 212371 | 1.47E-06 | 2.32E+01 |
| CD | rs2274595 | G | A | 9 | 2.37E-01 | 4.83E-02 | 212372 | 9.51E-07 | 2.40E+01 |
| CD | rs2735062 | C | T | 6 | 1.80E-01 | 3.26E-02 | 212373 | 3.18E-08 | 3.06E+01 |
| CD | rs35989721 | C | T | 6 | 2.91E-01 | 5.36E-02 | 212374 | 5.86E-08 | 2.94E+01 |
| CD | rs4855881 | A | G | 3 | -1.54E-01 | 3.22E-02 | 212375 | 1.81E-06 | 2.28E+01 |
| CD | rs517958 | T | C | 11 | 1.64E-01 | 3.54E-02 | 212376 | 3.92E-06 | 2.13E+01 |
| CD | rs55885727 | T | C | 18 | 2.74E-01 | 5.80E-02 | 212377 | 2.30E-06 | 2.23E+01 |
| CD | rs55904328 | A | G | 11 | 1.73E-01 | 3.44E-02 | 212378 | 5.09E-07 | 2.52E+01 |
| CD | rs57882523 | T | C | 7 | 2.32E-01 | 4.78E-02 | 212379 | 1.24E-06 | 2.35E+01 |
| CD | rs6005343 | G | A | 22 | -1.77E-01 | 3.55E-02 | 212380 | 6.70E-07 | 2.47E+01 |
| CD | rs61997160 | A | G | 7 | 2.78E-01 | 5.49E-02 | 212381 | 4.21E-07 | 2.56E+01 |
| CD | rs62443225 | A | G | 7 | 4.74E-01 | 6.32E-02 | 212382 | 6.70E-14 | 5.62E+01 |
| CD | rs6463427 | C | T | 7 | -2.40E-01 | 3.52E-02 | 212383 | 8.49E-12 | 4.66E+01 |
| CD | rs6584283 | C | T | 10 | -1.79E-01 | 3.23E-02 | 212384 | 3.38E-08 | 3.05E+01 |
| CD | rs73099728 | C | T | 5 | 2.59E-01 | 4.10E-02 | 212385 | 2.66E-10 | 3.99E+01 |
| CD | rs73434535 | A | C | 6 | 4.34E-01 | 9.41E-02 | 212386 | 3.93E-06 | 2.13E+01 |
| CD | rs78127111 | T | C | 11 | 2.45E-01 | 5.35E-02 | 212387 | 4.51E-06 | 2.10E+01 |
| CD | rs9380317 | T | C | 6 | 2.18E-01 | 4.26E-02 | 212388 | 3.03E-07 | 2.62E+01 |

SNPs, single nucleotide polymorphisms

IVs, qualified instrumental variables

AD, atopic dermatitis

CD, Crohn's disease

**Table18 SNPs selected as IVs for AD**

| **exposure** | **SNP** | **effect_allele** | **other_allele** | **chr** | **beta** | **se** | **samplesize** | **pval** | **F_statistic** |
| --- | --- | --- | --- | --- | --- | --- | --- | --- | --- |
| UC | rs10737481 | G | T | 1 | 1.57E-01 | 2.25E-02 | 214620 | 2.76E-12 | 4.89E+01 |
| UC | rs10748781 | A | C | 10 | -1.26E-01 | 2.37E-02 | 214621 | 1.18E-07 | 2.82E+01 |
| UC | rs10807943 | C | T | 7 | -3.67E-01 | 4.82E-02 | 214622 | 2.78E-14 | 5.80E+01 |
| UC | rs112266302 | A | G | 7 | 1.48E-01 | 3.14E-02 | 214623 | 2.48E-06 | 2.21E+01 |
| UC | rs11488194 | G | A | 1 | -2.59E-01 | 5.46E-02 | 214624 | 2.14E-06 | 2.25E+01 |
| UC | rs117954350 | A | G | 7 | 4.30E-01 | 7.18E-02 | 214625 | 2.02E-09 | 3.59E+01 |
| UC | rs1240695 | C | T | 1 | 1.23E-01 | 2.48E-02 | 214626 | 7.55E-07 | 2.46E+01 |
| UC | rs12685705 | G | A | 9 | 2.24E-01 | 4.52E-02 | 214627 | 7.14E-07 | 2.46E+01 |
| UC | rs12946510 | T | C | 17 | 1.27E-01 | 2.26E-02 | 214628 | 1.73E-08 | 3.17E+01 |
| UC | rs139613239 | G | A | 10 | -2.15E-01 | 4.23E-02 | 214629 | 3.67E-07 | 2.59E+01 |
| UC | rs145568234 | G | T | 6 | 1.32E+00 | 2.31E-01 | 214630 | 1.15E-08 | 3.26E+01 |
| UC | rs146249753 | T | C | 7 | 1.78E-01 | 3.52E-02 | 214631 | 3.95E-07 | 2.57E+01 |
| UC | rs149789095 | G | A | 6 | 3.87E-01 | 7.71E-02 | 214632 | 5.12E-07 | 2.52E+01 |
| UC | rs1892979 | G | A | 11 | 1.61E-01 | 3.48E-02 | 214633 | 3.85E-06 | 2.14E+01 |
| UC | rs2836878 | A | G | 21 | -1.32E-01 | 2.62E-02 | 214634 | 4.79E-07 | 2.54E+01 |
| UC | rs3024493 | A | C | 1 | 1.64E-01 | 3.12E-02 | 214635 | 1.34E-07 | 2.77E+01 |
| UC | rs3197999 | A | G | 3 | 1.46E-01 | 2.31E-02 | 214636 | 3.14E-10 | 3.97E+01 |
| UC | rs36051895 | T | G | 9 | 1.20E-01 | 2.38E-02 | 214637 | 4.29E-07 | 2.55E+01 |
| UC | rs3799396 | G | A | 6 | 3.07E-01 | 6.32E-02 | 214638 | 1.18E-06 | 2.36E+01 |
| UC | rs3823375 | C | T | 6 | 1.26E-01 | 2.33E-02 | 214639 | 5.86E-08 | 2.94E+01 |
| UC | rs399907 | A | G | 21 | 1.05E-01 | 2.29E-02 | 214640 | 4.73E-06 | 2.09E+01 |
| UC | rs4676410 | A | G | 2 | 1.56E-01 | 2.55E-02 | 214641 | 9.07E-10 | 3.75E+01 |
| UC | rs555332480 | T | G | 10 | 2.33E-01 | 4.74E-02 | 214642 | 8.67E-07 | 2.42E+01 |
| UC | rs61997160 | A | G | 7 | 2.52E-01 | 3.86E-02 | 214643 | 7.18E-11 | 4.25E+01 |
| UC | rs62443225 | A | G | 7 | 4.03E-01 | 4.41E-02 | 214644 | 5.66E-20 | 8.35E+01 |
| UC | rs6671847 | A | G | 1 | -1.25E-01 | 2.27E-02 | 214645 | 3.26E-08 | 3.05E+01 |
| UC | rs6693413 | A | G | 1 | 1.04E-01 | 2.27E-02 | 214646 | 4.80E-06 | 2.09E+01 |
| UC | rs6974185 | A | G | 7 | -1.28E-01 | 2.44E-02 | 214647 | 1.36E-07 | 2.77E+01 |
| UC | rs75169507 | G | T | 6 | 4.12E-01 | 8.26E-02 | 214648 | 6.24E-07 | 2.48E+01 |
| UC | rs75441319 | G | A | 4 | 1.72E-01 | 3.63E-02 | 214649 | 2.07E-06 | 2.26E+01 |
| UC | rs77066761 | A | G | 17 | 5.94E-01 | 1.23E-01 | 214650 | 1.37E-06 | 2.33E+01 |
| UC | rs77683650 | A | G | 3 | -2.00E-01 | 4.16E-02 | 214651 | 1.50E-06 | 2.32E+01 |
| UC | rs9275160 | A | G | 6 | -1.75E-01 | 2.49E-02 | 214652 | 2.38E-12 | 4.91E+01 |
| UC | rs9550660 | A | G | 13 | -1.08E-01 | 2.32E-02 | 214653 | 3.34E-06 | 2.16E+01 |

SNPs, single nucleotide polymorphisms

IVs, qualified instrumental variables

AD, atopic dermatitis

UC, ulcerative colitis
