# Supplementary material for: Atopic disease and inflammatory bowel disease: A bidirectional Mendelian randomization study
Source: Medicine (Baltimore). 2024 Oct 18;103(42):e40143. doi: 10.1097/MD.0000000000040143 (PMC11495711; doi:10.1097/MD.0000000000040143)
Supplement: Supplementary file 2 [file medi-103-e40143-s002.docx]

**Supplementary material**


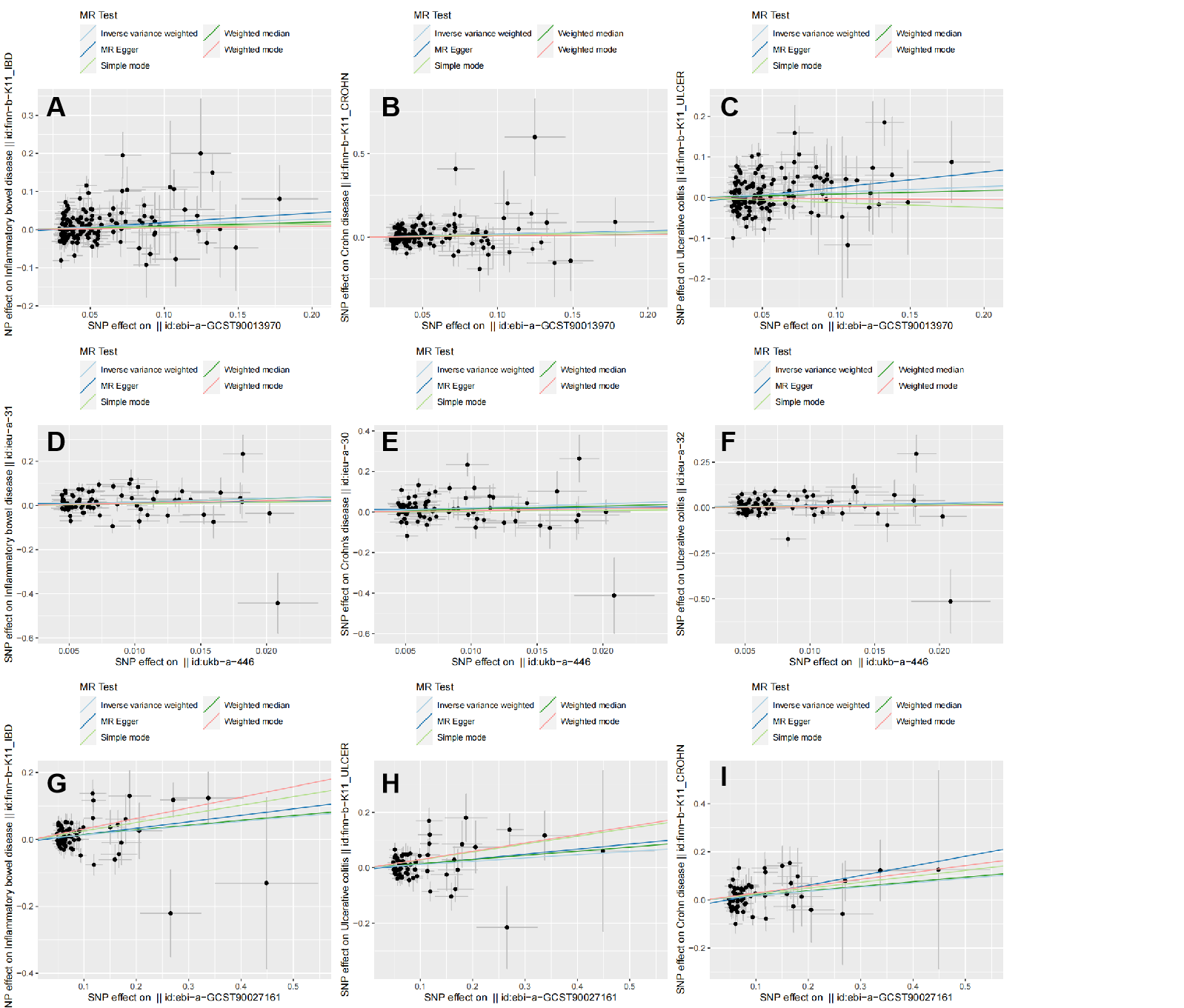


Supplementary Figure 1. Scatter plots

The estimate of intercept can be interpreted as an estimate of the average pleiotropy of all single-nucleotide polymorphisms (SNPs), and the slope coefficient provides an estimate of the bias of the causal effect. (A) AR on IBD. (B) AR on CD. (C) AR on UC. (D) Asthma on IBD. (E) Asthma on CD. (F) Asthma on UC. (G) AD on IBD. (H) AD on CD. (I) AD on UC.

**Supplementary material**


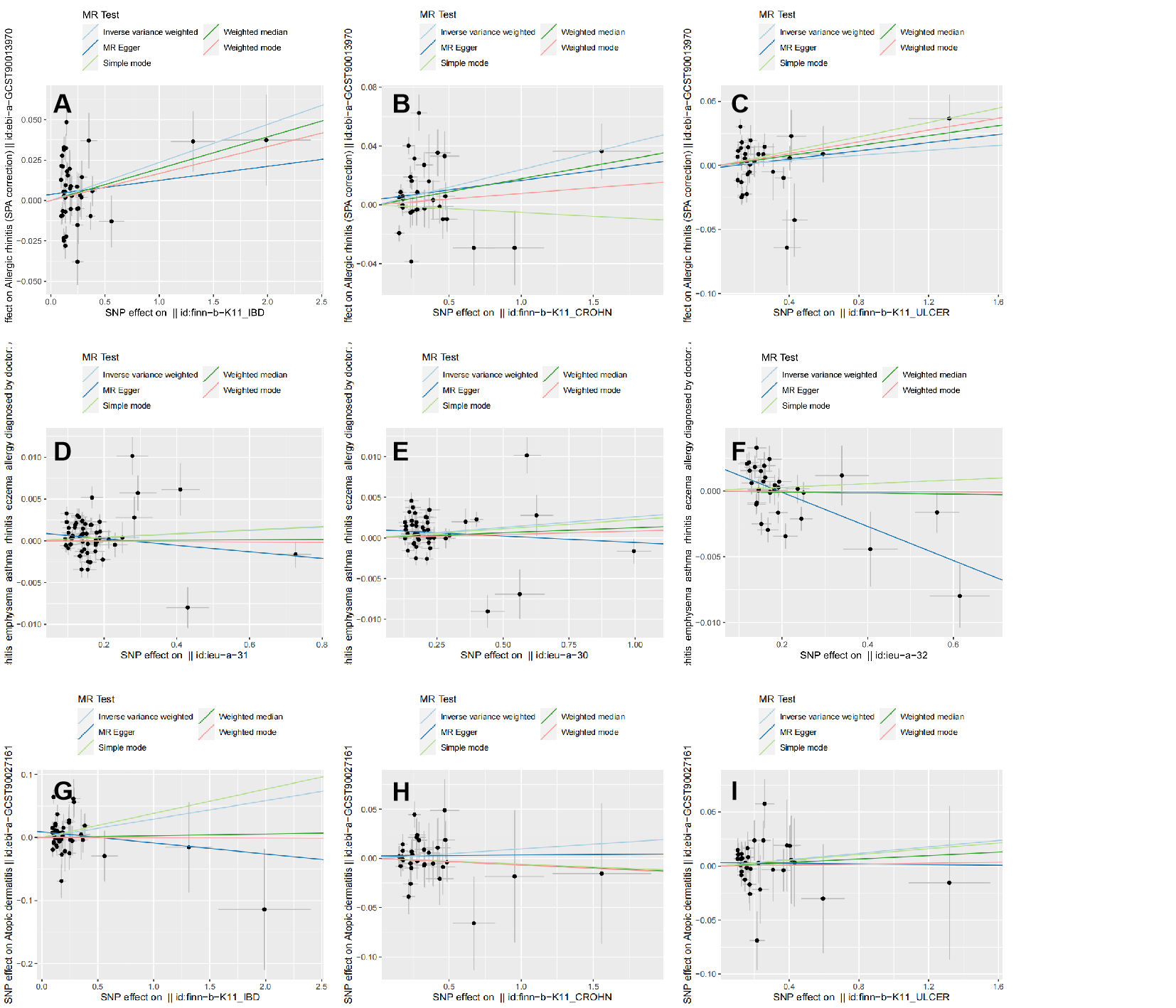


Supplementary Figure 2. Scatter plots

(A) IBD on AR. (B) CD on AR. (C) UC on AR. (D) IBD on Asthma. (E) CD on Asthma. (F) UC on Asthma. (G) IBD on AD. (H) CD on AD. (I) UC on AD.

**Supplementary material**


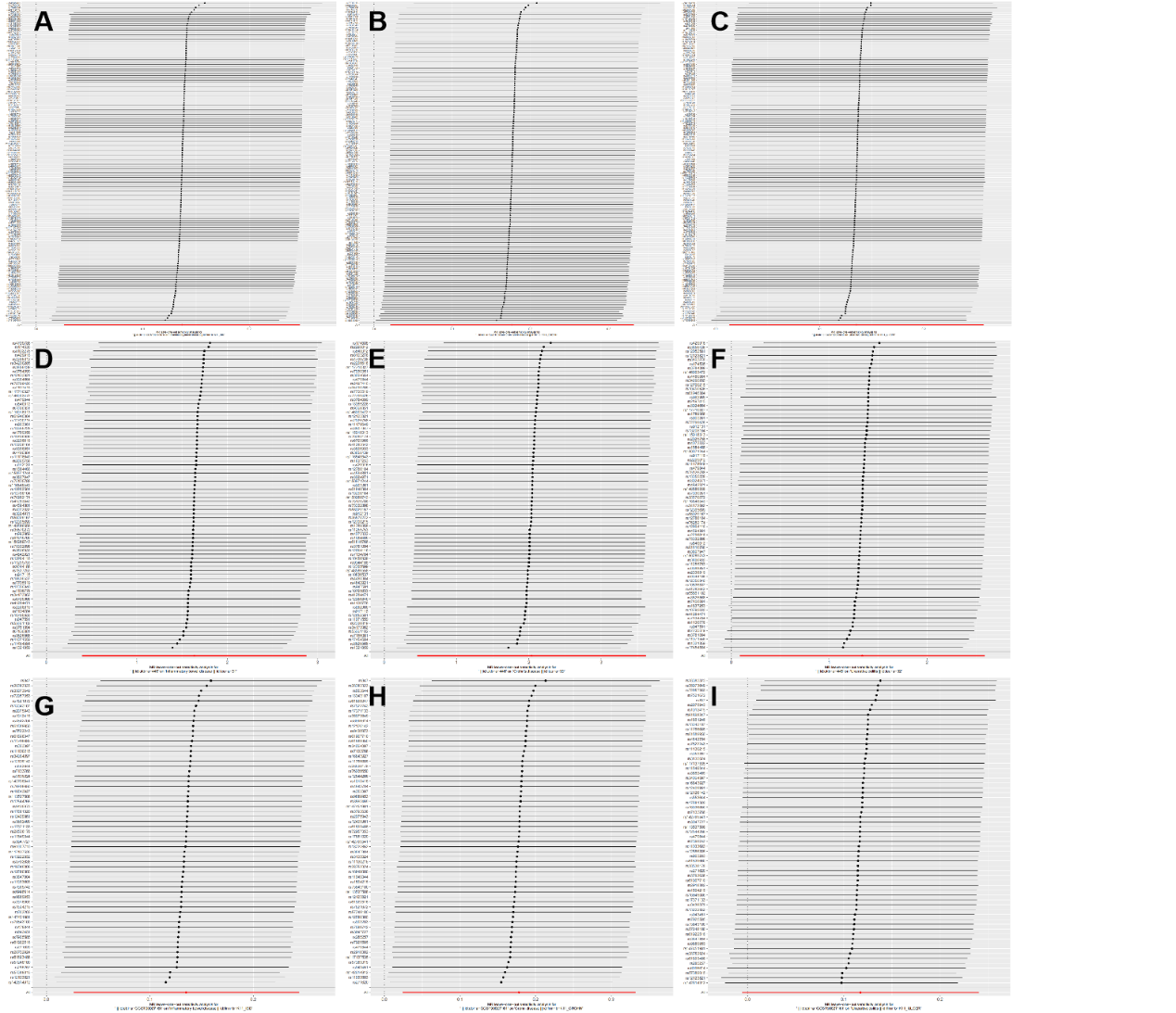


Supplementary Figure 3. Leave-one-out sensitivity analysis

(A) AR on IBD. (B) AR on CD. (C) AR on UC. (D) Asthma on IBD. (E) Asthma on CD. (F) Asthma on UC. (G) AD on IBD. (H) AD on CD. (I) AD on UC.

**Supplementary material**


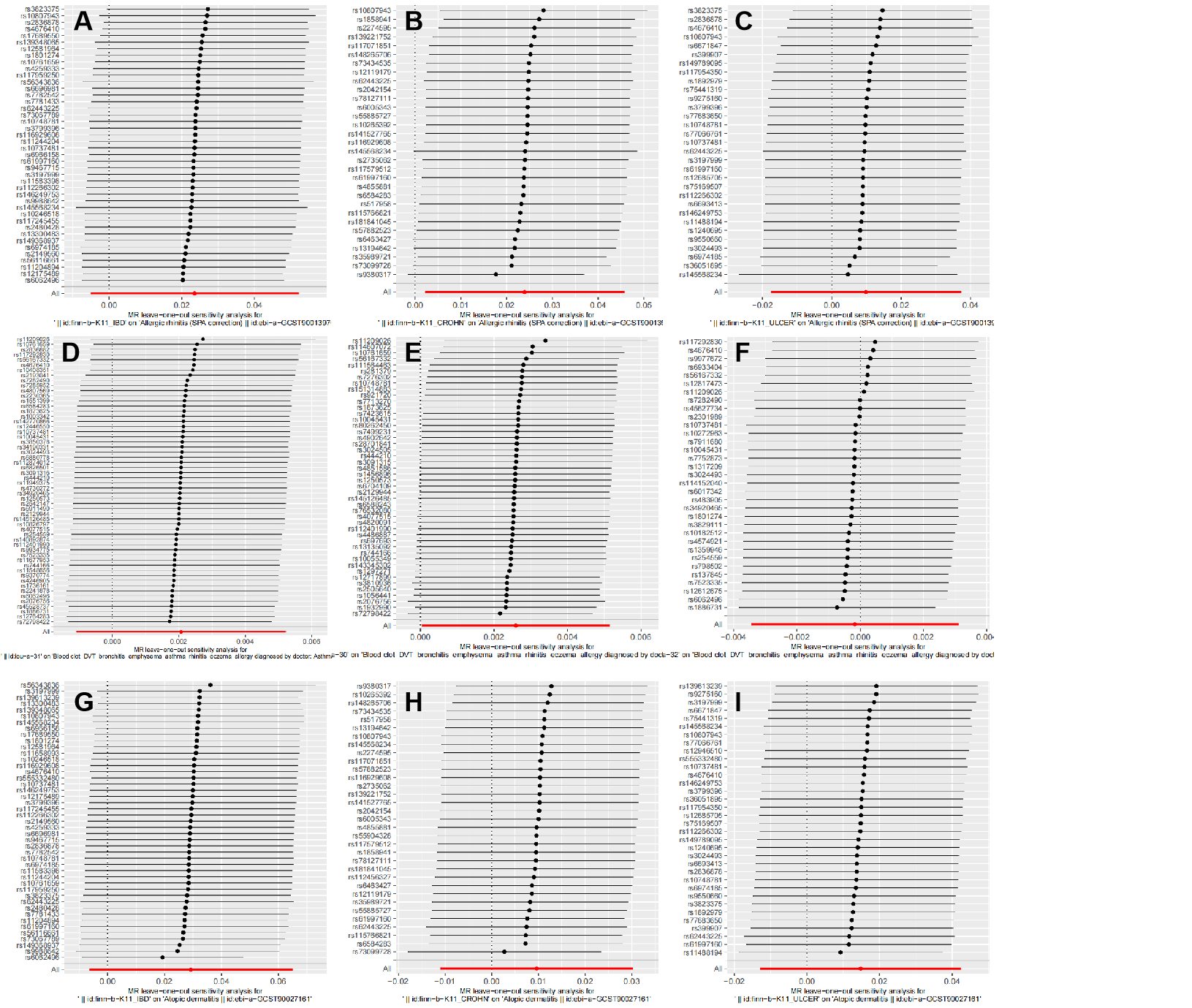


Supplementary Figure 4. Leave-one-out sensitivity analysis

(A) IBD on AR. (B) CD on AR. (C) UC on AR. (D) IBD on Asthma. (E) CD on Asthma. (F) UC on Asthma. (G) IBD on AD. (H) CD on AD. (I) UC on AD.

**Supplementary material**


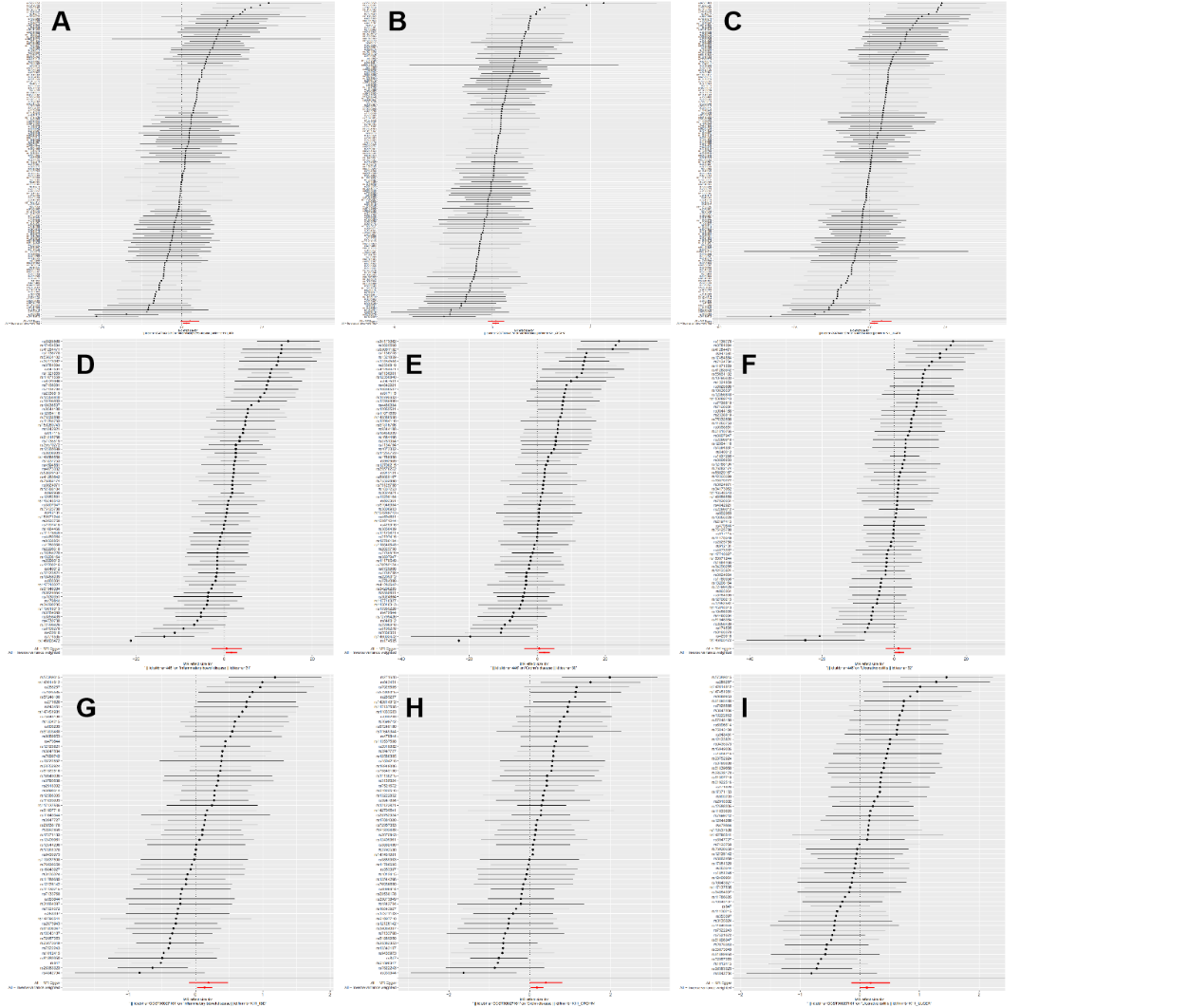


Supplementary Figure 5. Forest plots

(A) AR on IBD. (B) AR on CD. (C) AR on UC. (D) Asthma on IBD. (E) Asthma on CD. (F) Asthma on UC. (G) AD on IBD. (H) AD on CD. (I) AD on UC.

**Supplementary material**


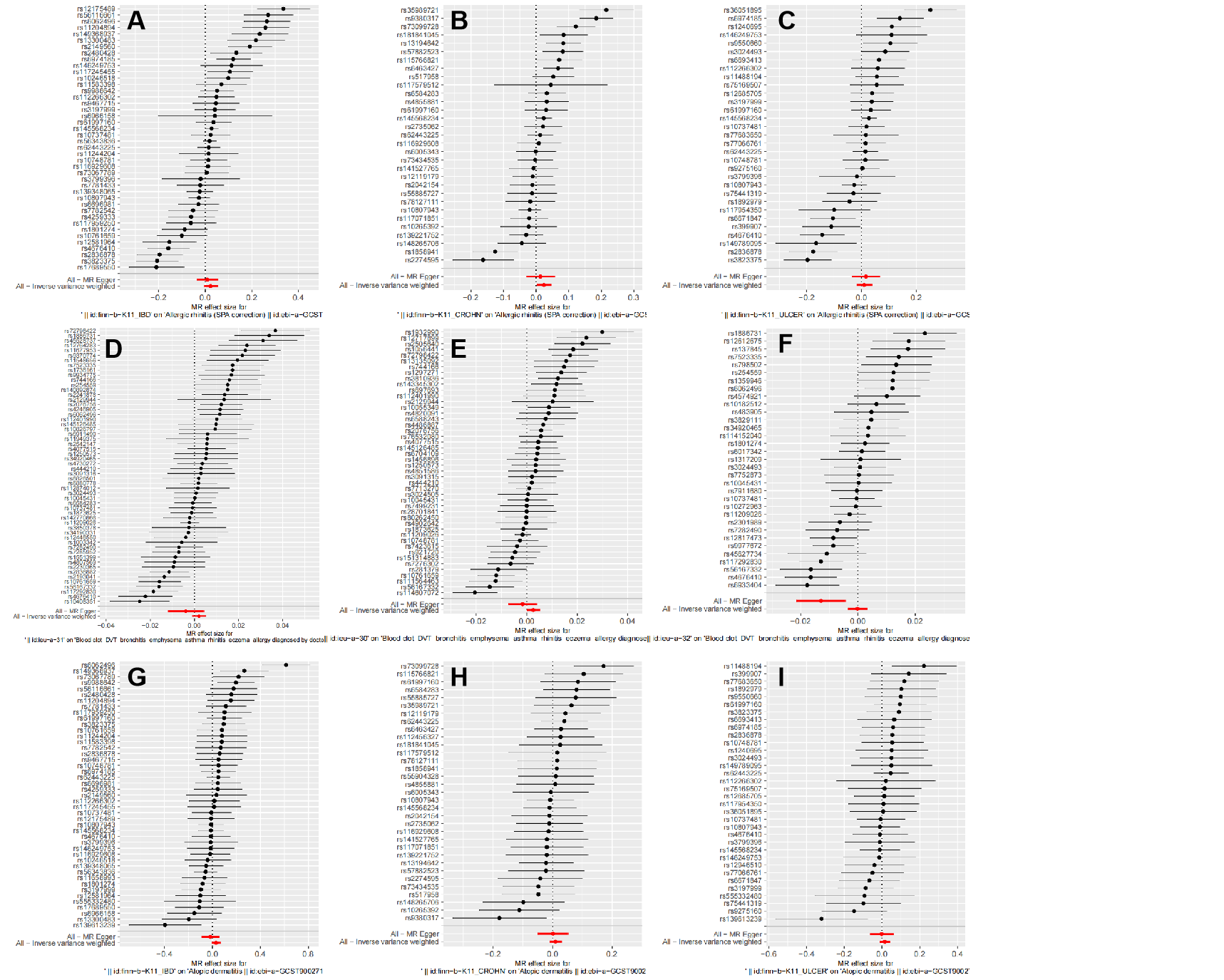


Supplementary Figure 6. Forest plots

(A) IBD on AR. (B) CD on AR. (C) UC on AR. (D) IBD on Asthma. (E) CD on Asthma. (F) UC on Asthma. (G) IBD on AD. (H) CD on AD. (I) UC on AD.

**Supplementary material**


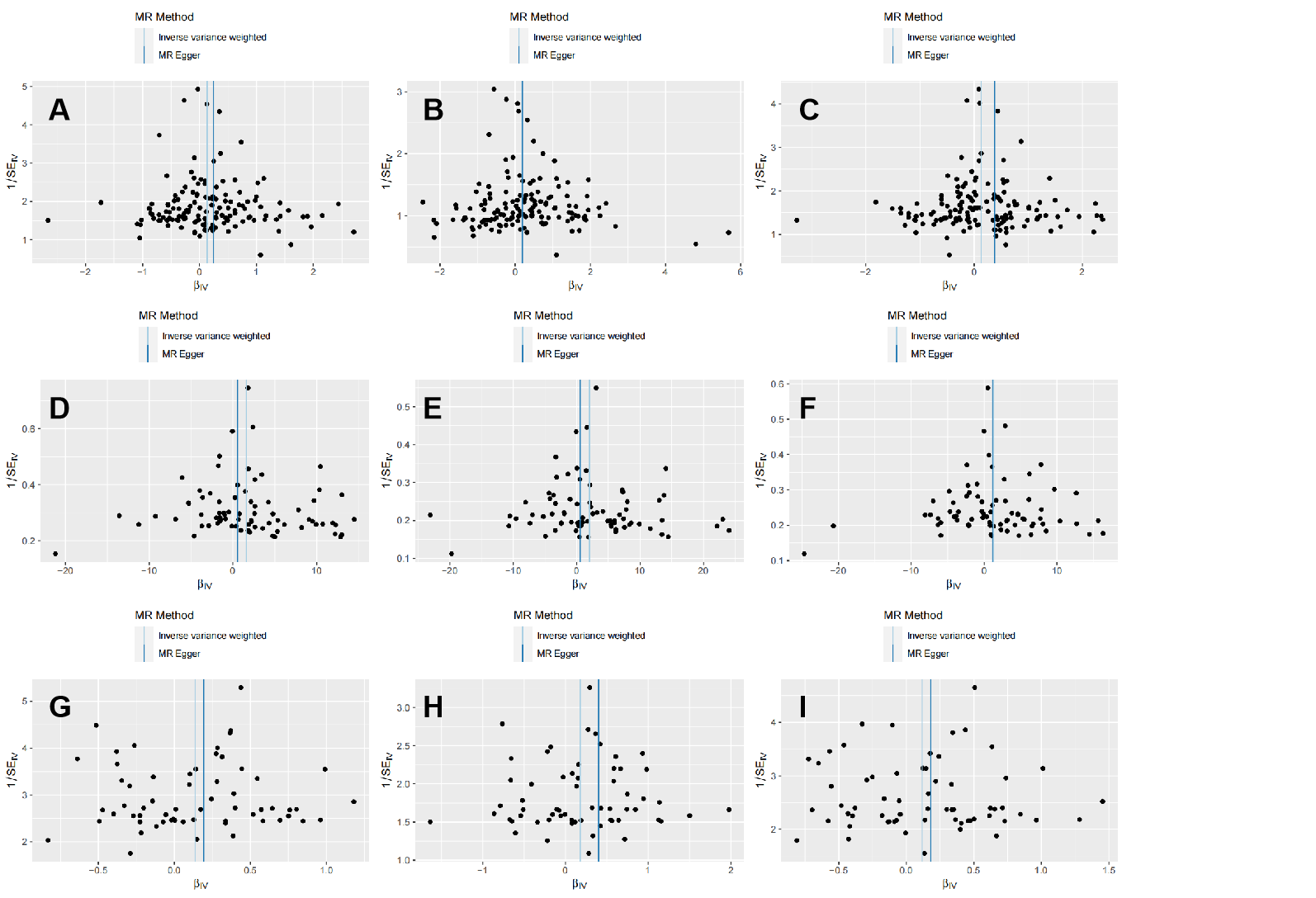


Supplementary Figure 7. Funnel plots

The x-axis represents β, and the y-axis represents 1/SE (standard error). (A) AR on IBD. (B) AR on CD. (C) AR on UC. (D) Asthma on IBD. (E) Asthma on CD. (F) Asthma on UC. (G) AD on IBD. (H) AD on CD. (I) AD on UC.

**Supplementary material**


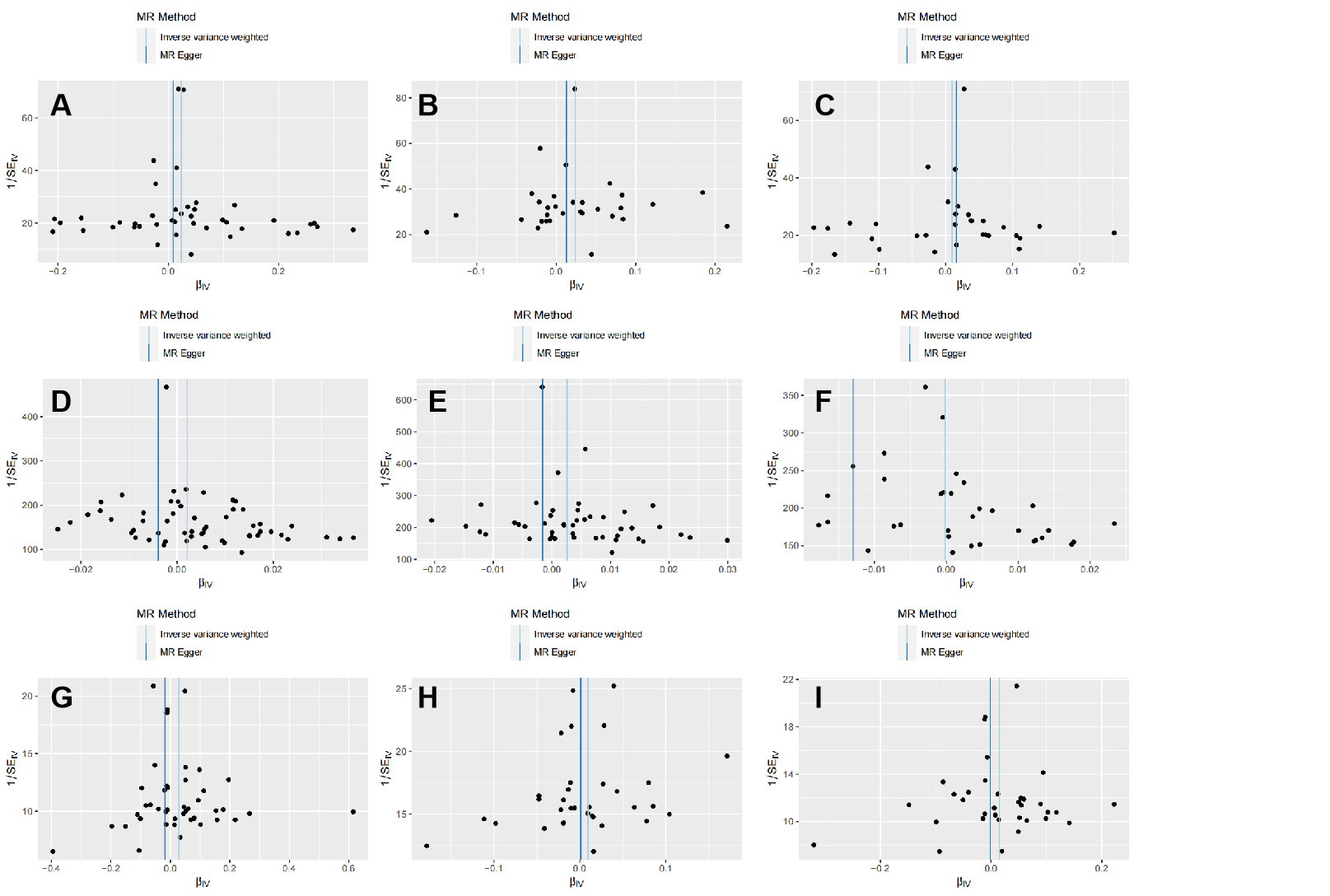


Supplementary Figure 8. Funnel plots

(A) IBD on AR. (B) CD on AR. (C) UC on AR. (D) IBD on Asthma. (E) CD on Asthma. (F) UC on Asthma. (G) IBD on AD. (H) CD on AD. (I) UC on AD.

**Supplementary material**


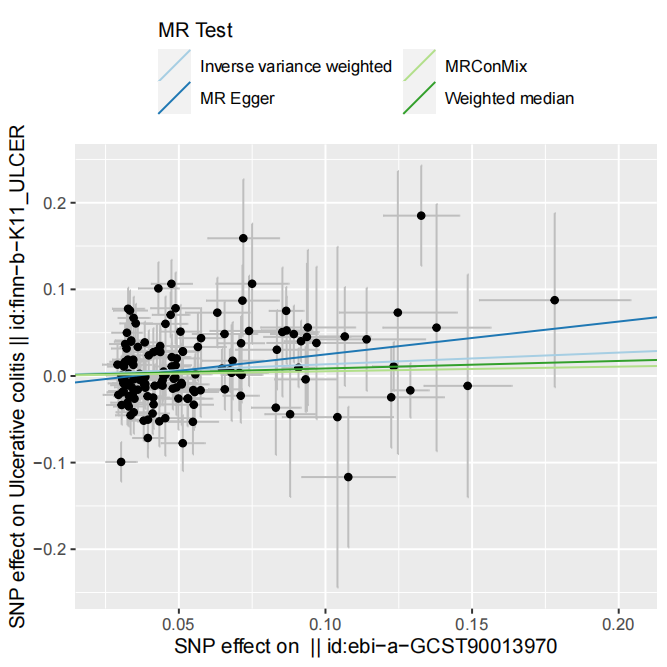


Supplementary Figure 9. Scatter plots

Contamination mixture.AR on UC.

**Supplementary material**


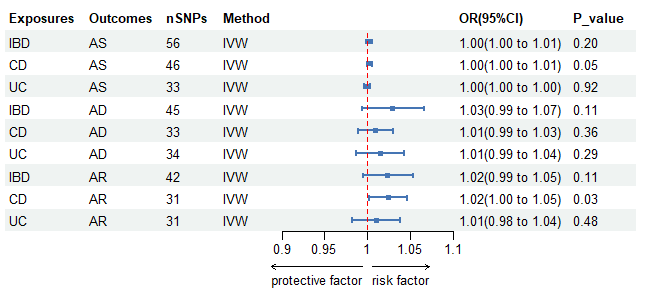


Supplementary Figure 10. Forest plots

Mendelian randomization analysis of inflammatory bowel disease (including crohn's disease and ulcerative colitis) as an exposure and atopic diseases (including asthma, atopic dermatitis, and allergic rhinitis) as outcomes.
