# Supplementary material for: Atopic disease and inflammatory bowel disease: A bidirectional Mendelian randomization study
Source: Medicine (Baltimore). 2024 Oct 18;103(42):e40143. doi: 10.1097/MD.0000000000040143 (PMC11495711; doi:10.1097/MD.0000000000040143)
Supplement: Supplementary file 3 [file medi-103-e40143-s003.docx]

**Table19 Pleiotropy and heterogeneity test results of all two-sample MR analyses**

| **pleiotropy_test** | | | **heterogeneity_test** | | | | | |
| --- | --- | --- | --- | --- | --- | --- | --- | --- |
| **exposure** | **outcome** | **p_value** | **method** | **Q statistic** | **p_value** | **method** | **Q statistic** | **p_value** |
| AR | IBD | 0.418 | MR-Egger | 2.49E+02 | ＜0.001 | IVW | 2.50E+02 | ＜0.001 |
| AR | CD | 0.940 | MR-Egger | 2.02E+02 | ＜0.001 | IVW | 2.02E+02 | ＜0.001 |
| AR | UC | 0.109 | MR-Egger | 2.33E+02 | ＜0.001 | IVW | 2.38E+02 | ＜0.001 |
| Asthma | IBD | 0.503 | MR-Egger | 2.42E+02 | ＜0.001 | IVW | 2.44E+02 | ＜0.001 |
| Asthma | CD | 0.483 | MR-Egger | 2.12E+02 | ＜0.001 | IVW | 2.14E+02 | ＜0.001 |
| Asthma | UC | 0.917 | MR-Egger | 1.48E+02 | ＜0.001 | IVW | 1.48E+02 | ＜0.001 |
| AD | IBD | 0.672 | MR-Egger | 1.04E+02 | ＜0.001 | IVW | 1.04E+02 | ＜0.001 |
| AD | CD | 0.248 | MR-Egger | 7.78E+01 | 0.051 | IVW | 7.96E+01 | 0.046 |
| AD | UC | 0.678 | MR-Egger | 1.11E+02 | ＜0.001 | IVW | 1.12E+02 | ＜0.001 |
| IBD | AR | 0.420 | MR-Egger | 2.56E+02 | ＜0.001 | IVW | 2.60E+02 | ＜0.001 |
| CD | AR | 0.570 | MR-Egger | 1.43E+02 | ＜0.001 | IVW | 1.45E+02 | ＜0.001 |
| UC | AR | 0.777 | MR-Egger | 1.32E+02 | ＜0.001 | IVW | 1.32E+02 | ＜0.001 |
| IBD | Asthma | 0.125 | MR-Egger | 2.21E+02 | ＜0.001 | IVW | 2.31E+02 | ＜0.001 |
| CD | Asthma | 0.109 | MR-Egger | 1.83E+02 | ＜0.001 | IVW | 1.94E+02 | ＜0.001 |
| UC | Asthma | 0.004 | MR-Egger | 9.26E+01 | ＜0.001 | IVW | 1.22E+02 | ＜0.001 |
| IBD | AD | 0.149 | MR-Egger | 8.24E+01 | ＜0.001 | IVW | 8.65E+01 | ＜0.001 |
| CD | AD | 0.721 | MR-Egger | 3.30E+01 | 0.369 | IVW | 3.32E+01 | 0.410 |
| UC | AD | 0.571 | MR-Egger | 3.11E+01 | 0.513 | IVW | 3.14E+01 | 0.546 |

AR, allergic rhinitis

IVW, inverse variance weighting

IBD, inflammatory bowel disease

CD, Crohn's disease

UC, ulcerative colitis

AD, atopic dermatitis
